# Supplementary material for: Drivers of linkage disequilibrium across a species’ geographic range
Source: PLoS Genet. 2021 Mar 26;17(3):e1009477. doi: 10.1371/journal.pgen.1009477 (PMC8026057; doi:10.1371/journal.pgen.1009477)
Supplement: S1 Fig — Decay of linkage disequilibrium (LD), measured as r2 between SNPs, with increasing physical distance (bps) for genic (black) and intergenic (blue) regions for each population plotted separately (S1 Table). LD is based on the average across all retained pairwise LD estimates for each base-pair distance bin. Note that with increasing insert size, fewer observations occur, increasing the stochasticity in the plots. (PDF) [file pgen.1009477.s001.pdf]

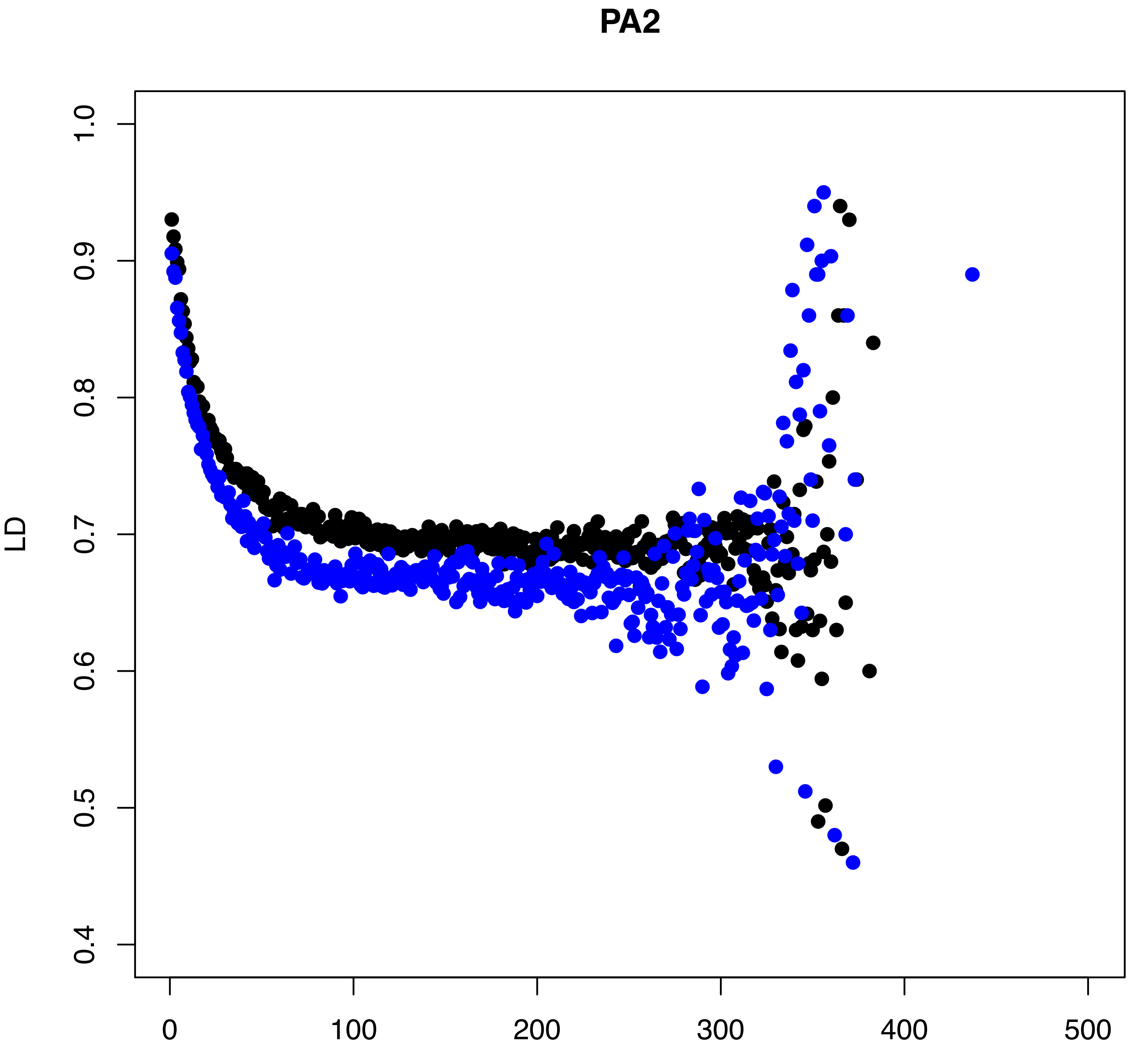
PA2
LD
1.0
0.9
0.8
0.7
0.6
0.5
0.4
0
100
200
300
400
500

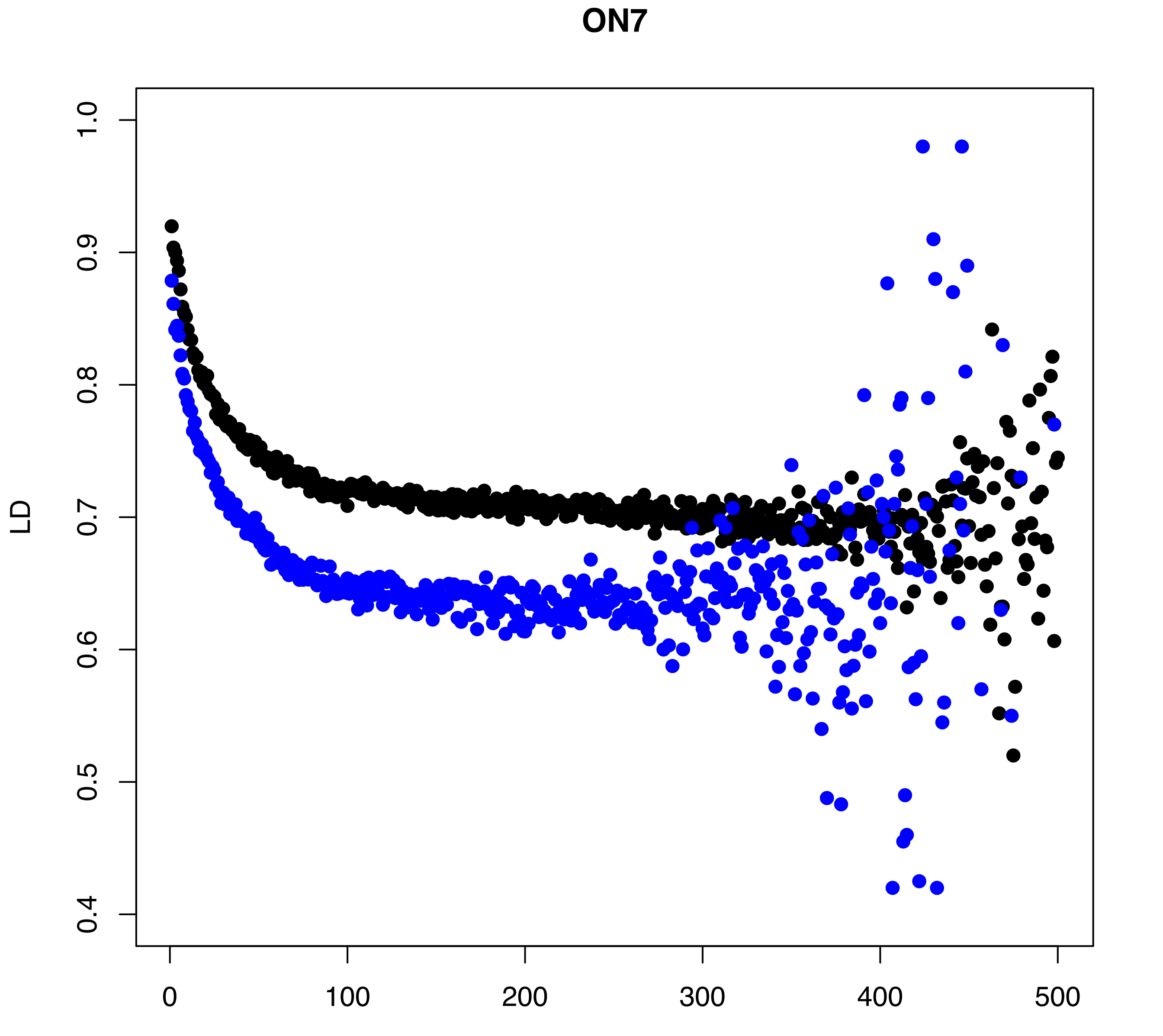

ON7
LD
1.0
0.9
0.8
0.7
0.6
0.5
0.4
0
100
200
300
400
500

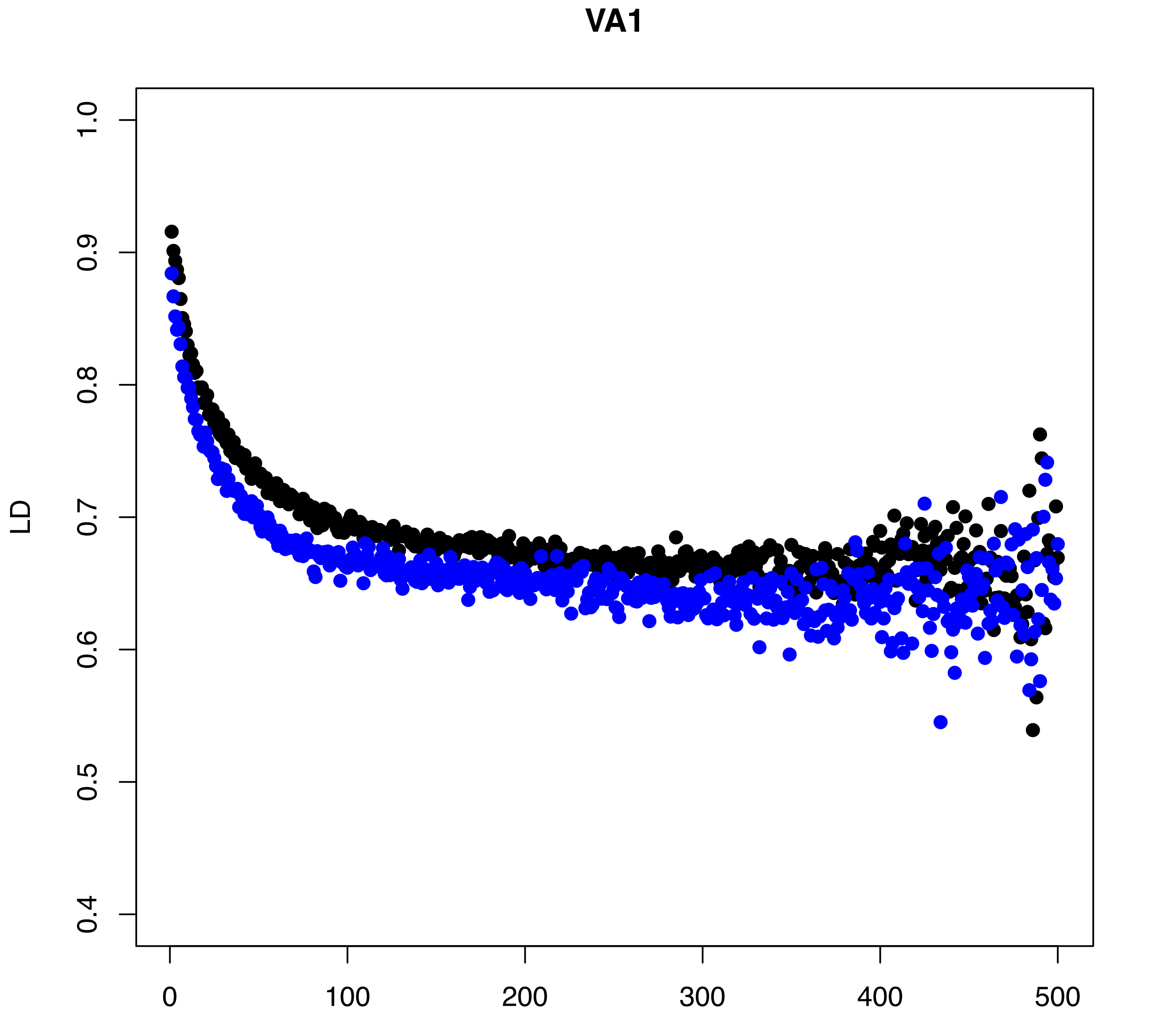

VA1
LD
1.0
0.9
0.8
0.7
0.6
0.5
0.4
0
100
200
300
400
500

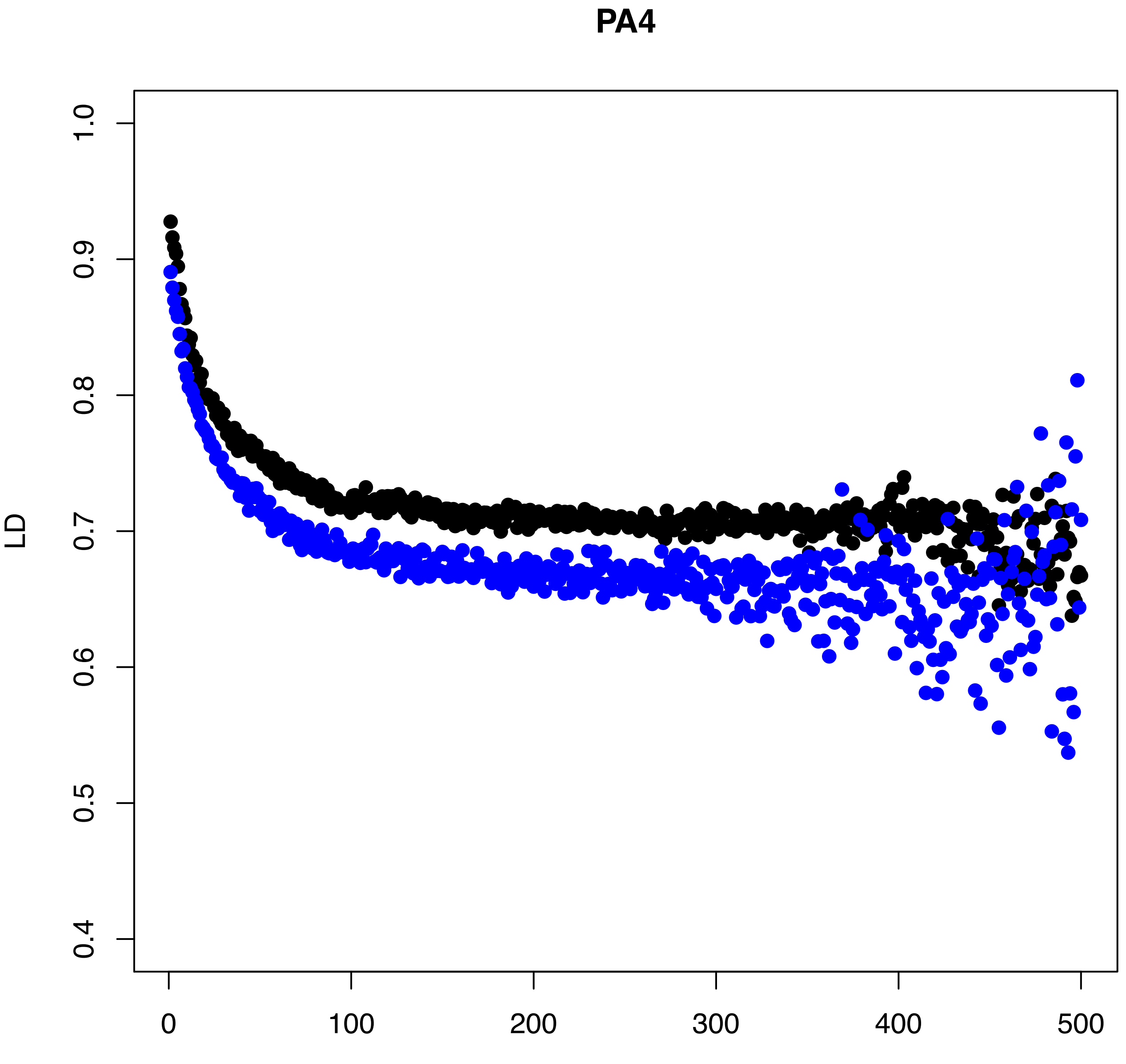
PA4
LD
1.0
0.9
0.8
0.7
0.6
0.5
0.4
0
100
200
300
400
500

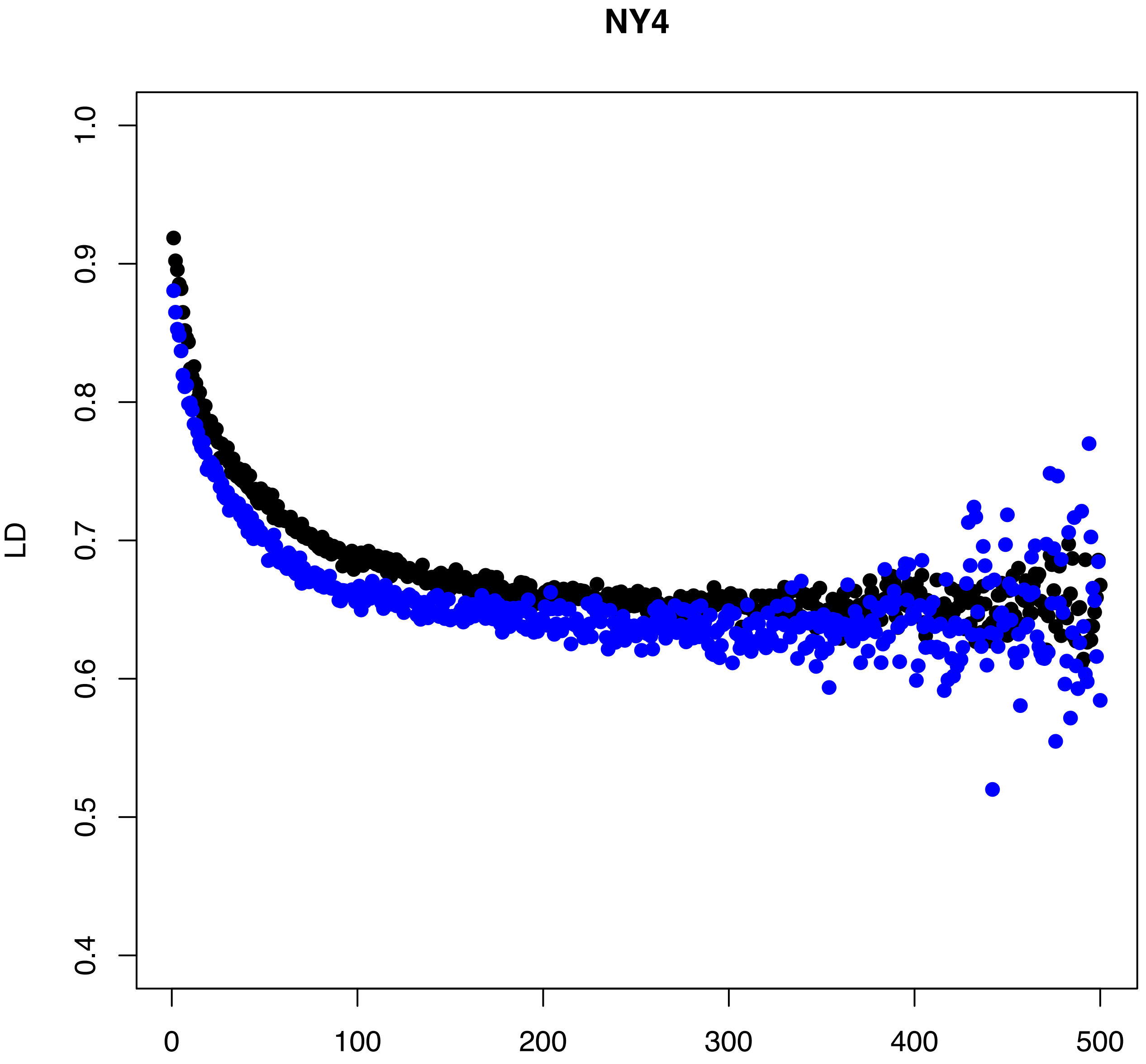

NY4
LD
1.0
0.9
0.8
0.7
0.6
0.5
0.4
0
100
200
300
400
500

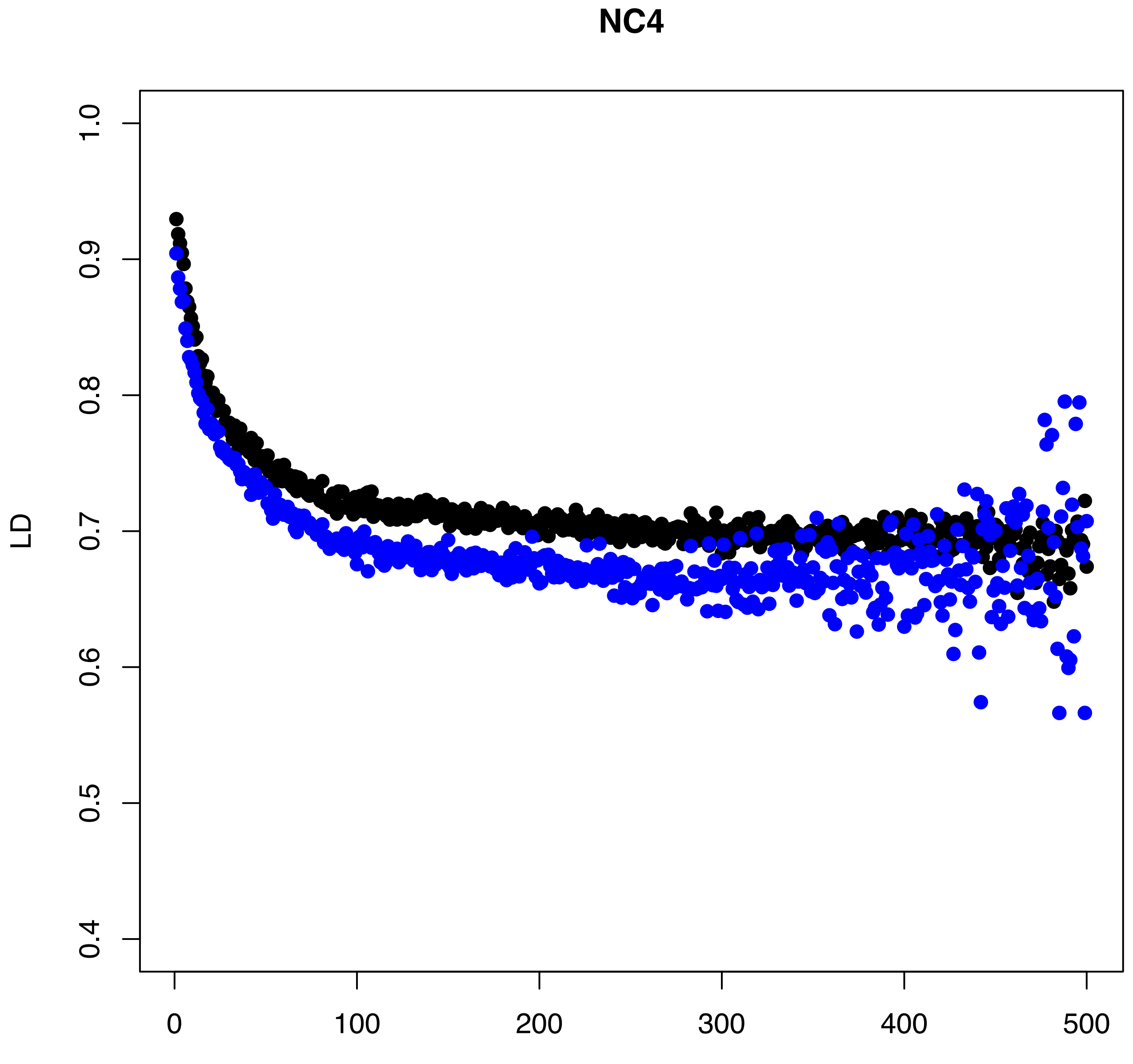
NC4
LD
1.0
0.9
0.8
0.7
0.6
0.5
0.4
0
100
200
300
400
500

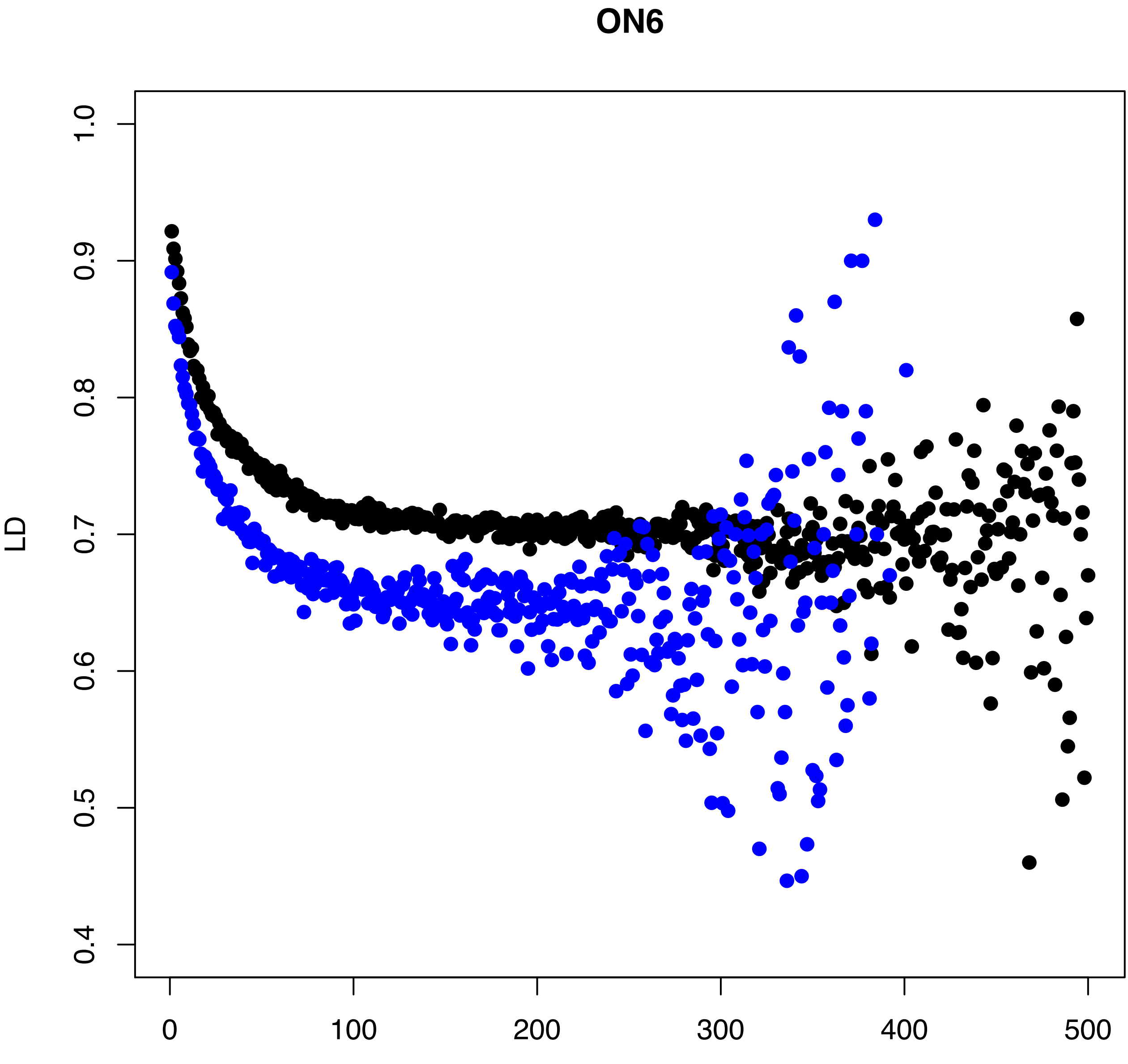

ON6
LD
1.0
0.9
0.8
0.7
0.6
0.5
0.4
0
100
200
300
400
500

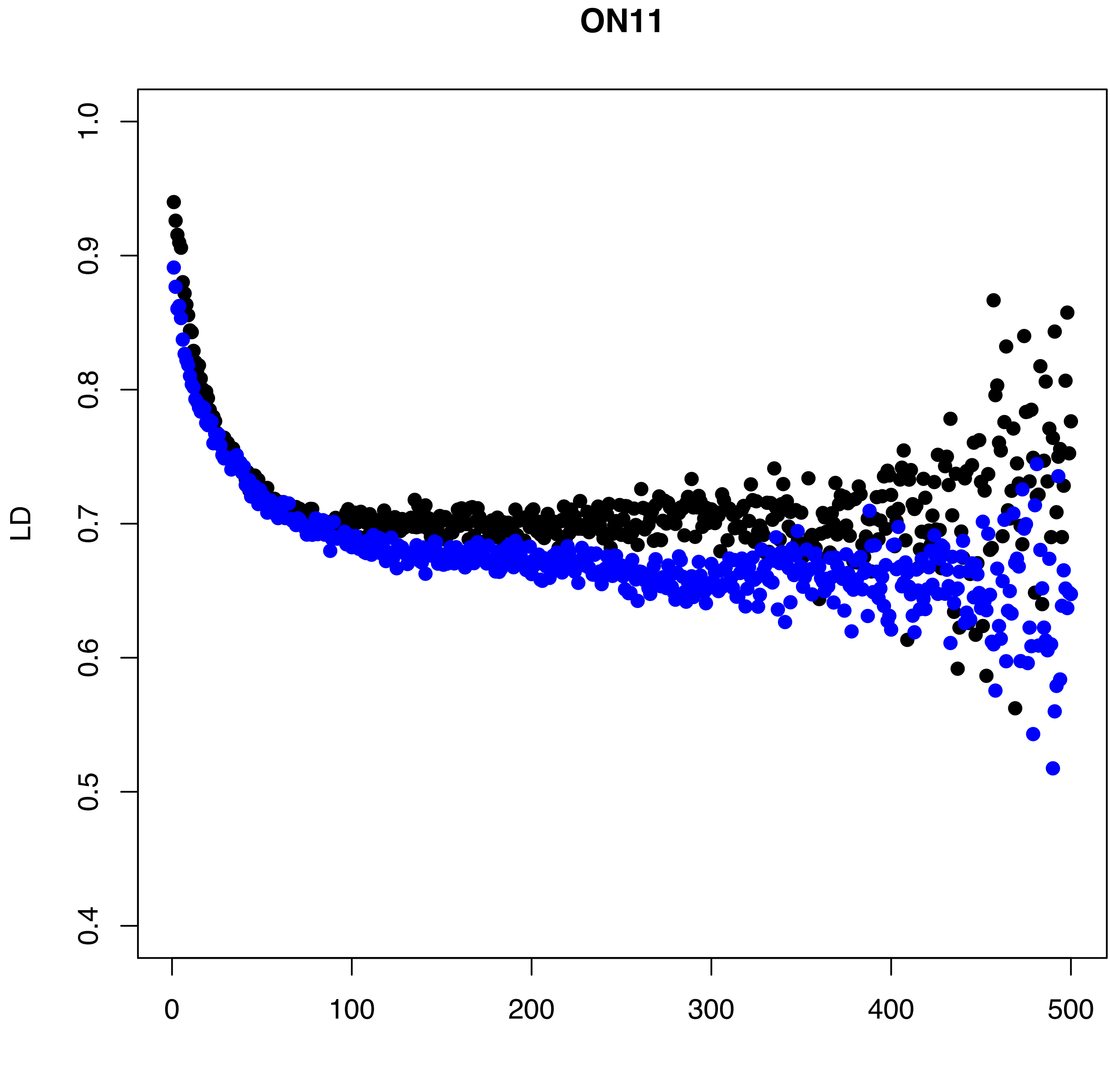
ON11
LD
1.0
0.9
0.8
0.7
0.6
0.5
0.4
0
100
200
300
400
500

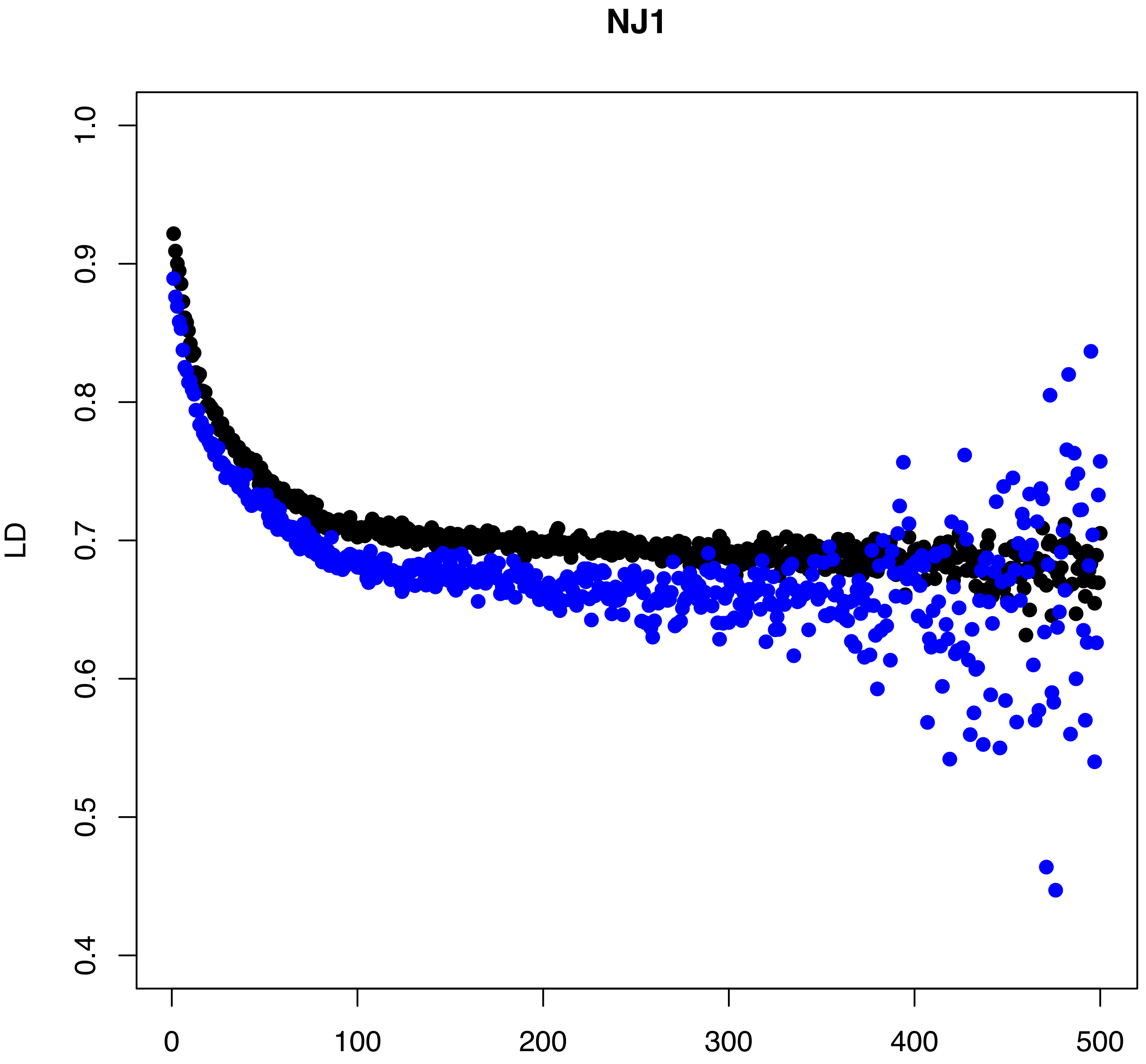
NJ1
LD
1.0
0.9
0.8
0.7
0.6
0.5
0.4
0
100
200
300
400
500

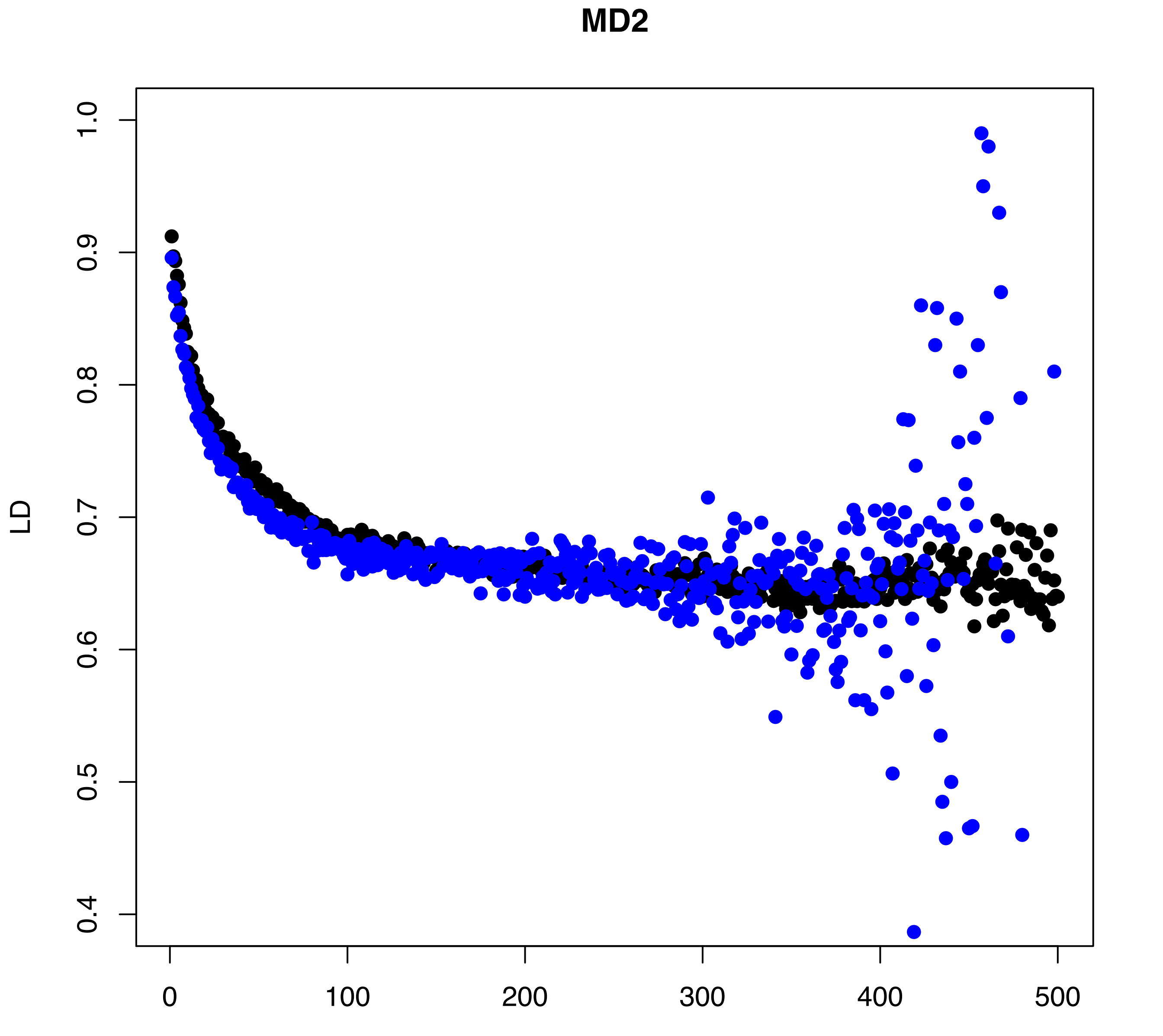
MD2
LD
1.0
0.9
0.8
0.7
0.6
0.5
0.4
0
100
200
300
400
500

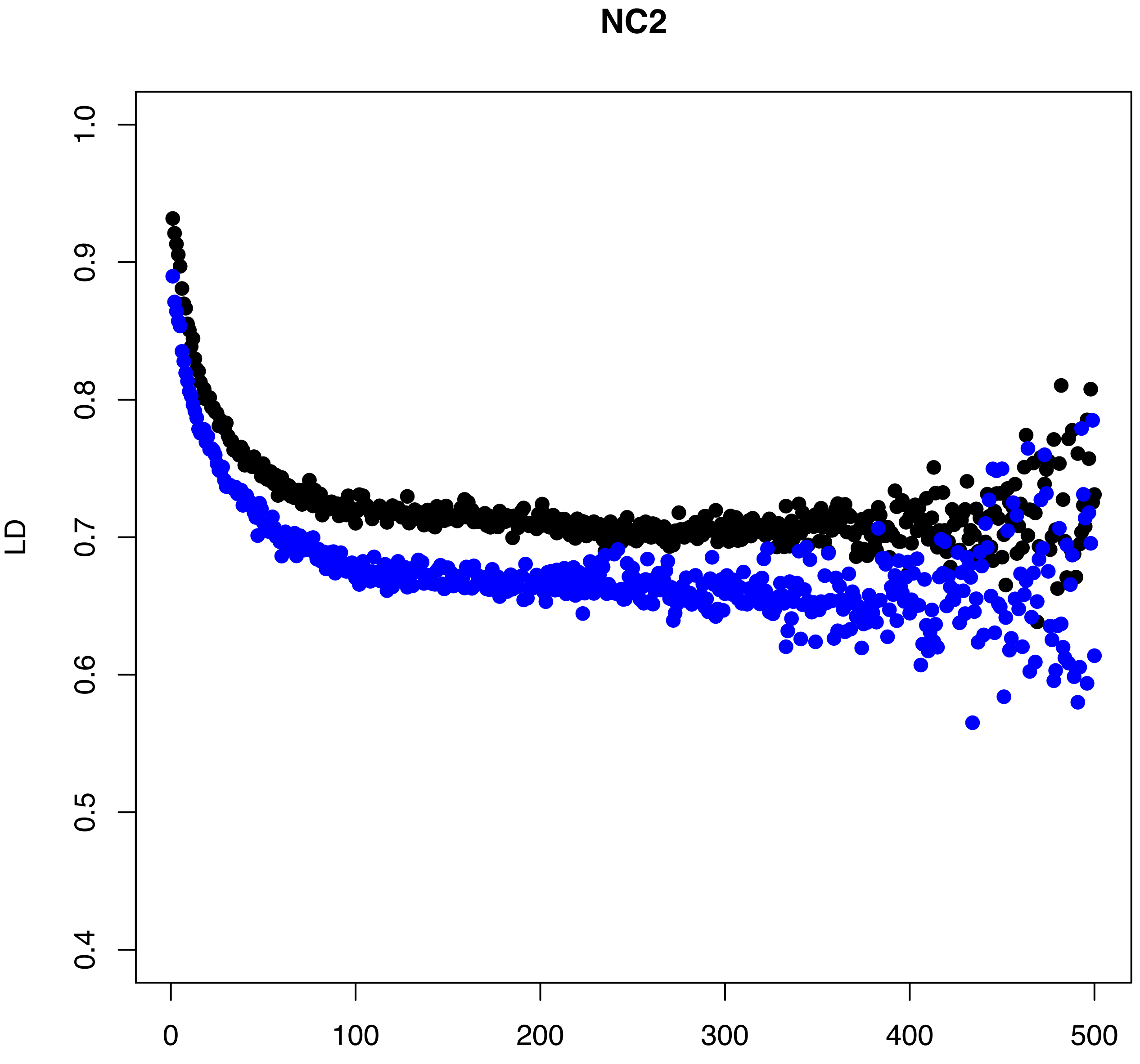
NC2
LD
1.0
0.9
0.8
0.7
0.6
0.5
0.4
0
100
200
300
400
500

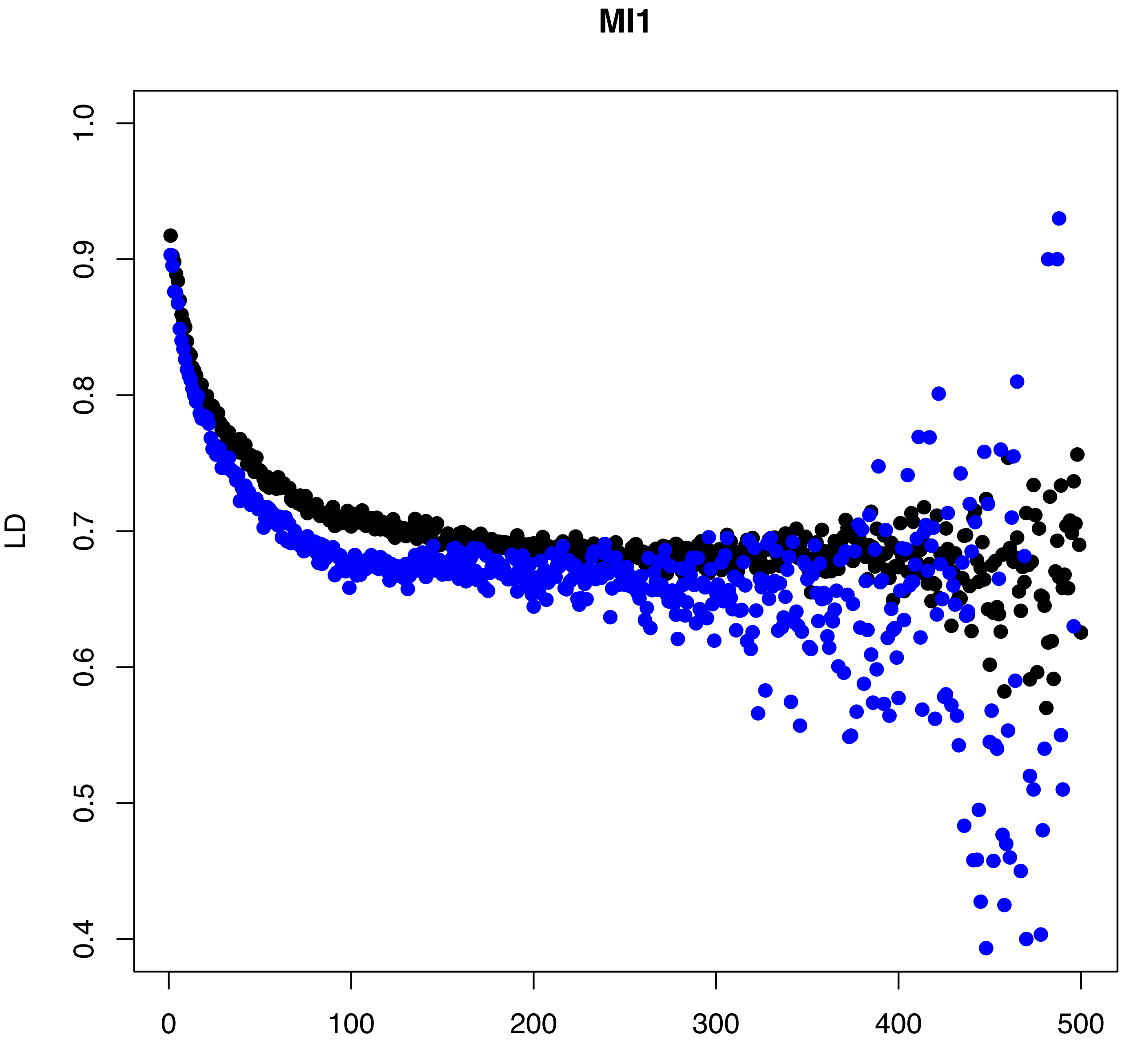
MI1
LD
1.0
0.9
0.8
0.7
0.6
0.5
0.4
0
100
200
300
400
500

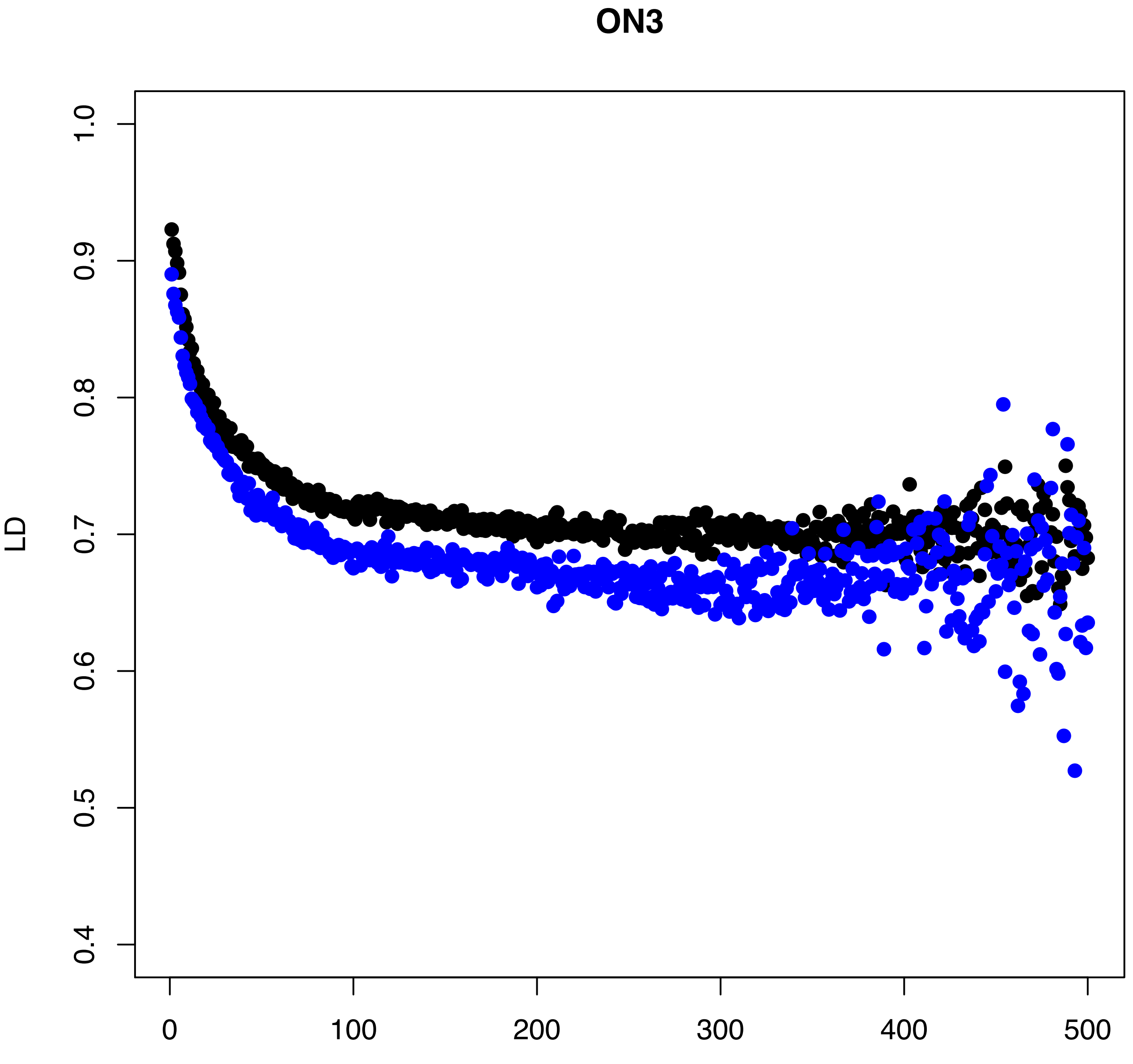
ON3
LD
0.4
0.5
0.6
0.7
0.8
0.9
1.0
0
100
200
300
400
500

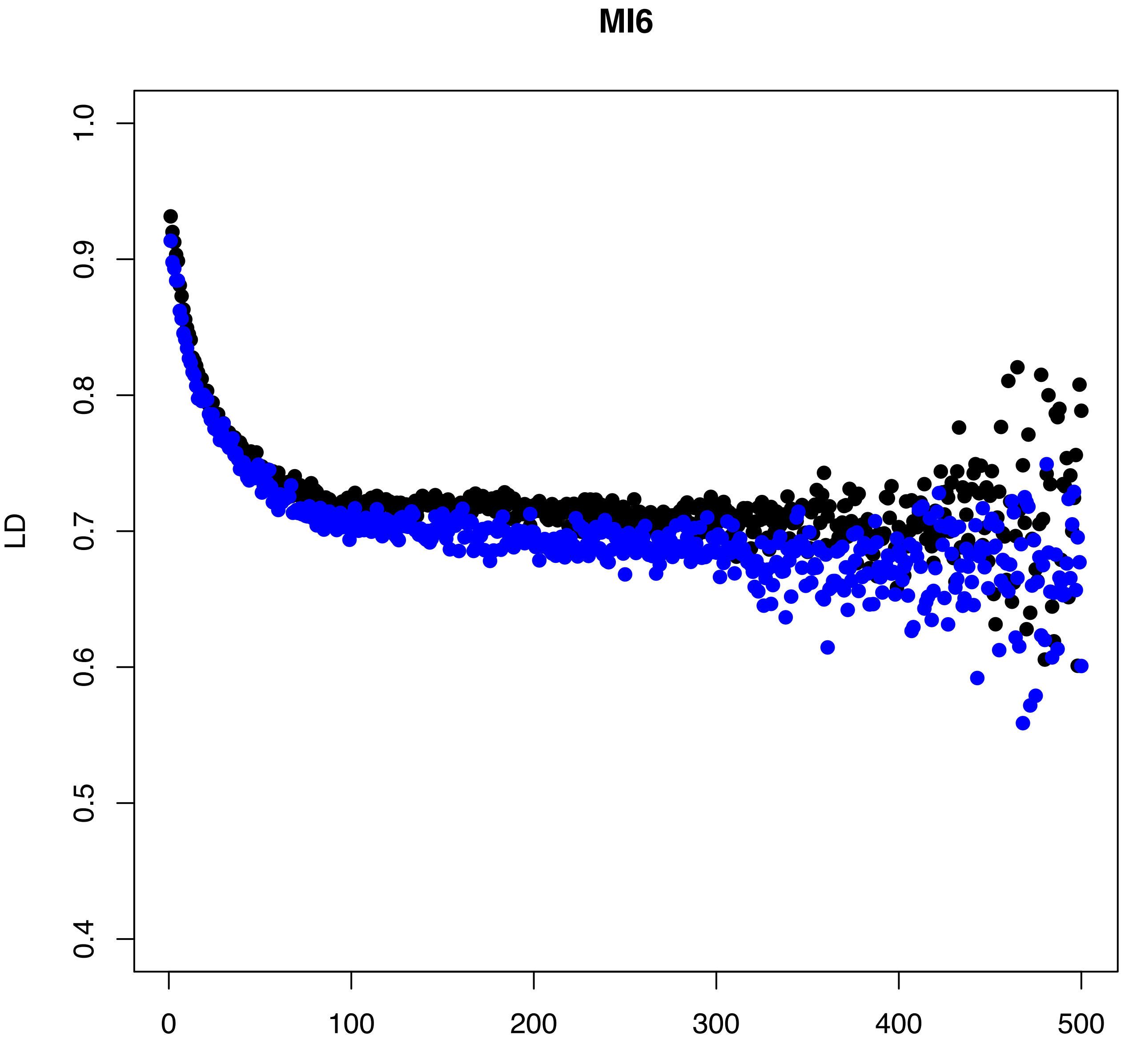
MI6
LD
1.0
0.9
0.8
0.7
0.6
0.5
0.4
0
100
200
300
400
500

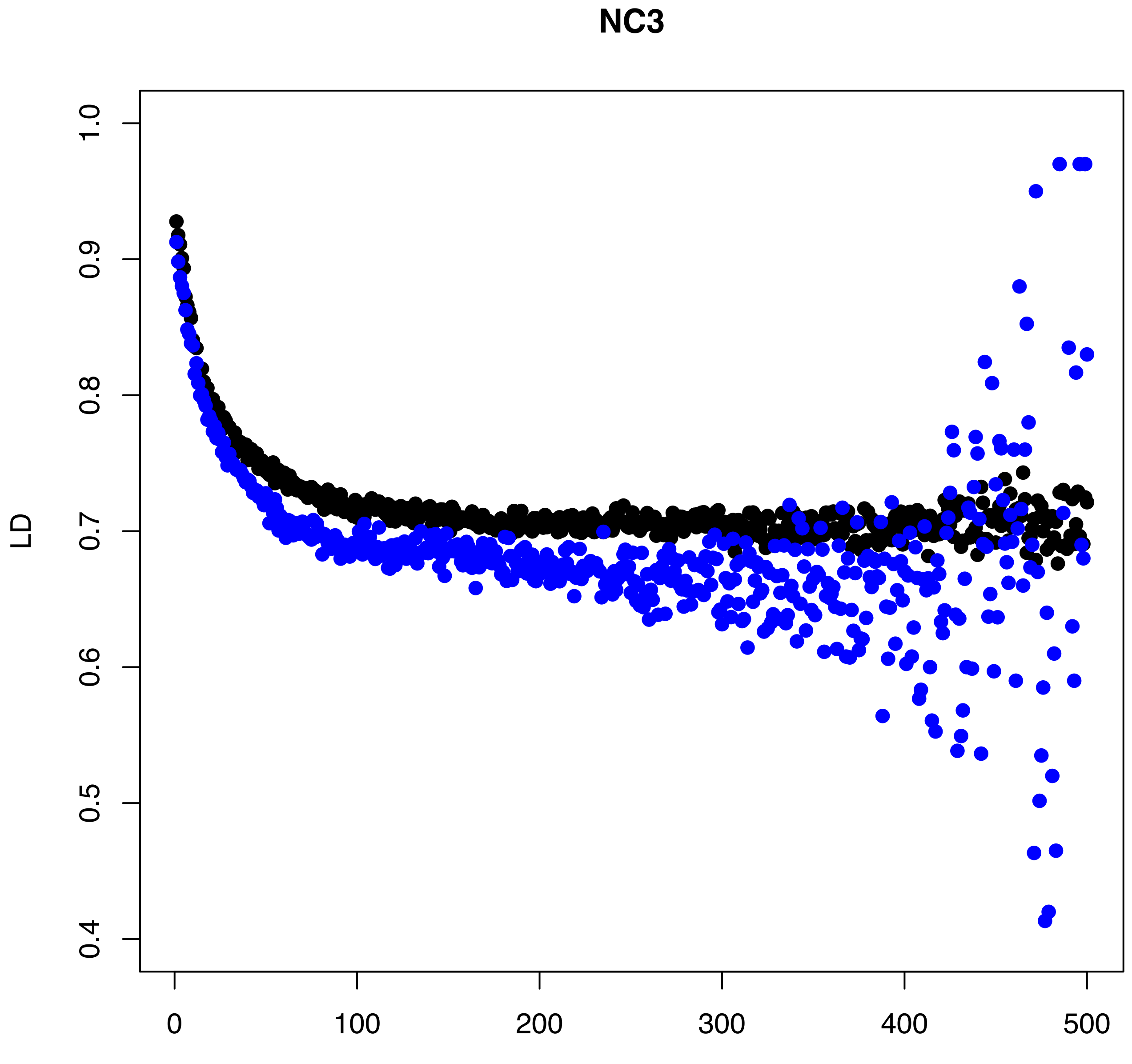
NC3
LD
1.0
0.9
0.8
0.7
0.6
0.5
0.4
0
100
200
300
400
500

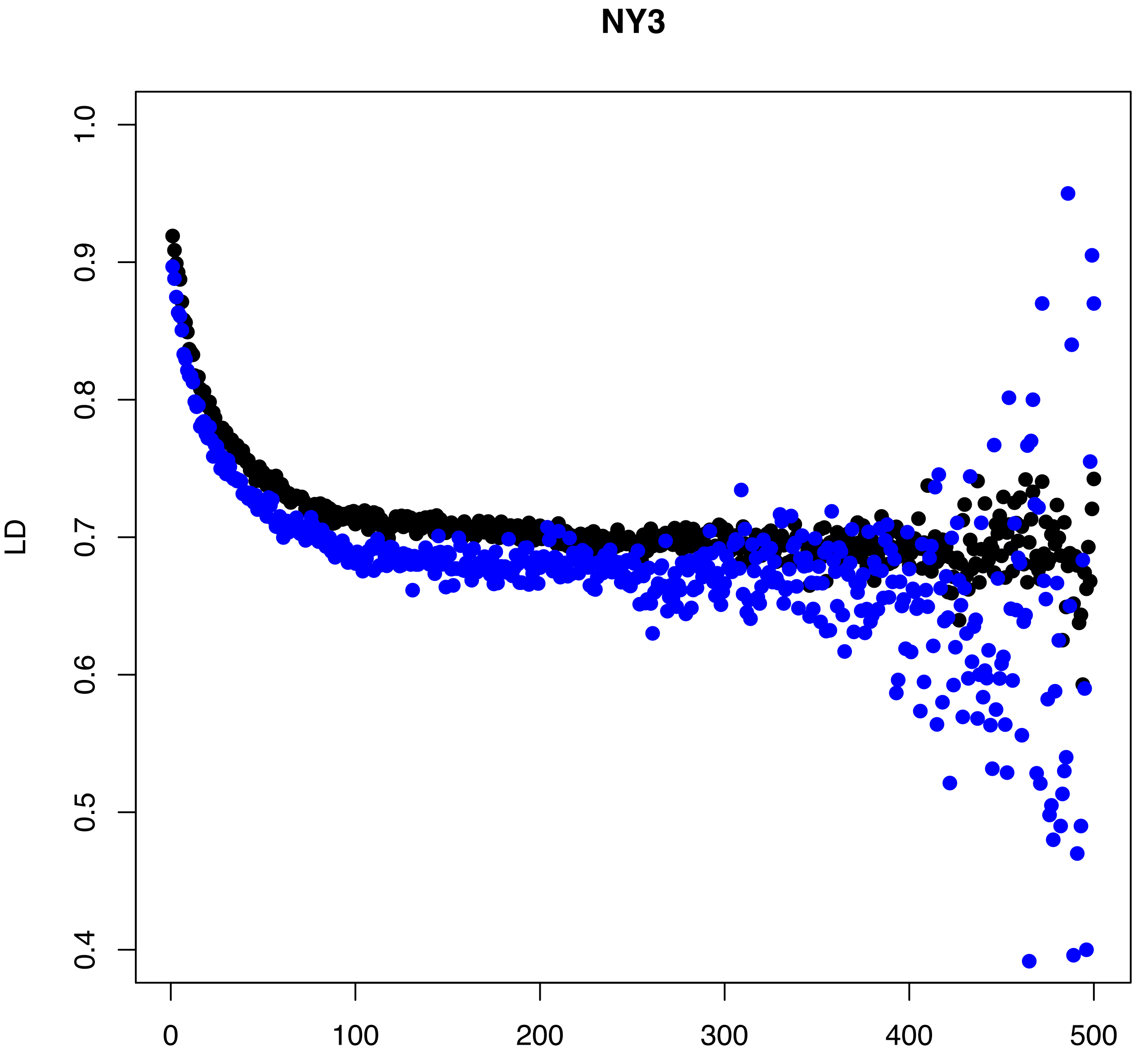

NY3
LD
1.0
0.9
0.8
0.7
0.6
0.5
0.4
0
100
200
300
400
500

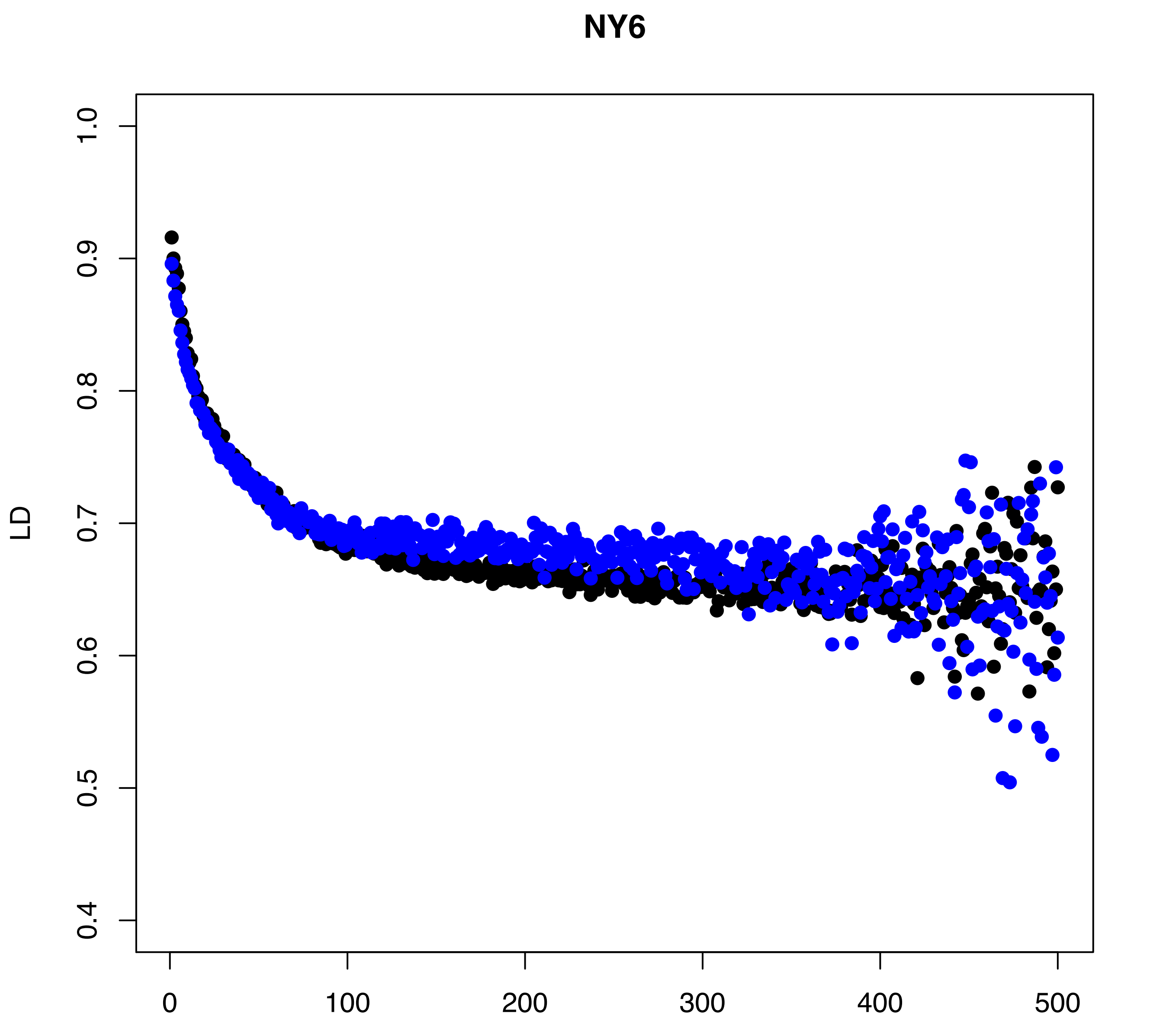
NY6
LD
1.0
0.9
0.8
0.7
0.6
0.5
0.4
0
100
200
300
400
500

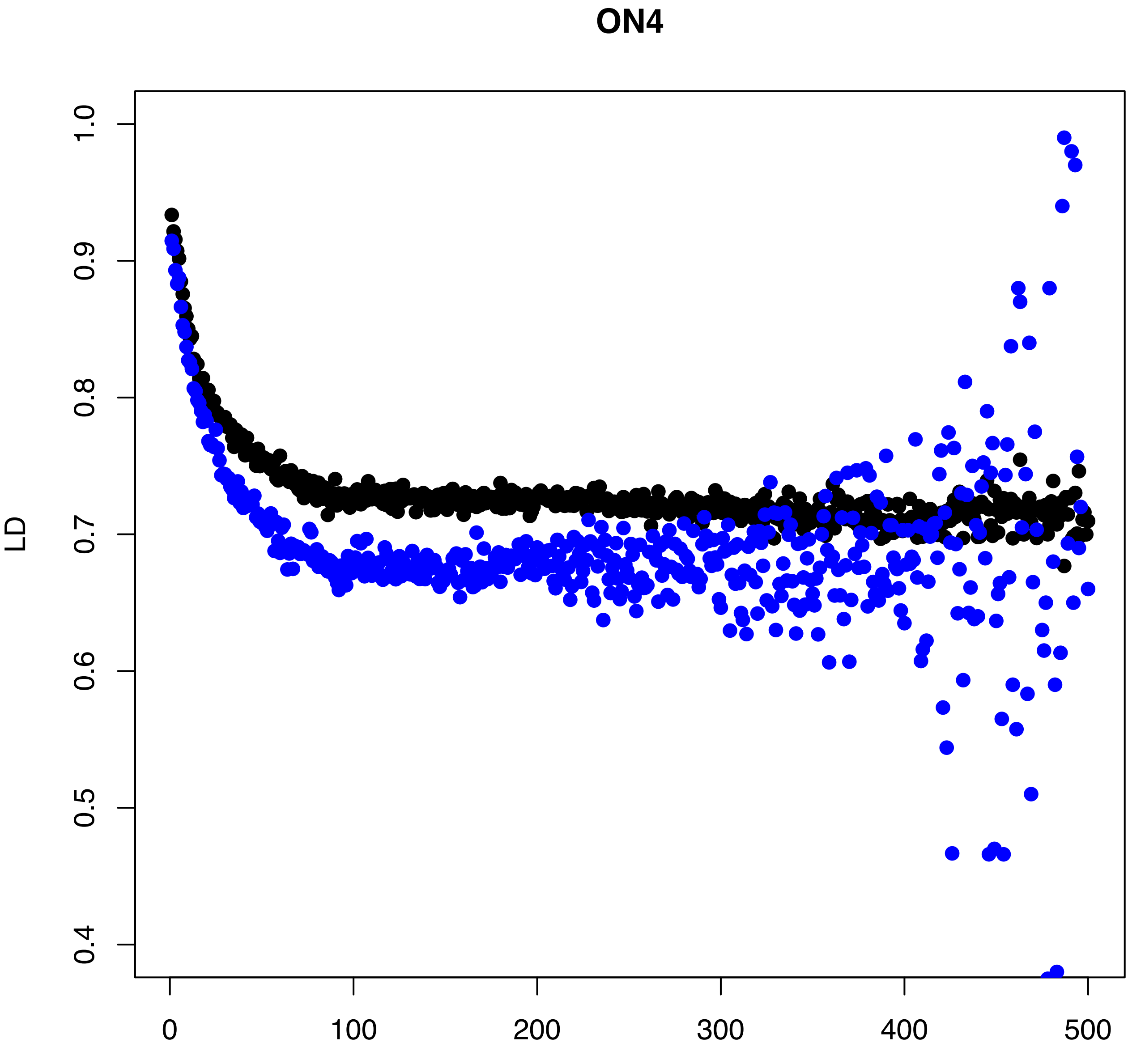
ON4
LD
1.0
0.9
0.8
0.7
0.6
0.5
0.4
0
100
200
300
400
500

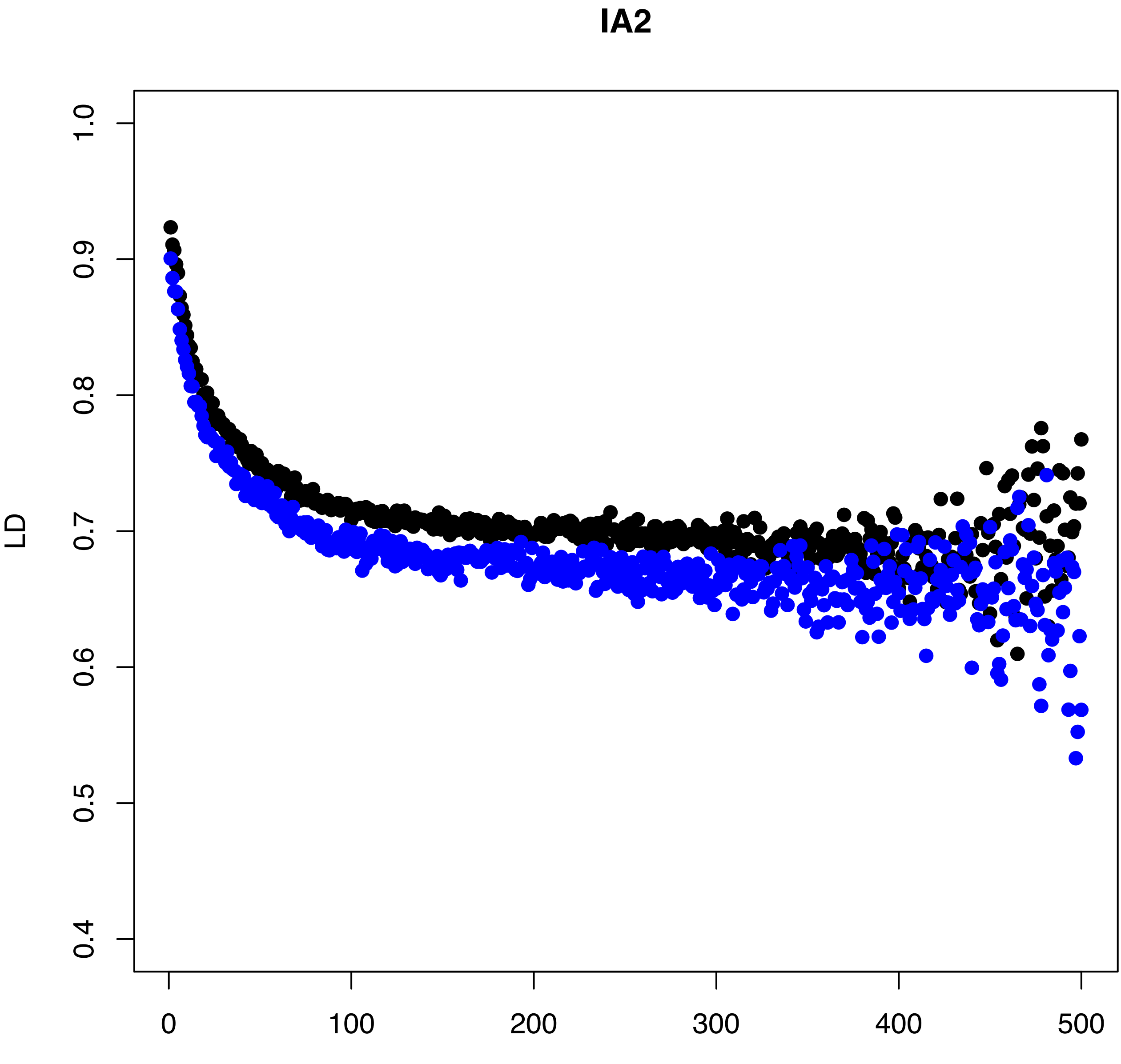
IA2
LD
1.0
0.9
0.8
0.7
0.6
0.5
0.4
0
100
200
300
400
500

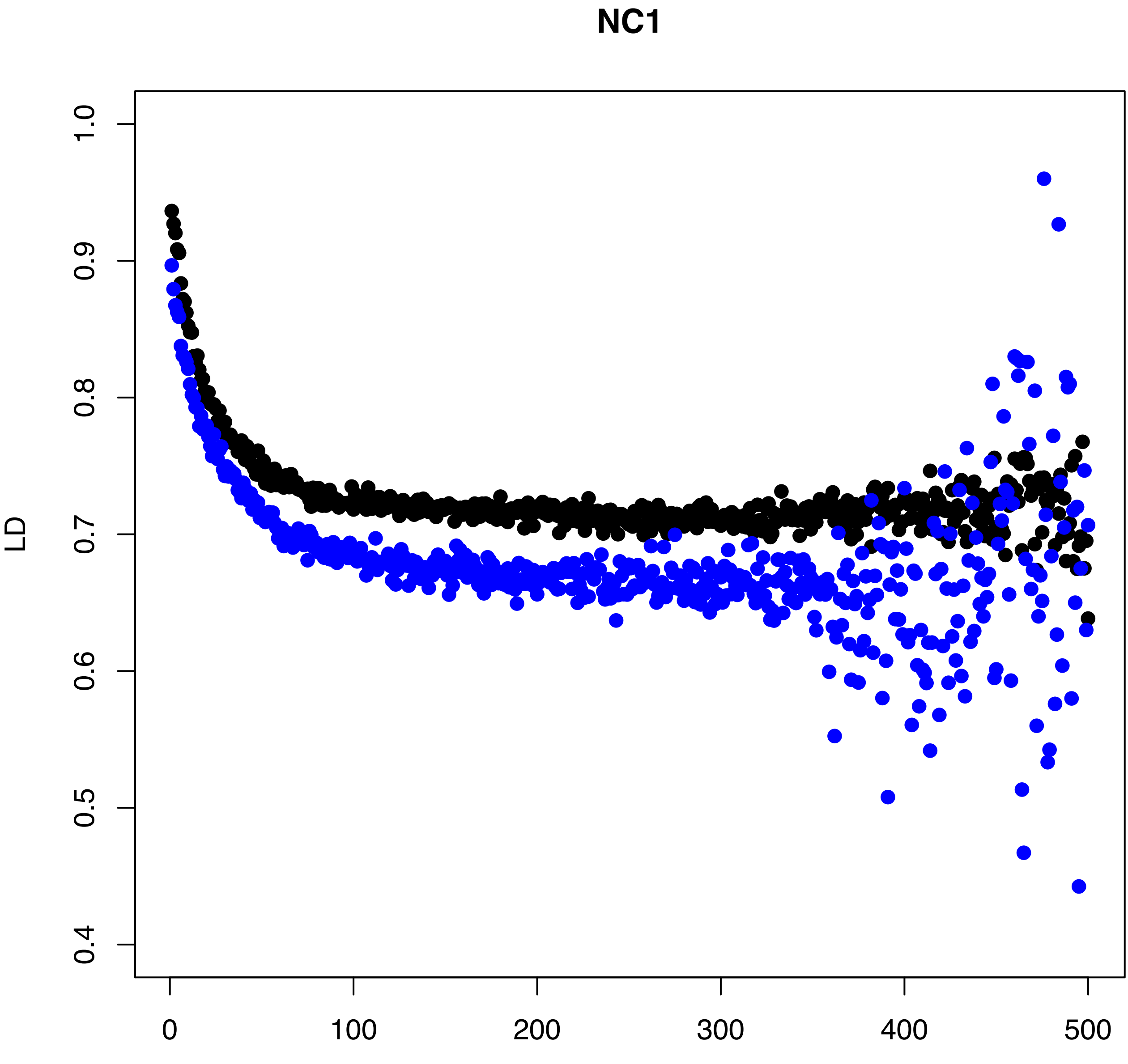
NC1
LD
1.0
0.9
0.8
0.7
0.6
0.5
0.4
0
100
200
300
400
500

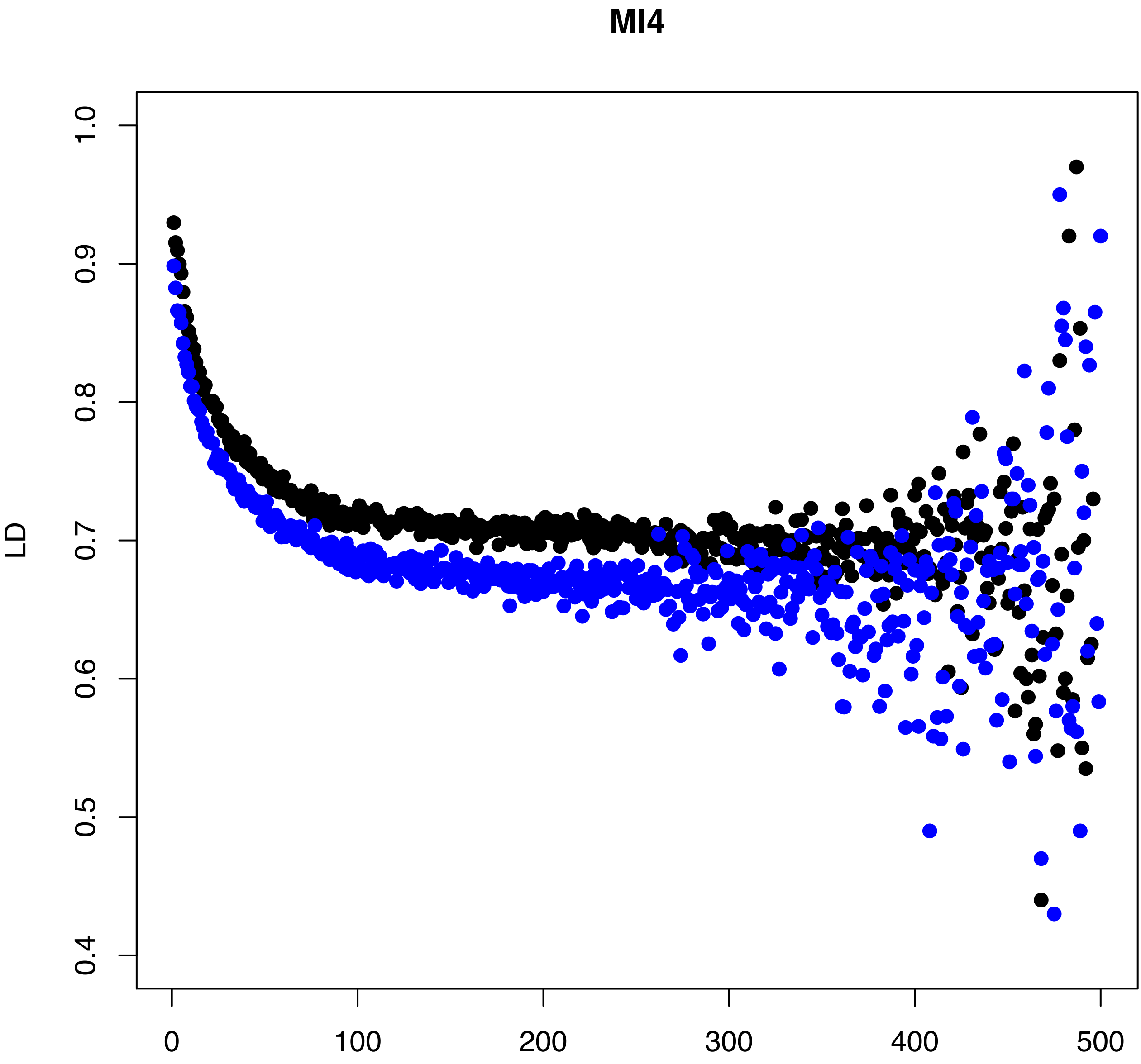

MI4
LD
1.0
0.9
0.8
0.7
0.6
0.5
0.4
0
100
200
300
400
500

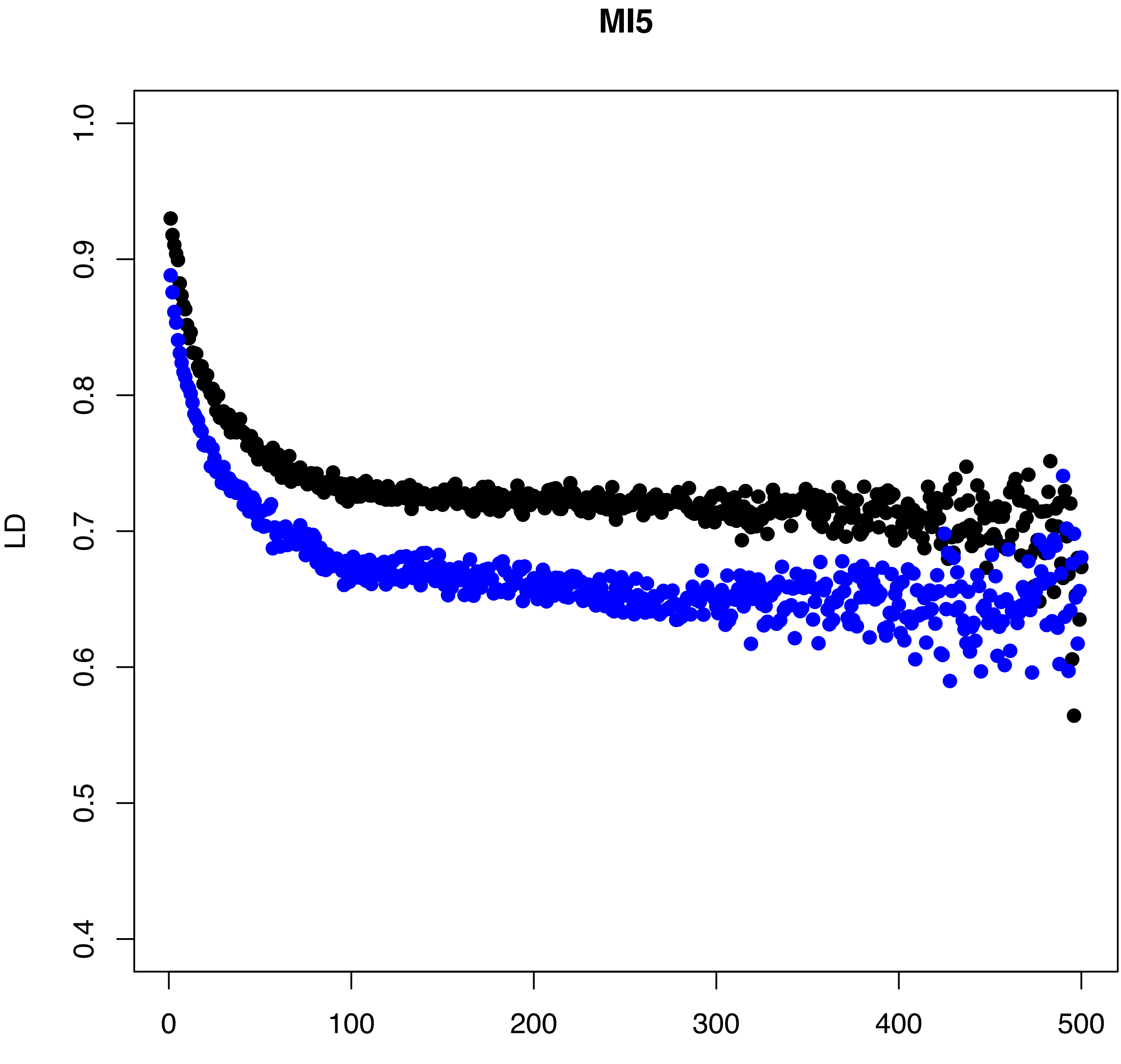
MI5
LD
1.0
0.9
0.8
0.7
0.6
0.5
0.4
0
100
200
300
400
500

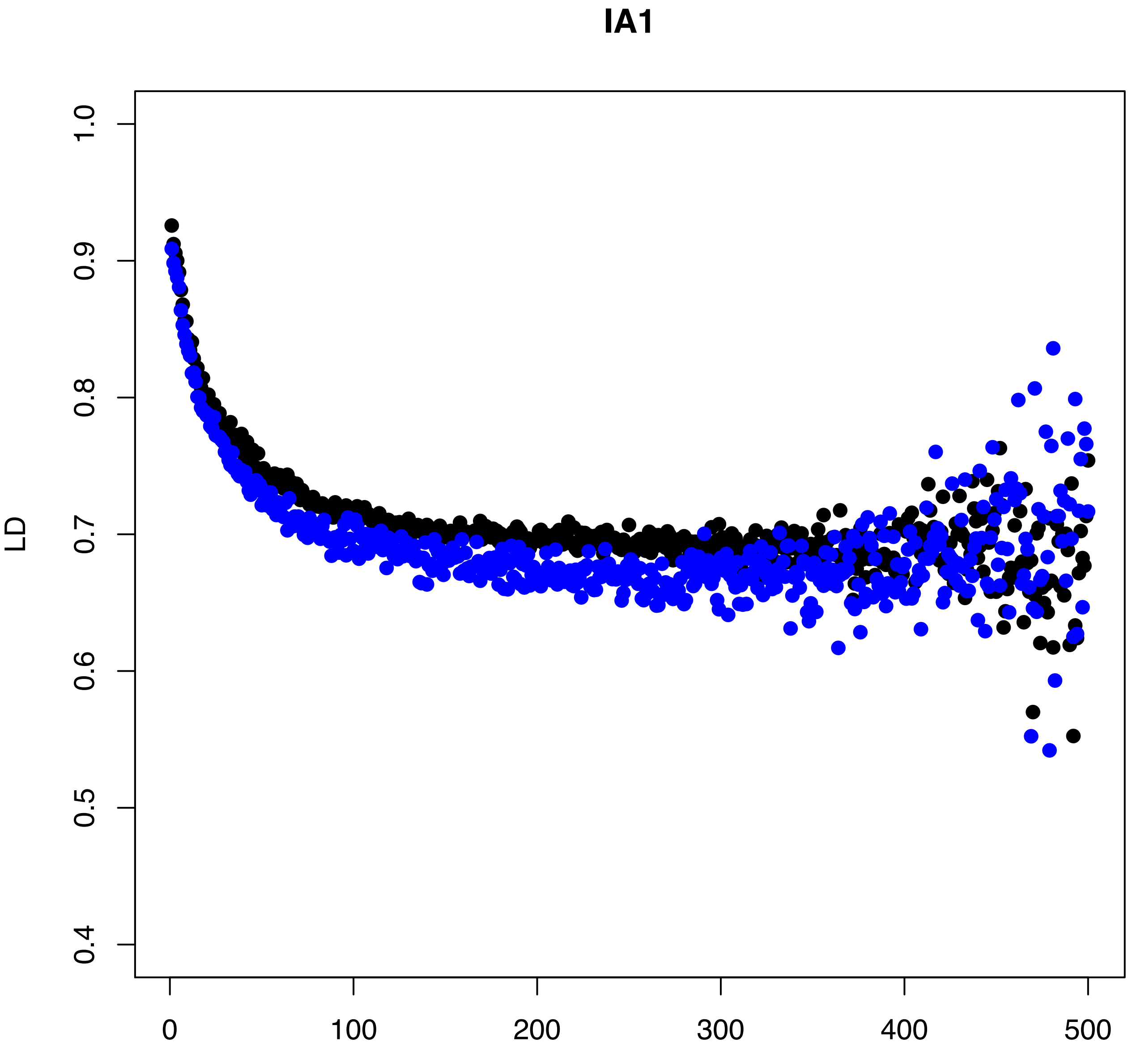
IA1
LD
1.0
0.9
0.8
0.7
0.6
0.5
0.4
0
100
200
300
400
500

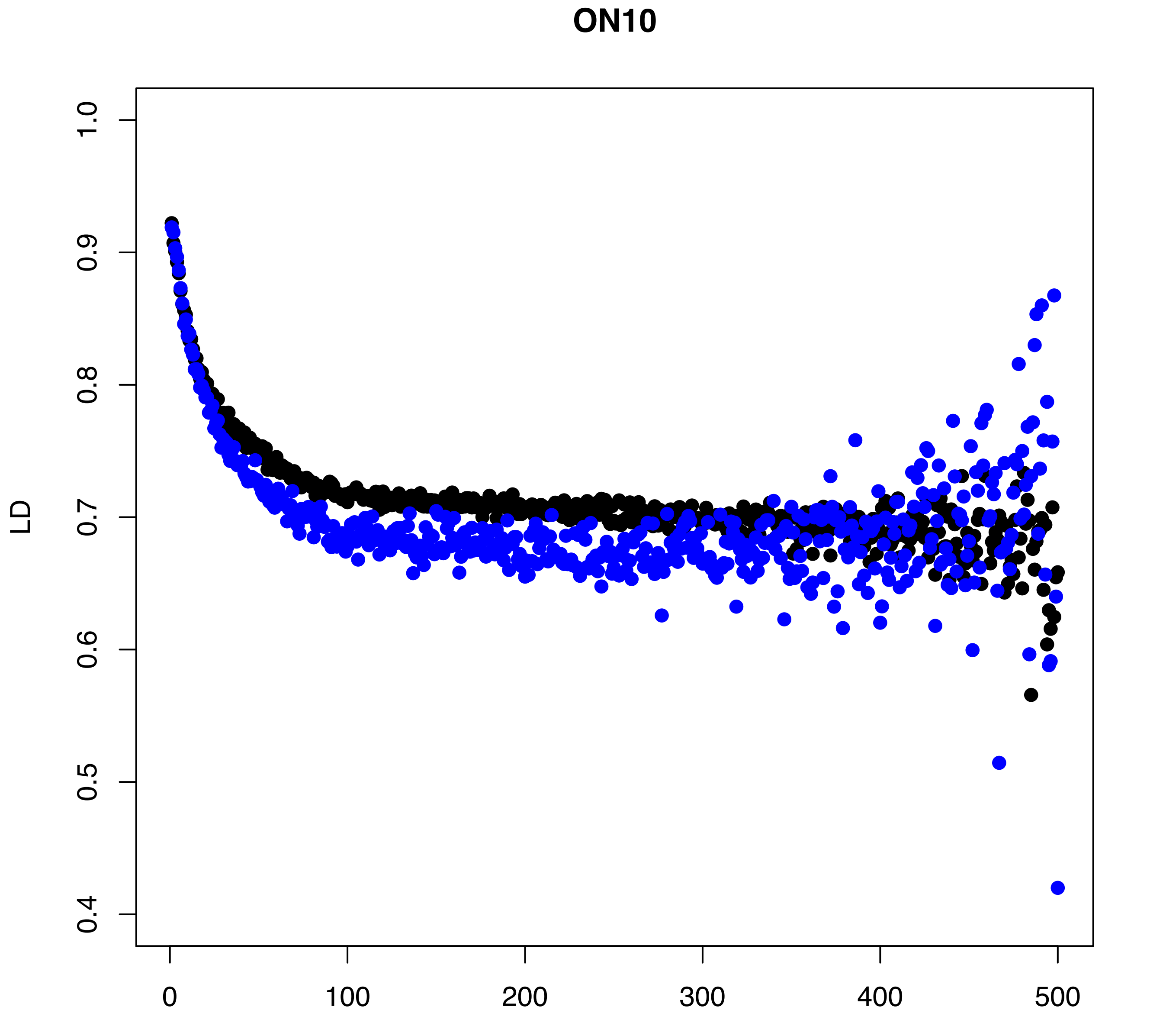
ON10
LD
1.0
0.9
0.8
0.7
0.6
0.5
0.4
0
100
200
300
400
500

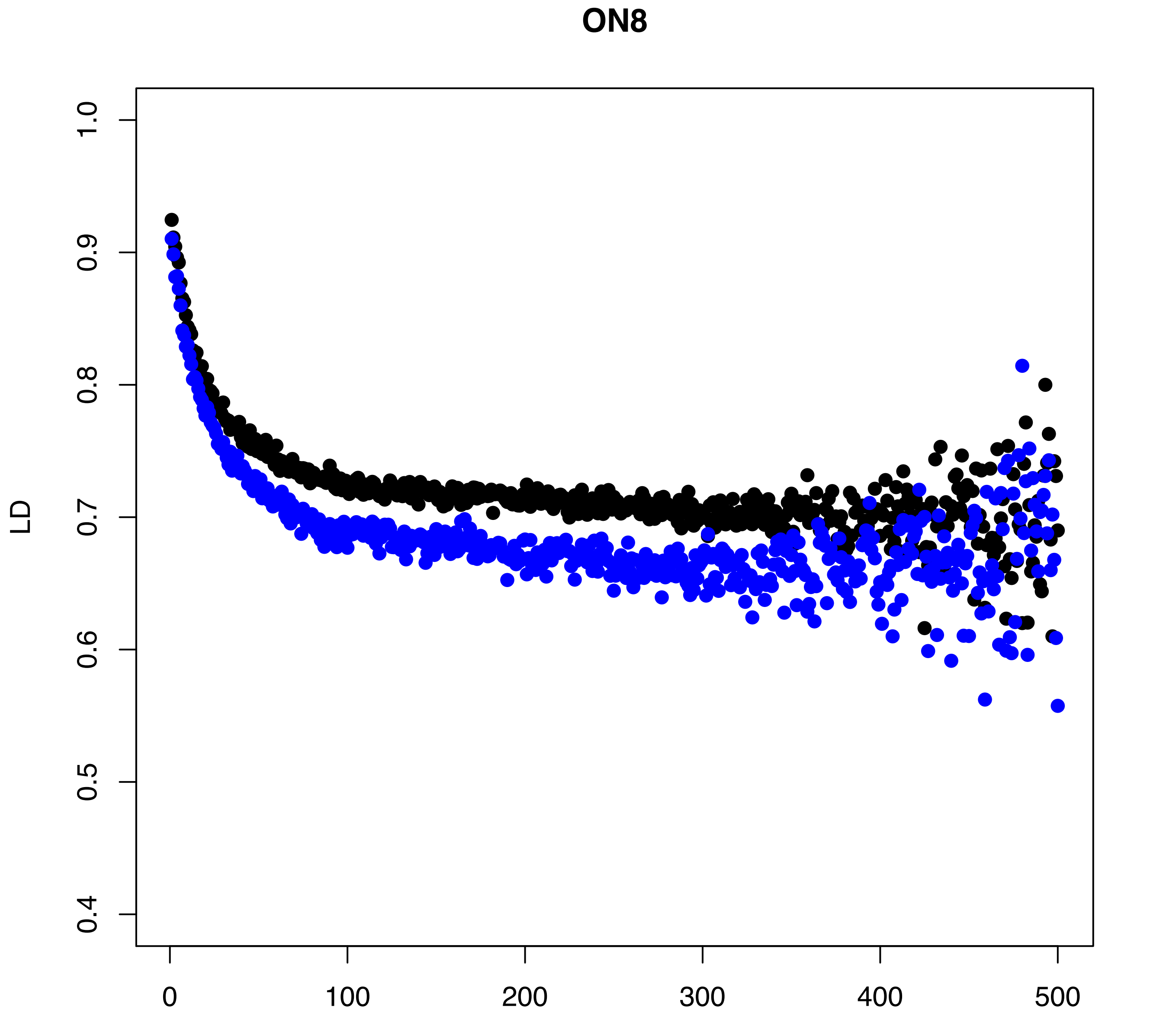
ON8
LD
1.0
0.9
0.8
0.7
0.6
0.5
0.4
0
100
200
300
400
500

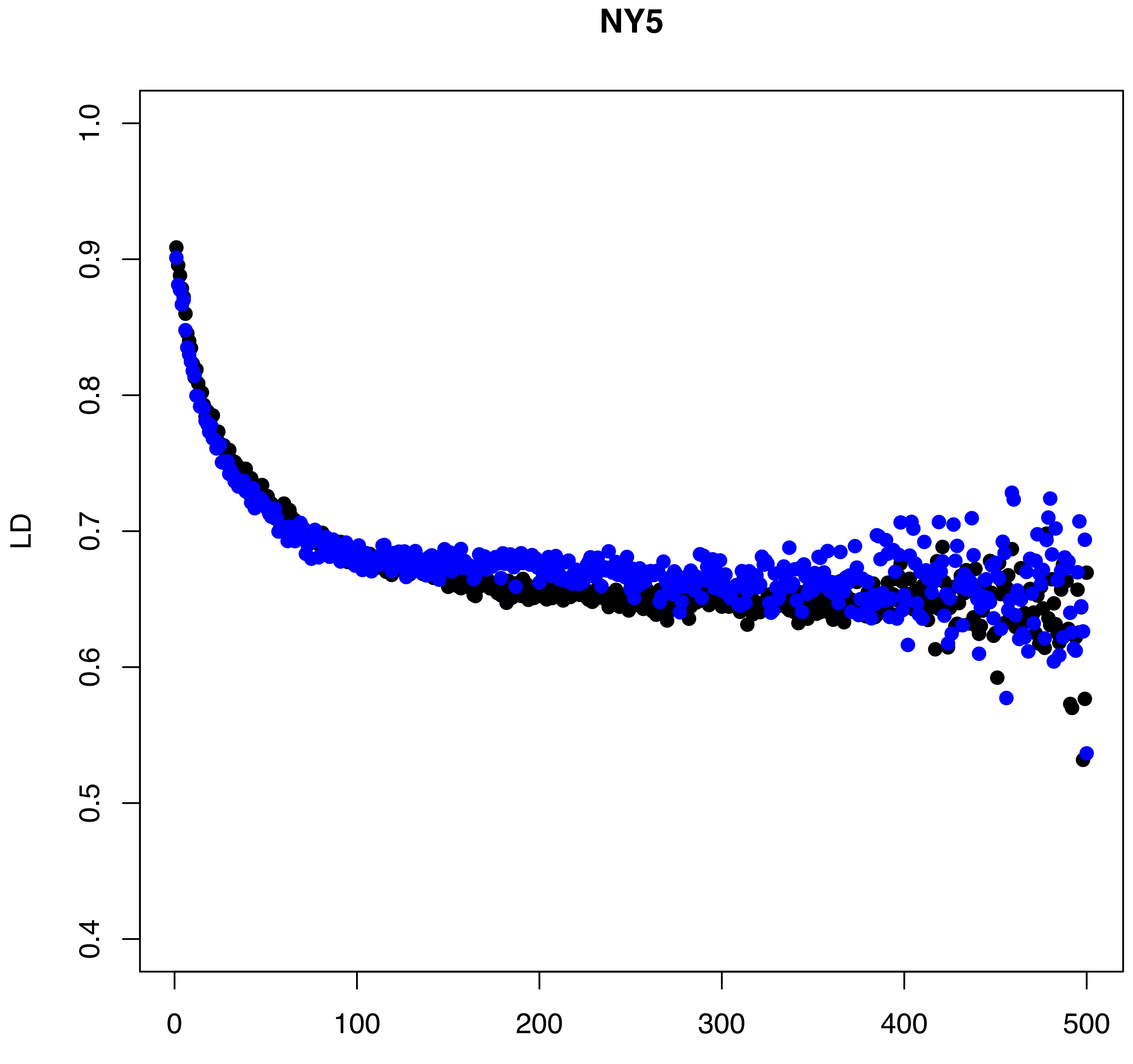
NY5
LD
1.0
0.9
0.8
0.7
0.6
0.5
0.4
0
100
200
300
400
500

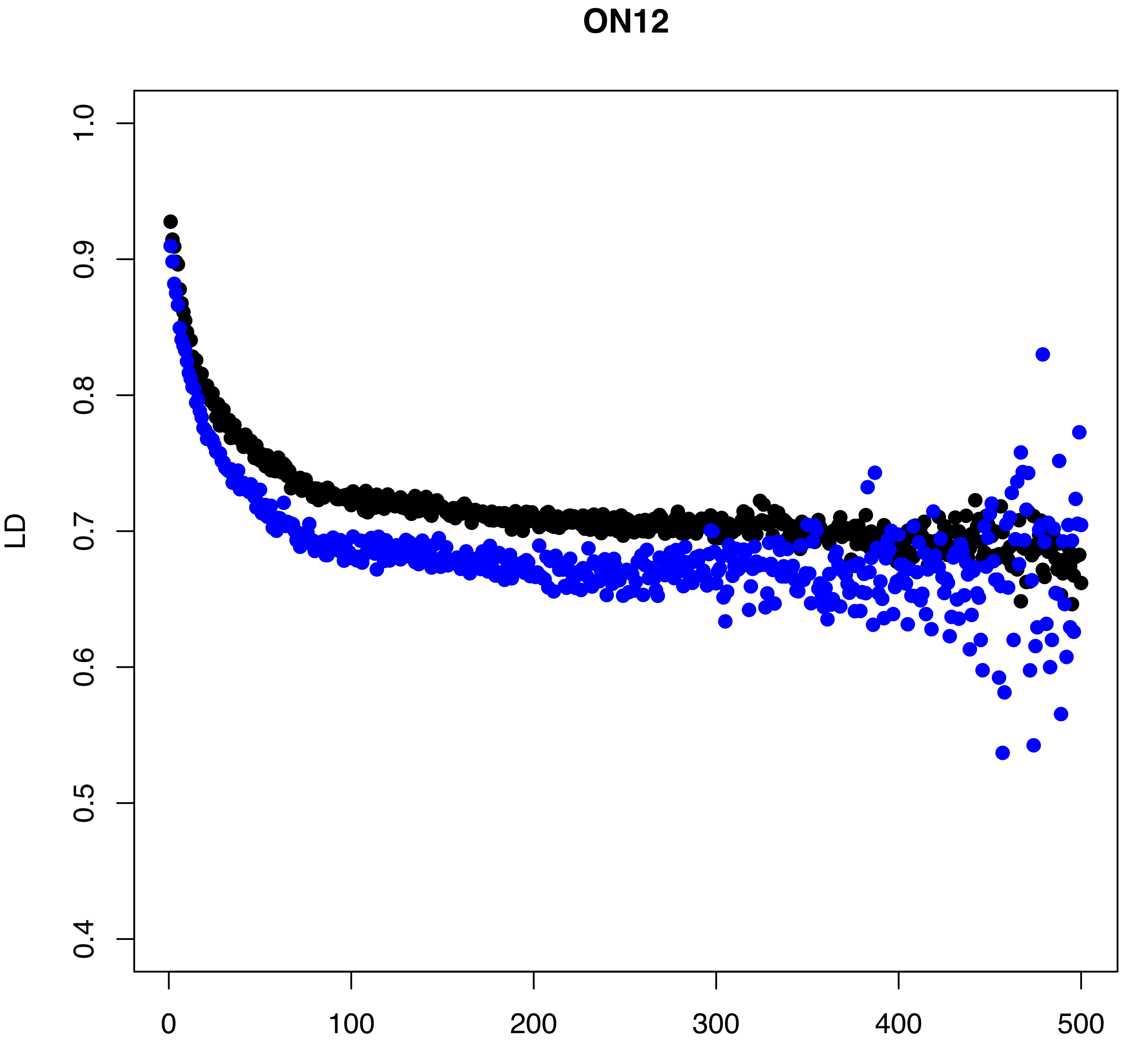

ON12
LD
1.0
0.9
0.8
0.7
0.6
0.5
0.4
0
100
200
300
400
500

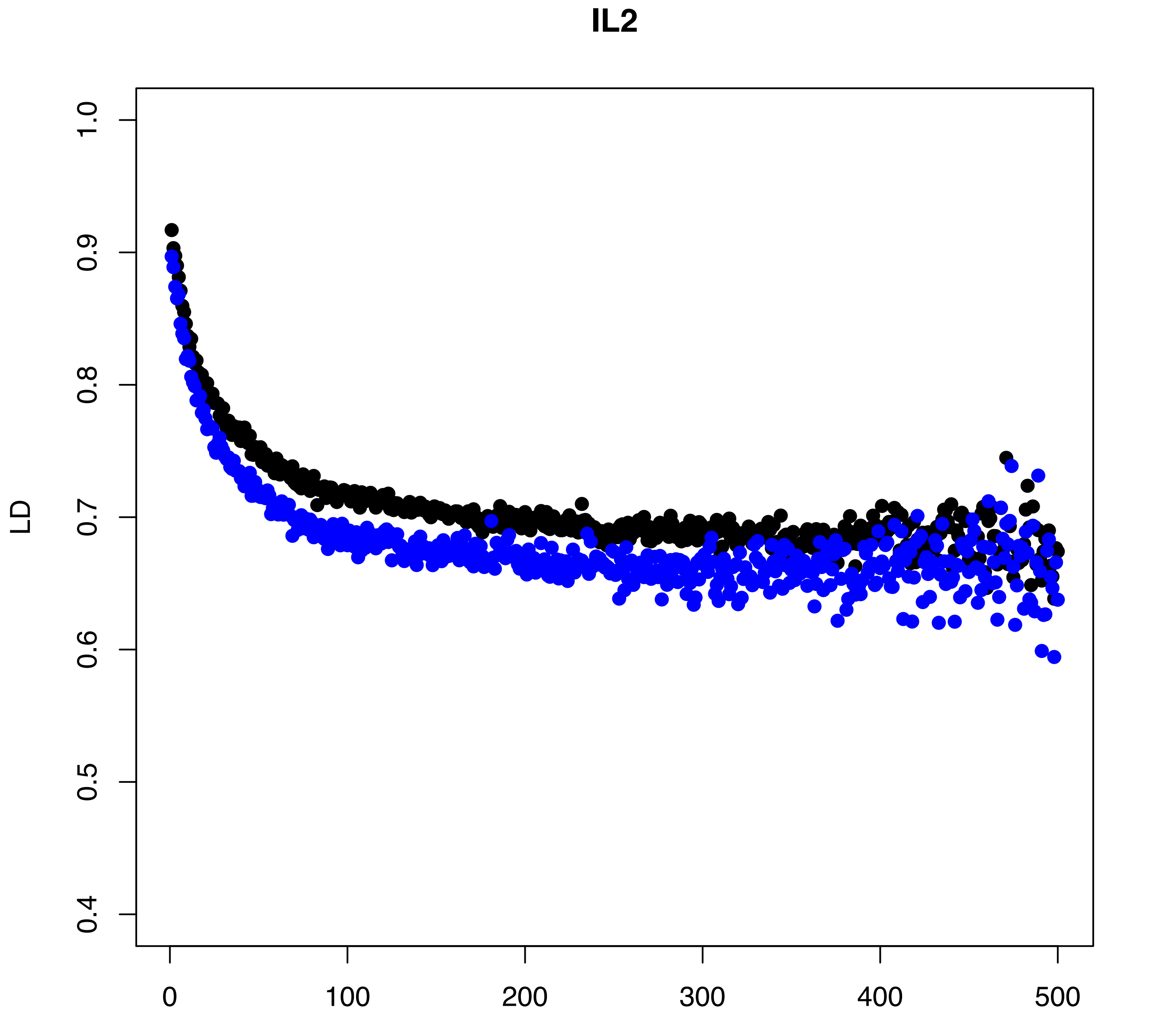

IL2
LD
1.0
0.9
0.8
0.7
0.6
0.5
0.4
0
100
200
300
400
500

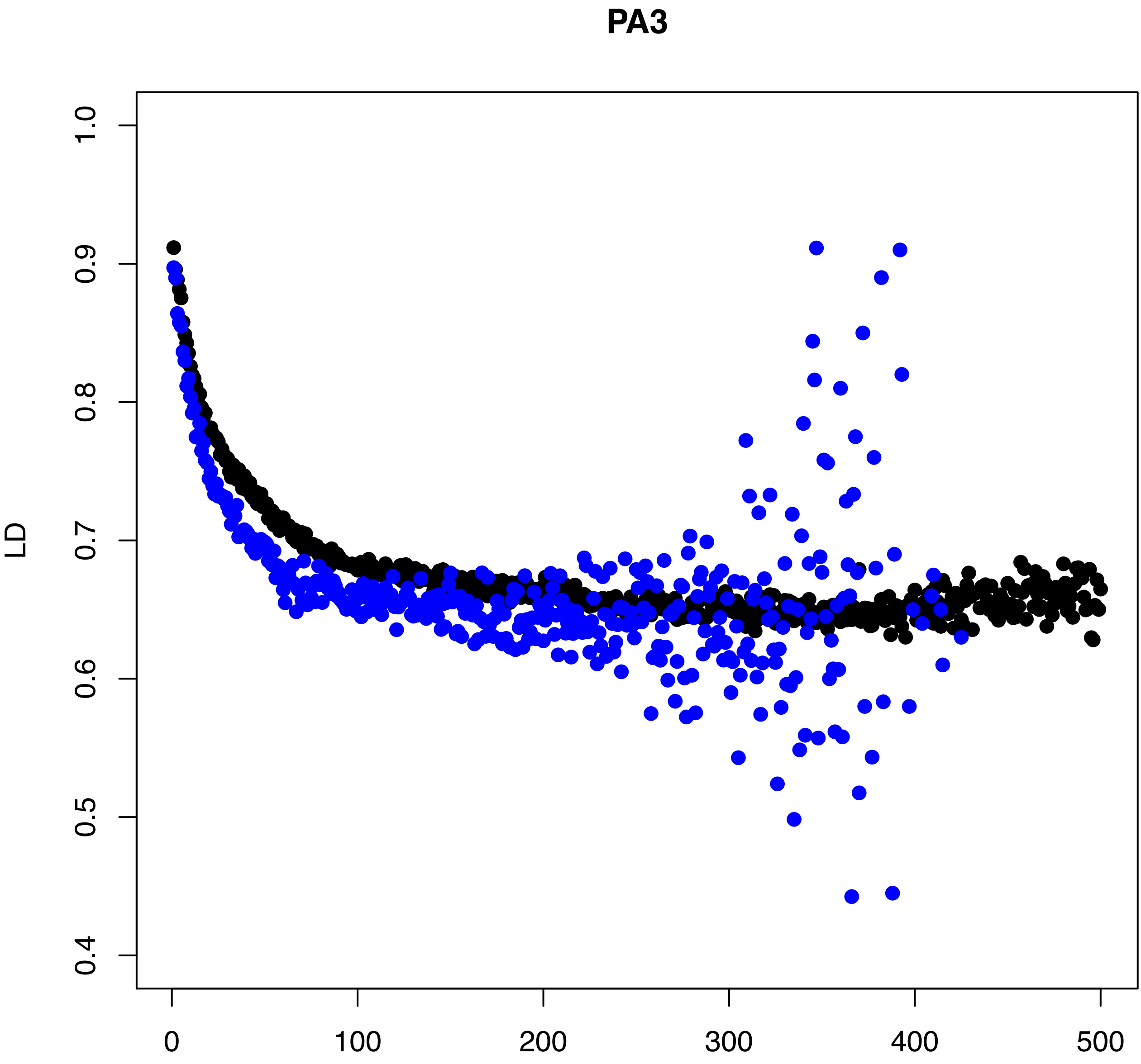
PA3
LD
1.0
0.9
0.8
0.7
0.6
0.5
0.4
0
100
200
300
400
500

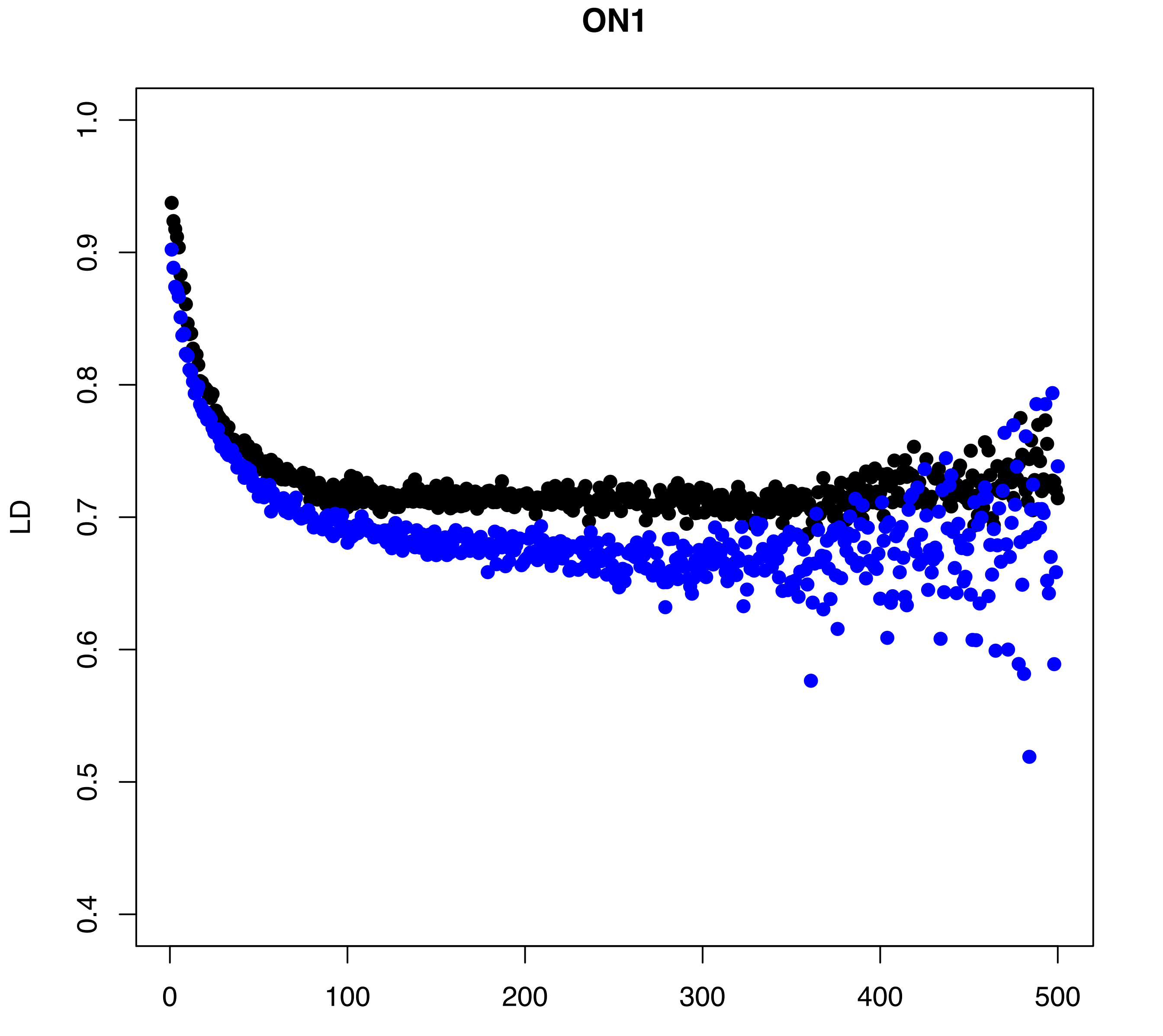
ON1
LD
1.0
0.9
0.8
0.7
0.6
0.5
0.4
0
100
200
300
400
500

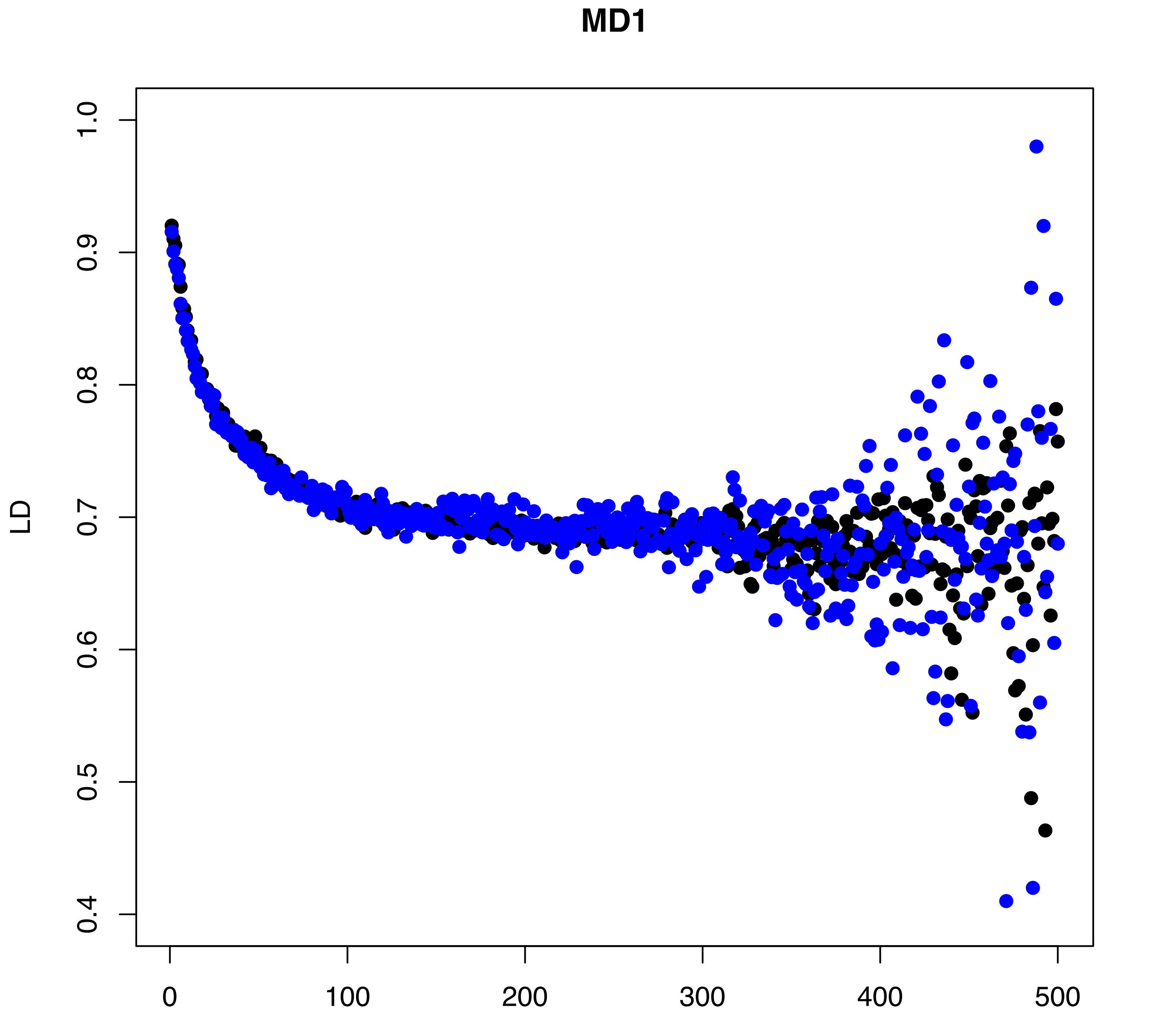
MD1
LD
1.0
0.9
0.8
0.7
0.6
0.5
0.4
0
100
200
300
400
500

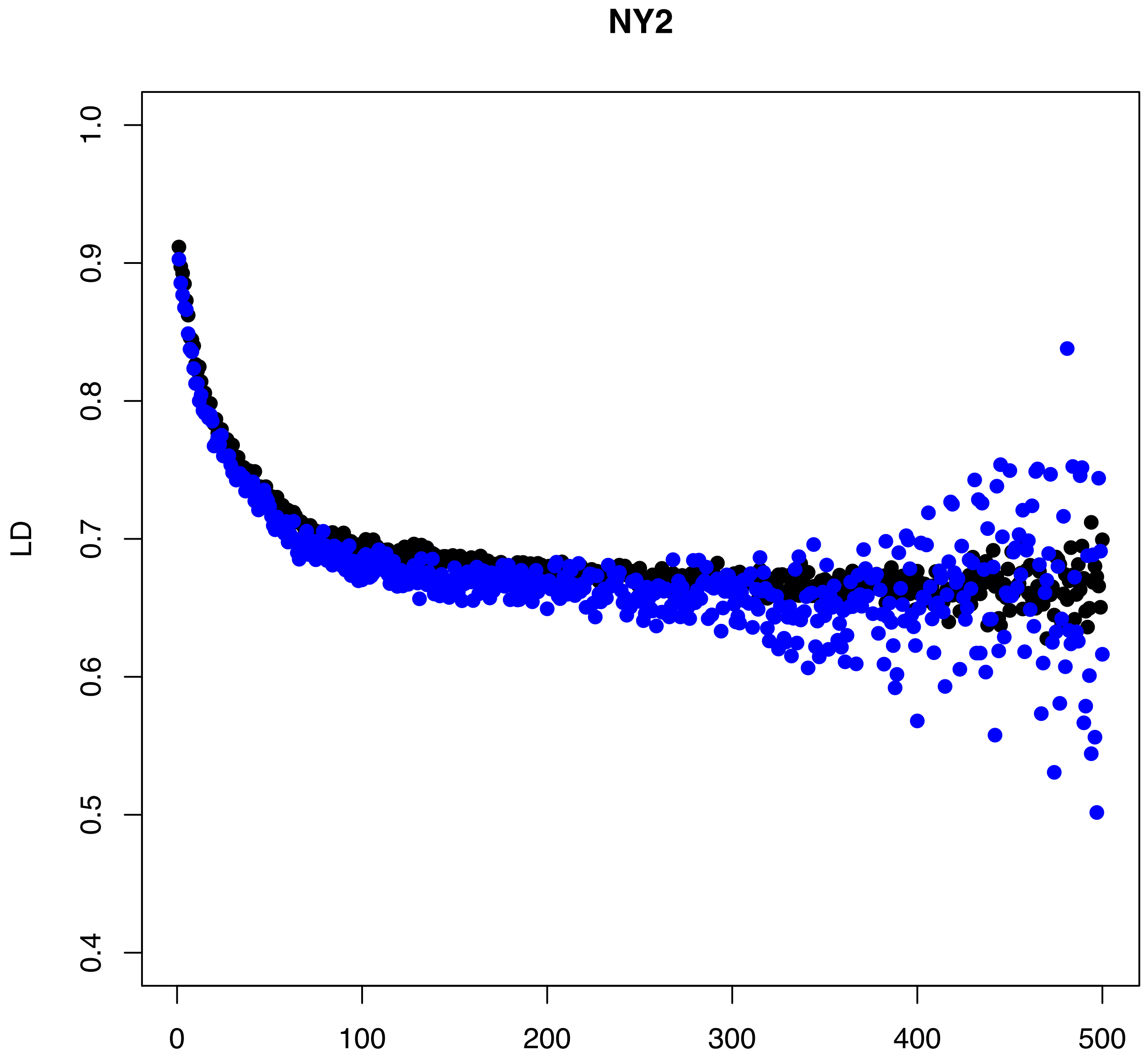
NY2
LD
1.0
0.9
0.8
0.7
0.6
0.5
0.4
0
100
200
300
400
500

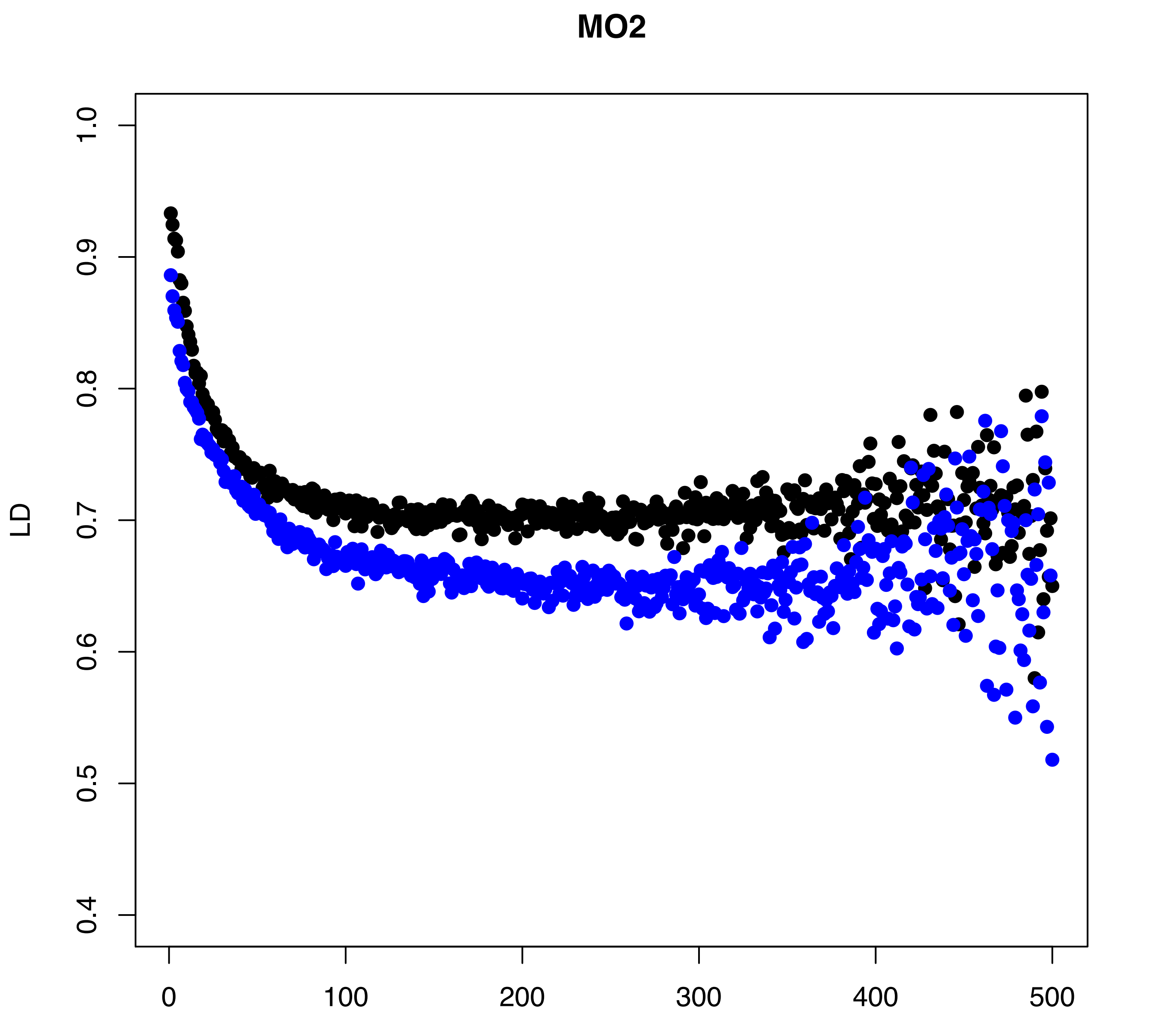
MO2
LD
1.0
0.9
0.8
0.7
0.6
0.5
0.4
0
100
200
300
400
500

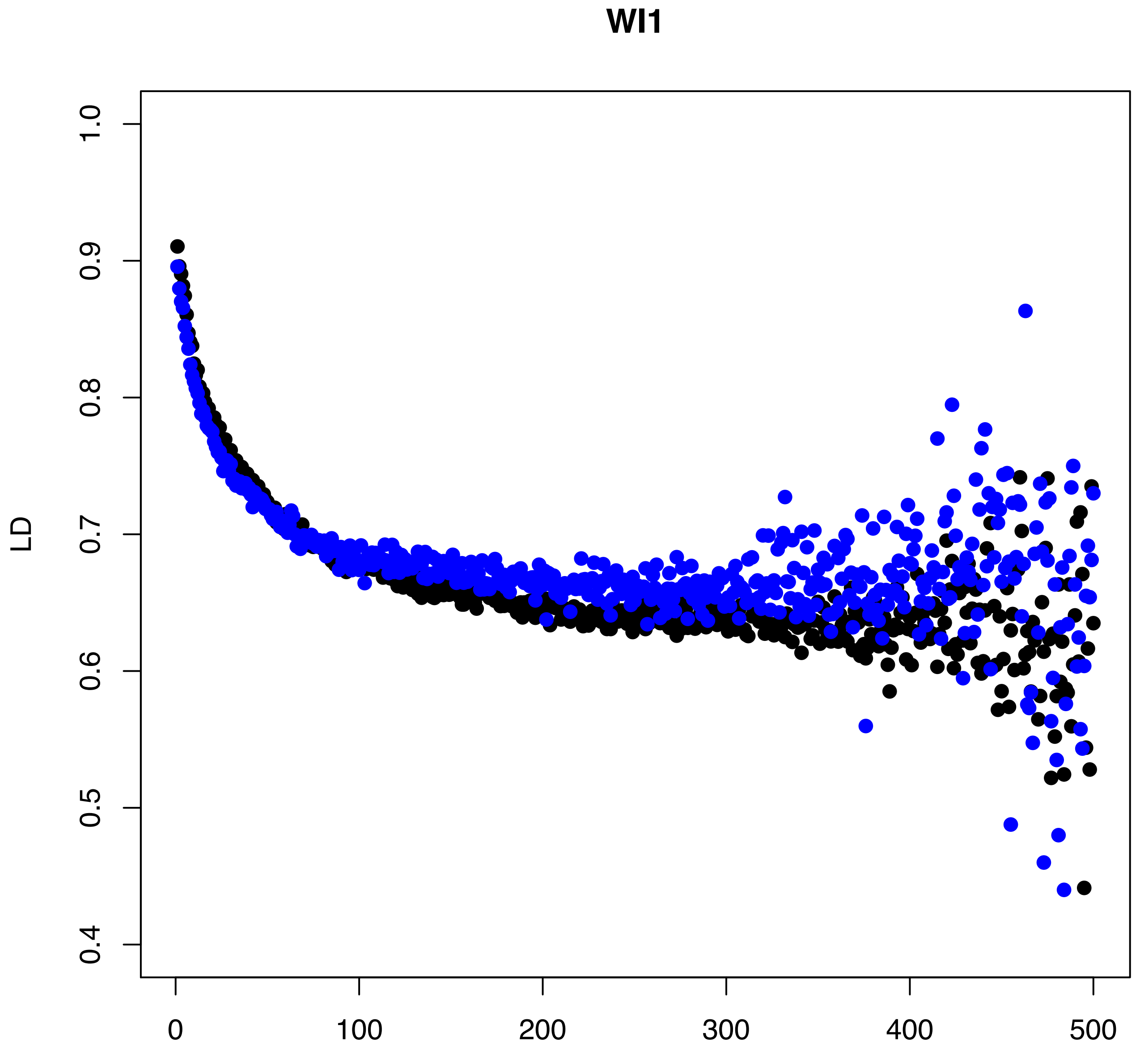
WI1
LD
1.0
0.9
0.8
0.7
0.6
0.5
0.4
0
100
200
300
400
500

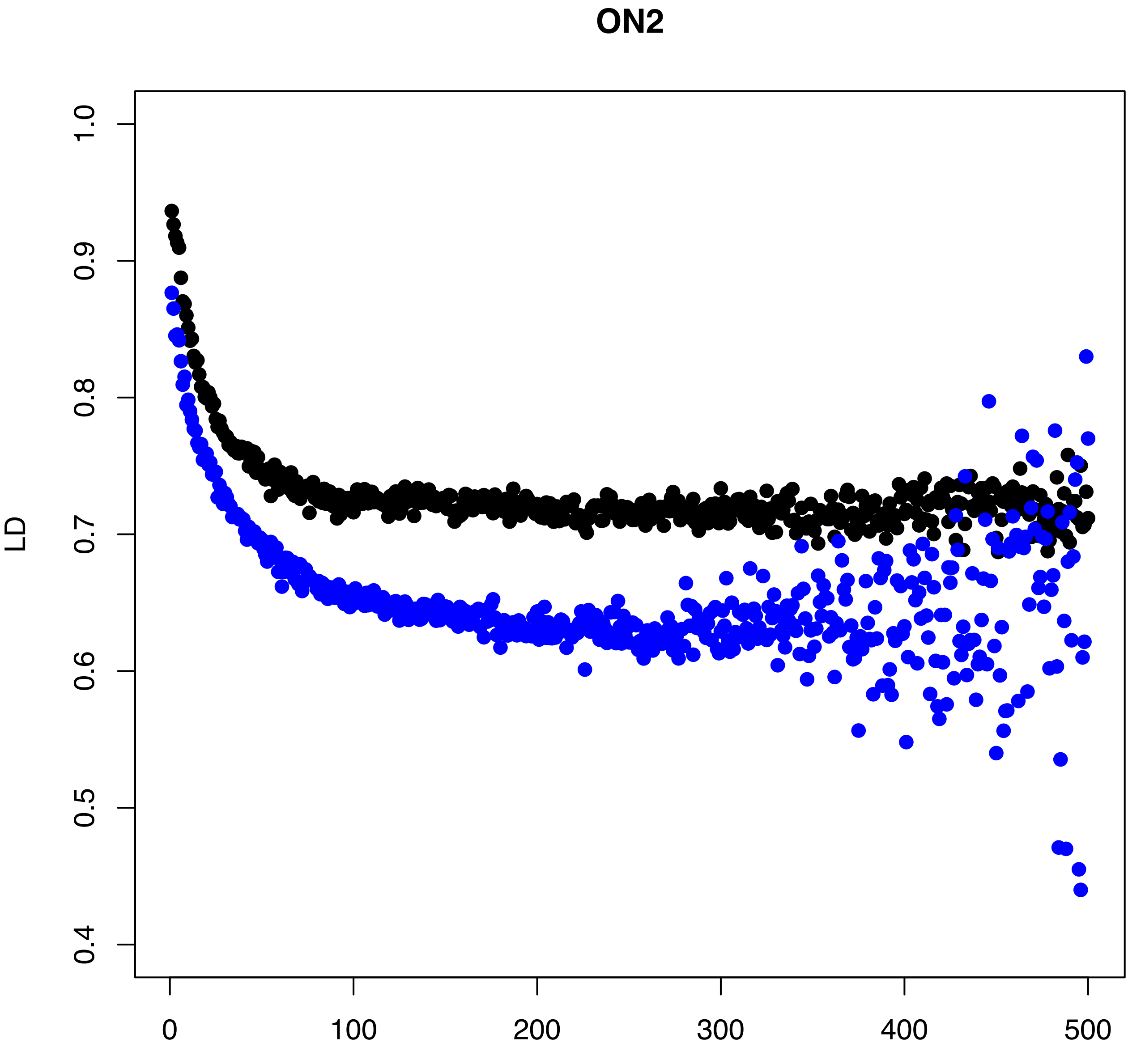
ON2
LD
1.0
0.9
0.8
0.7
0.6
0.5
0.4
0
100
200
300
400
500

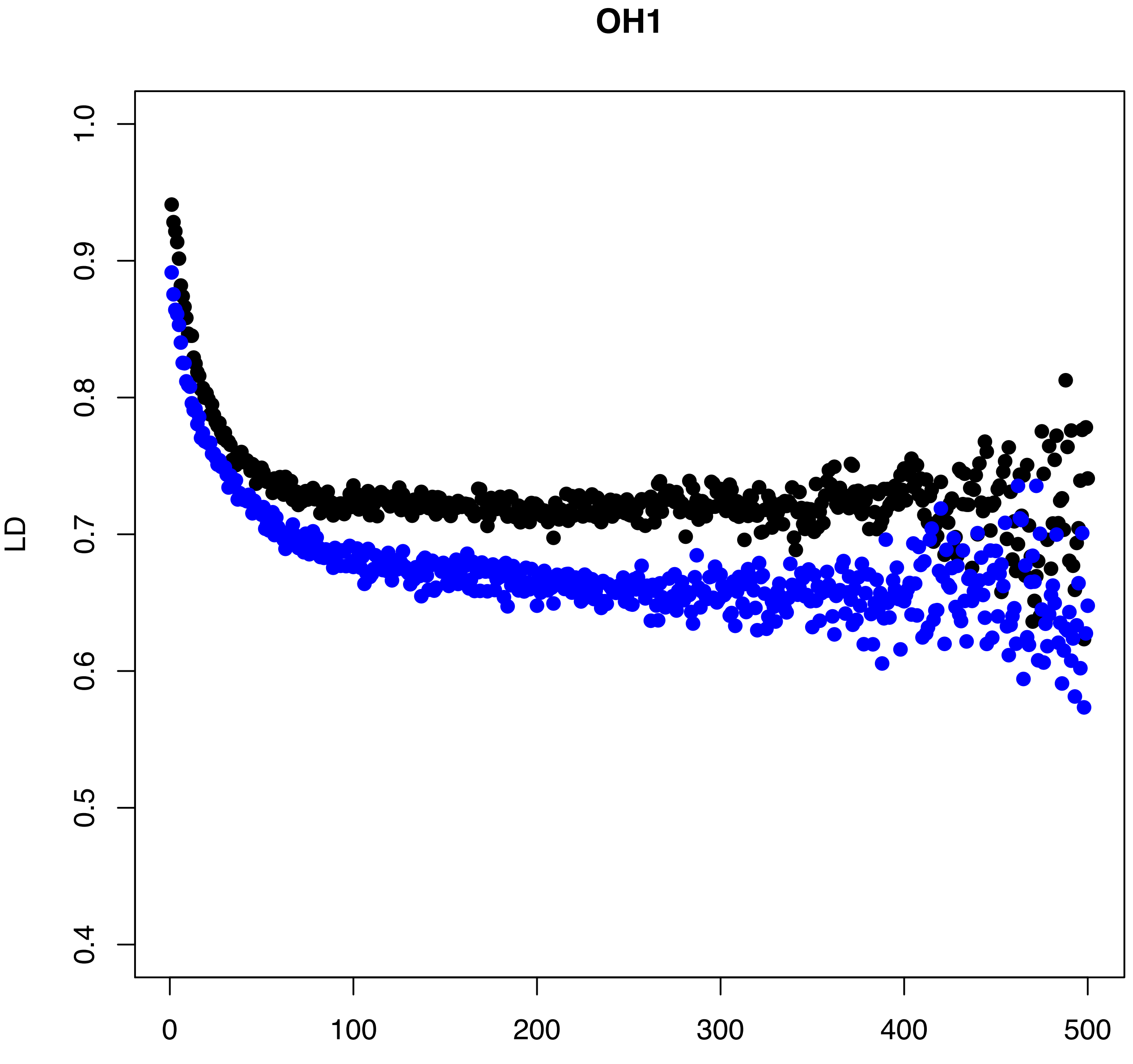

OH1
LD
1.0
0.9
0.8
0.7
0.6
0.5
0.4
0
100
200
300
400
500

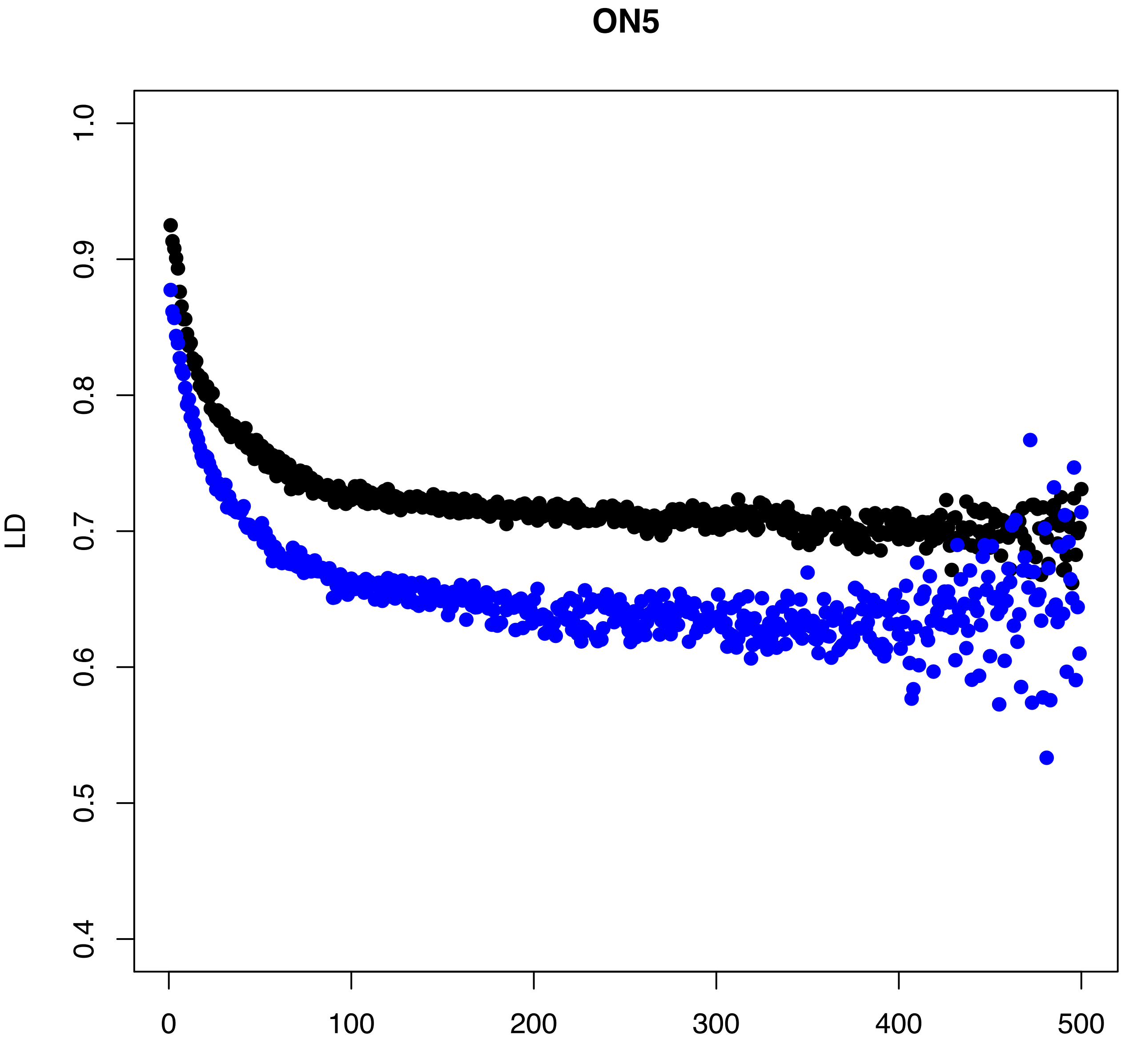

ON5
LD
1.0
0.9
0.8
0.7
0.6
0.5
0.4
0
100
200
300
400
500

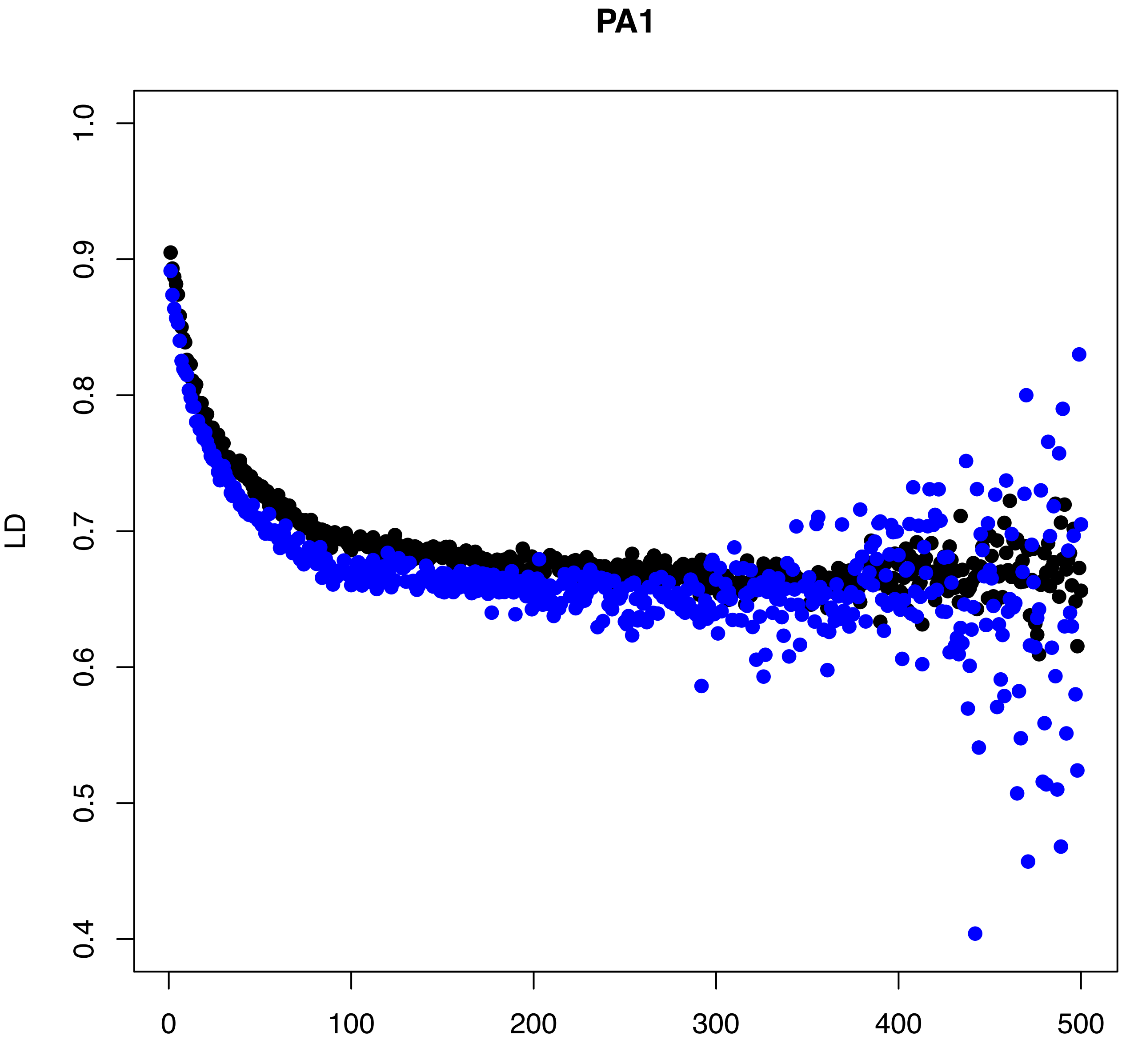

PA1
LD
1.0
0.9
0.8
0.7
0.6
0.5
0.4
0
100
200
300
400
500

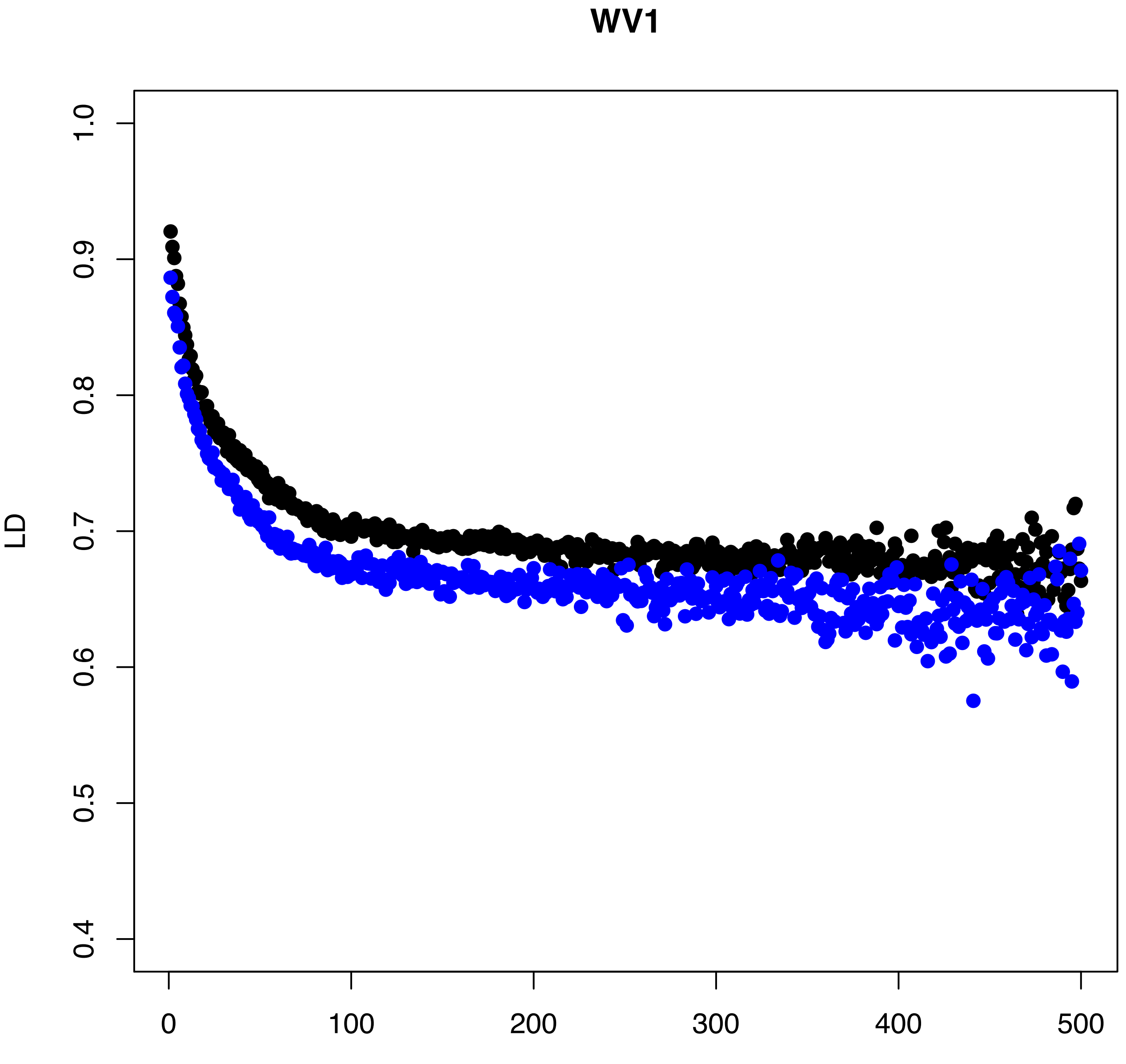

WV1
LD
1.0
0.9
0.8
0.7
0.6
0.5
0.4
0
100
200
300
400
500

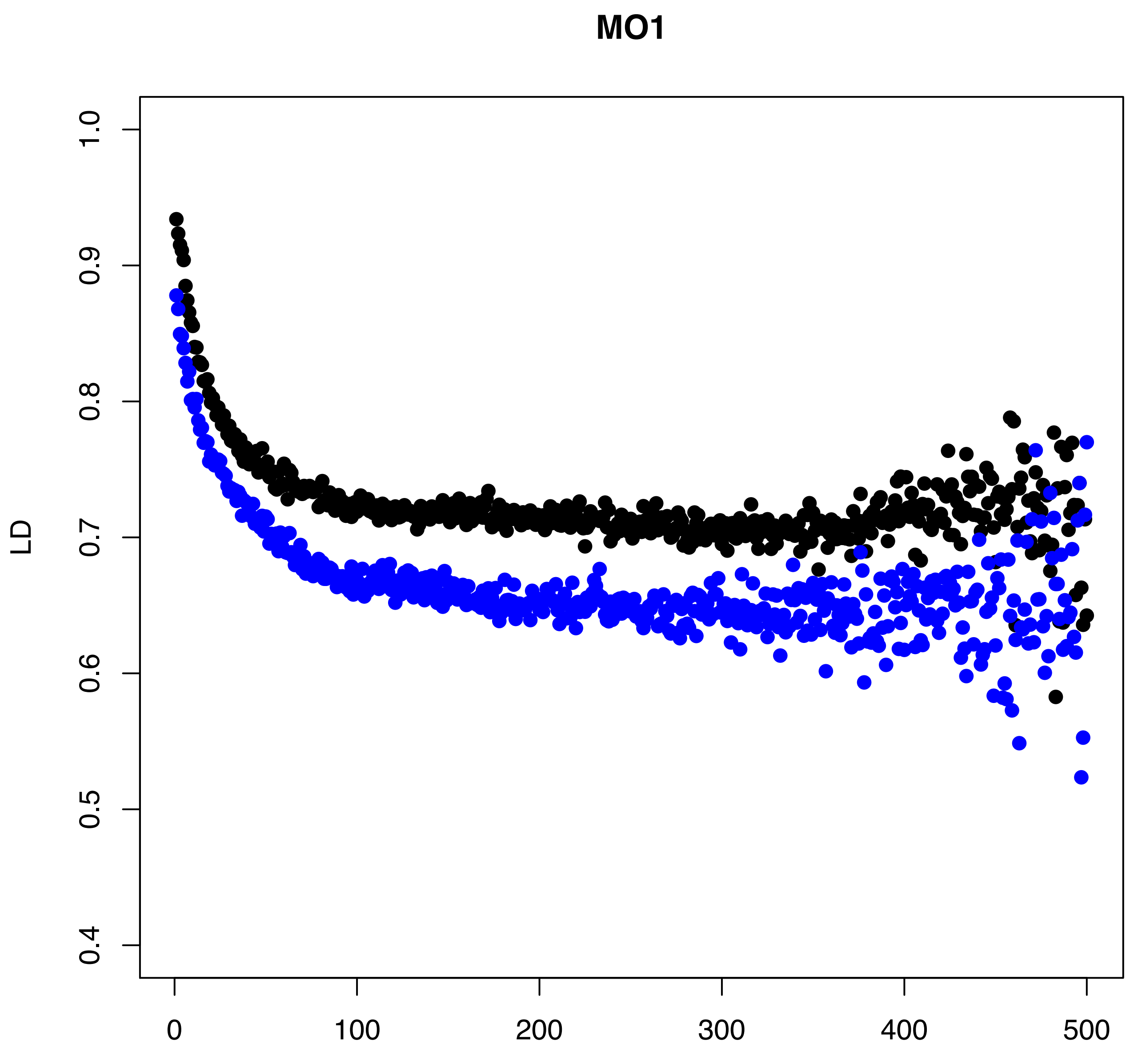
MO1
LD
1.0
0.9
0.8
0.7
0.6
0.5
0.4
0
100
200
300
400
500

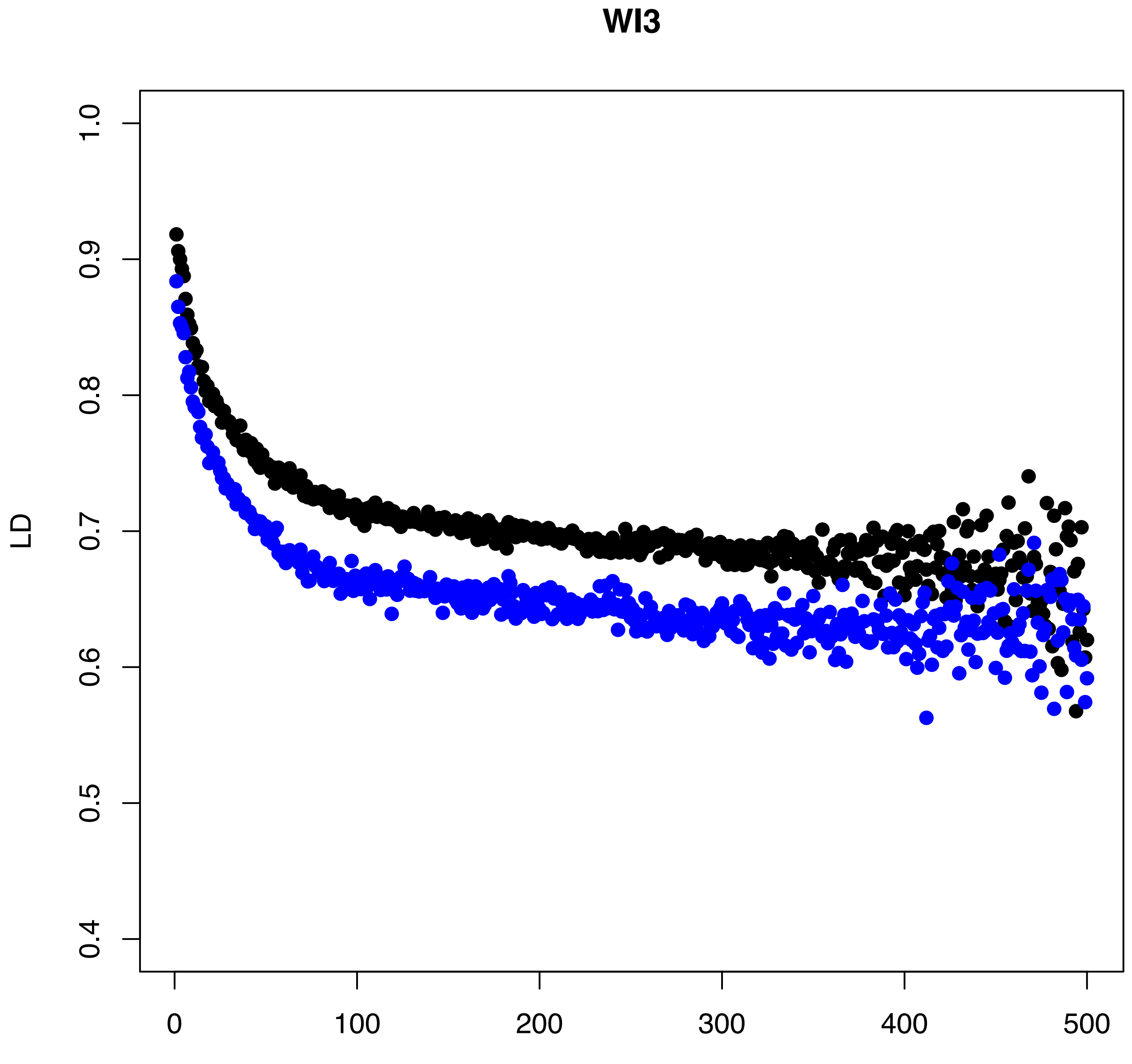
WI3
LD
1.0
0.9
0.8
0.7
0.6
0.5
0.4
0
100
200
300
400
500

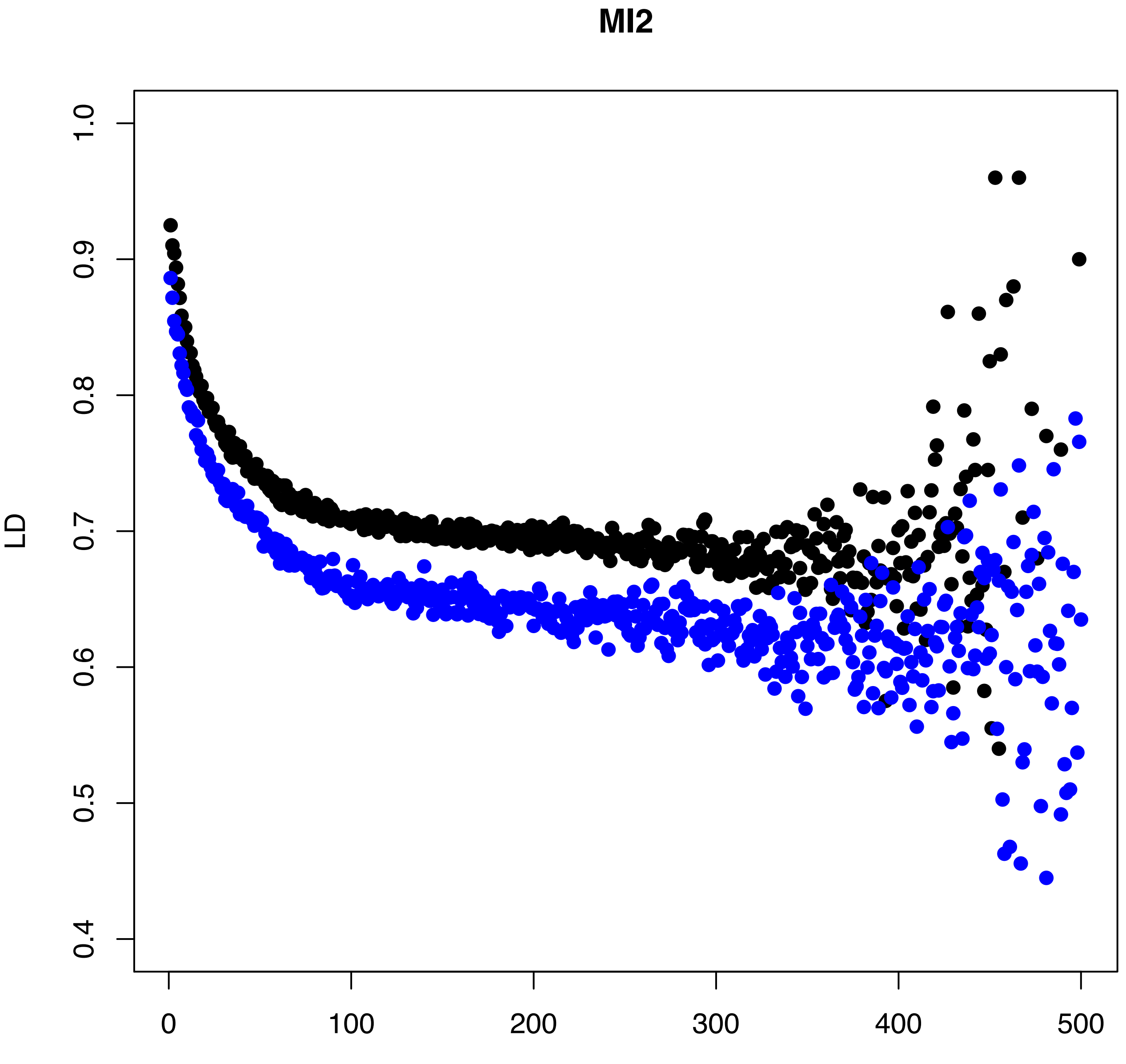
MI2
LD
1.0
0.9
0.8
0.7
0.6
0.5
0.4
0
100
200
300
400
500

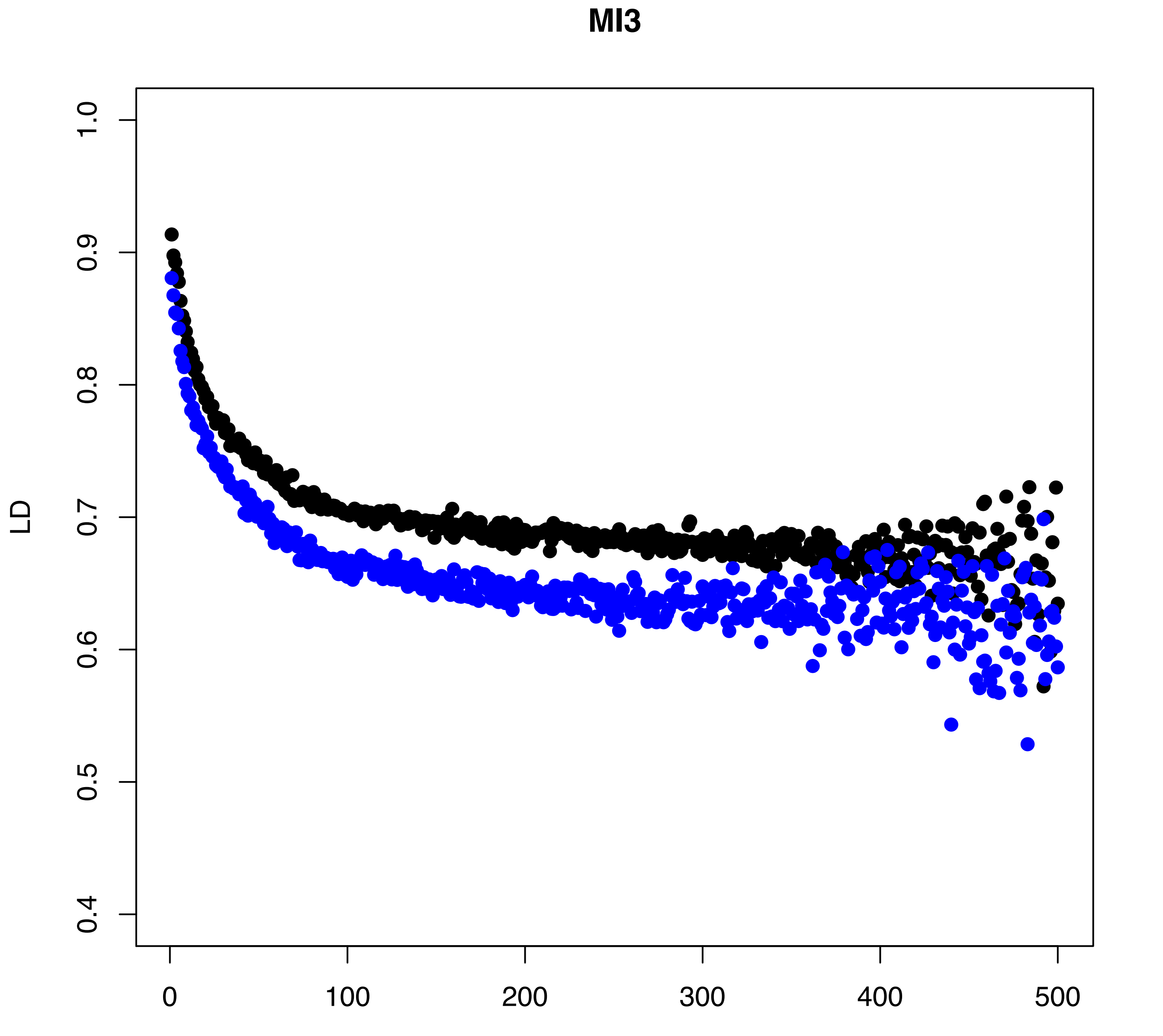
MI3
LD
1.0
0.9
0.8
0.7
0.6
0.5
0.4
0
100
200
300
400
500

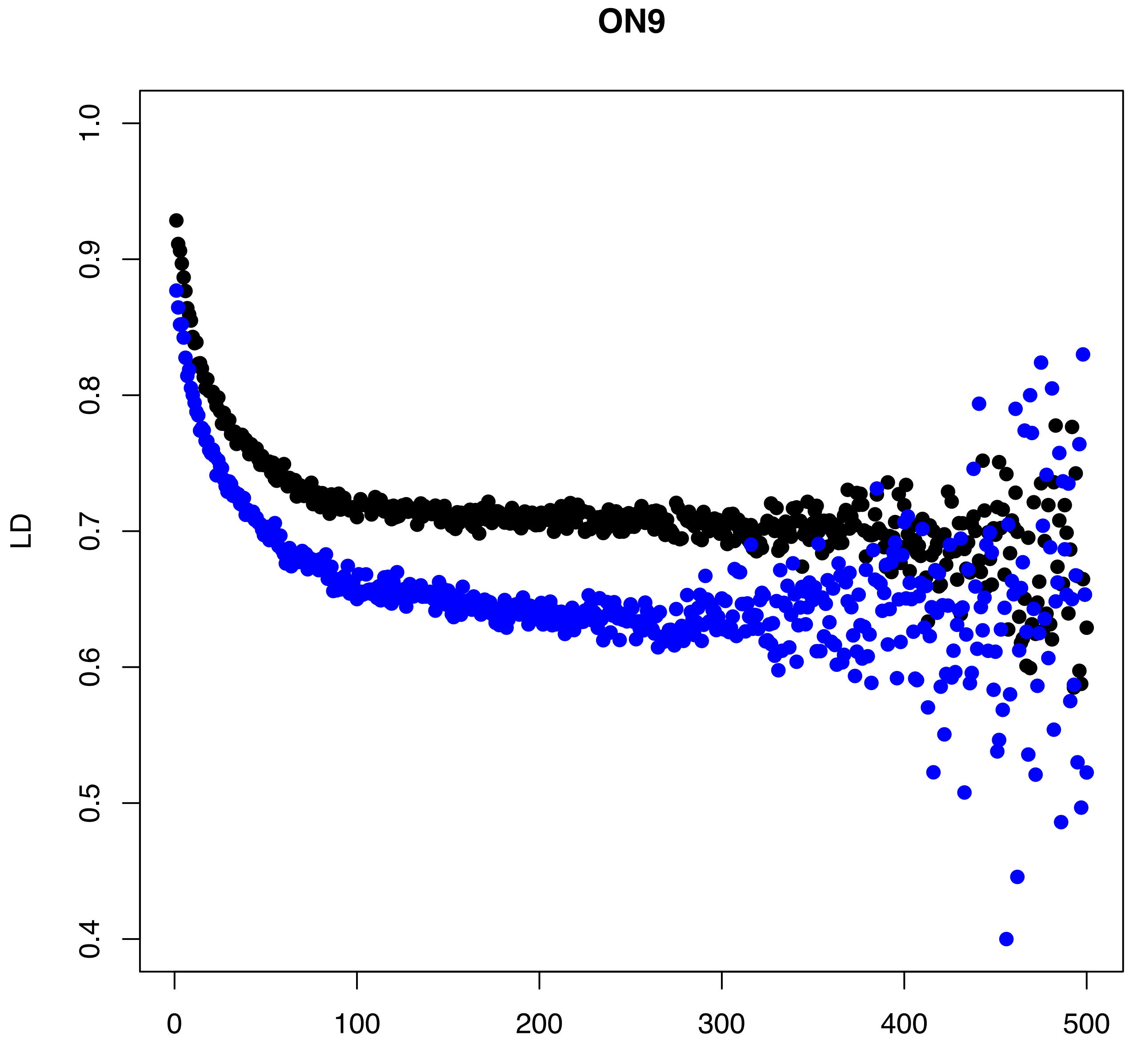
ON9
LD
1.0
0.9
0.8
0.7
0.6
0.5
0.4
0
100
200
300
400
500

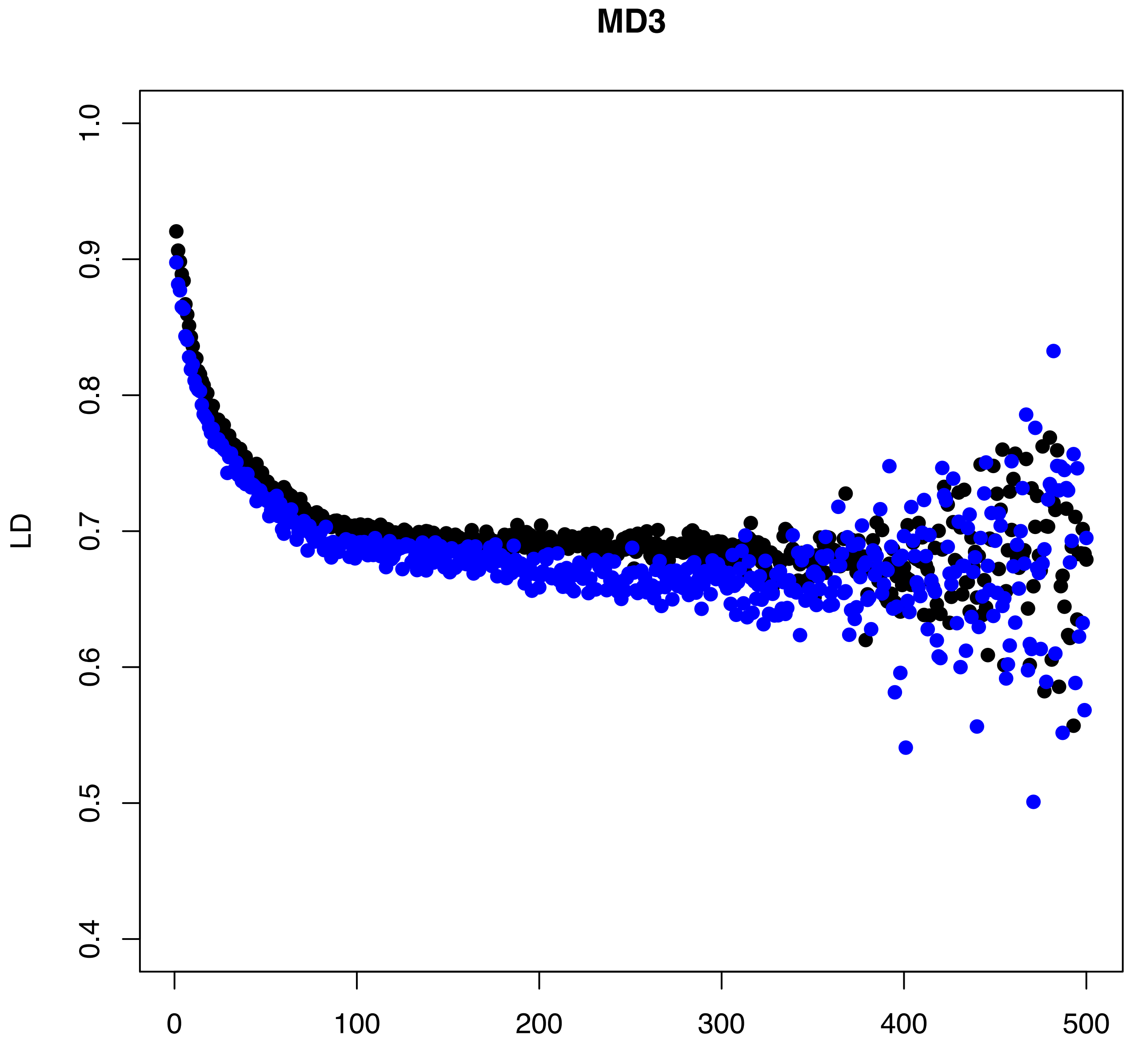
MD3
LD
1.0
0.9
0.8
0.7
0.6
0.5
0.4
0
100
200
300
400
500

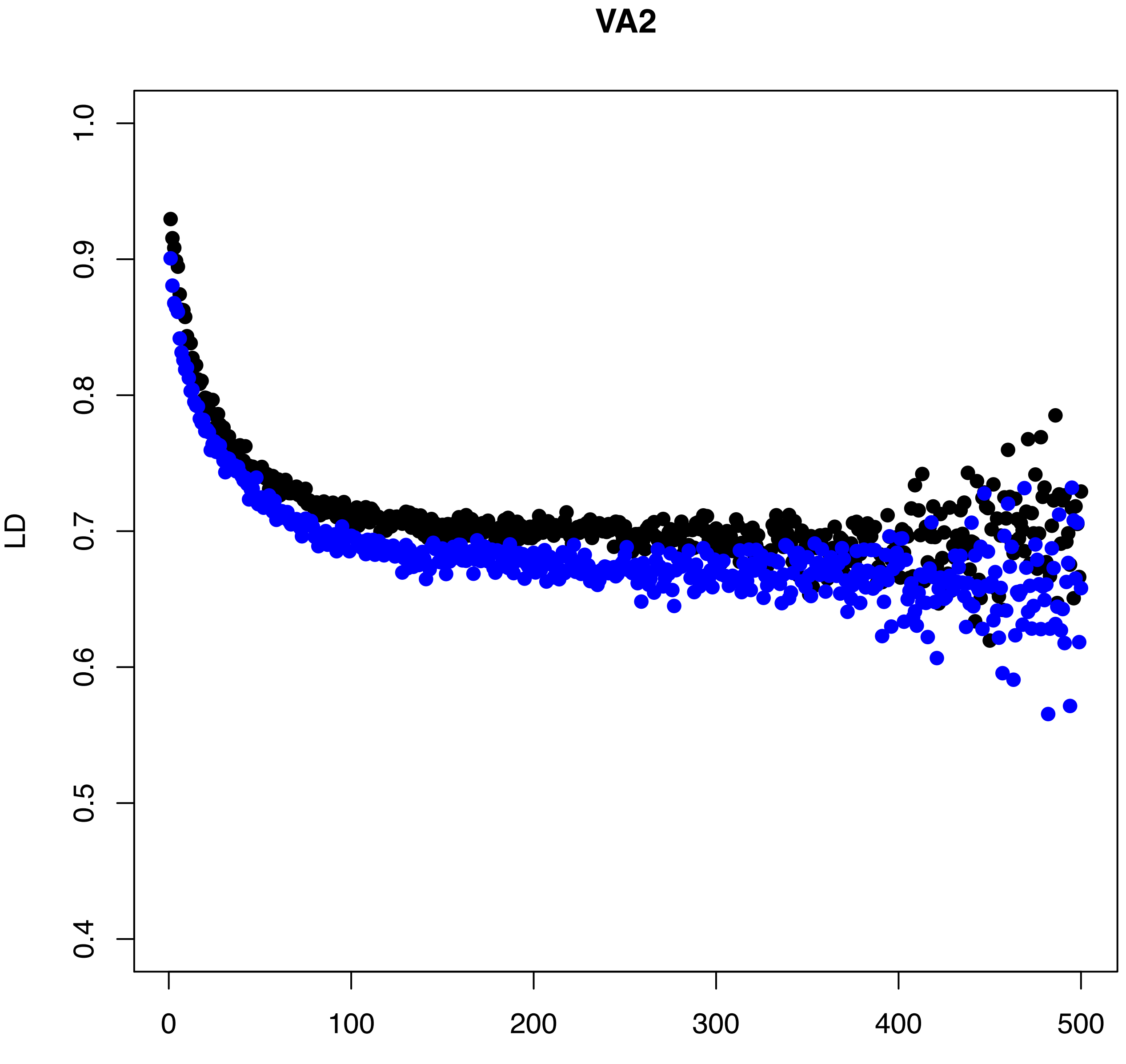

VA2
LD
1.0
0.9
0.8
0.7
0.6
0.5
0.4
0
100
200
300
400
500

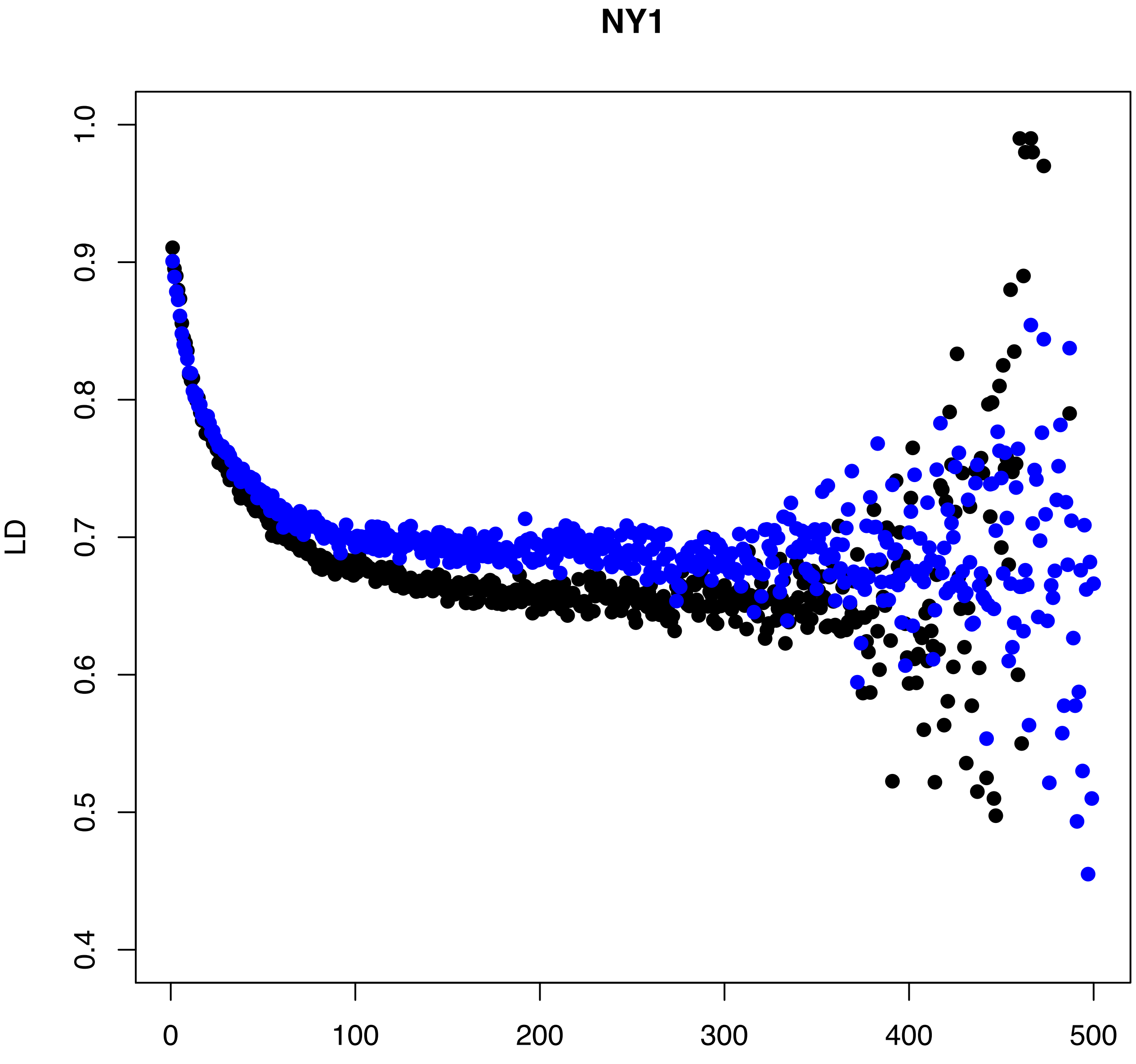
NY1
LD
1.0
0.9
0.8
0.7
0.6
0.5
0.4
0
100
200
300
400
500

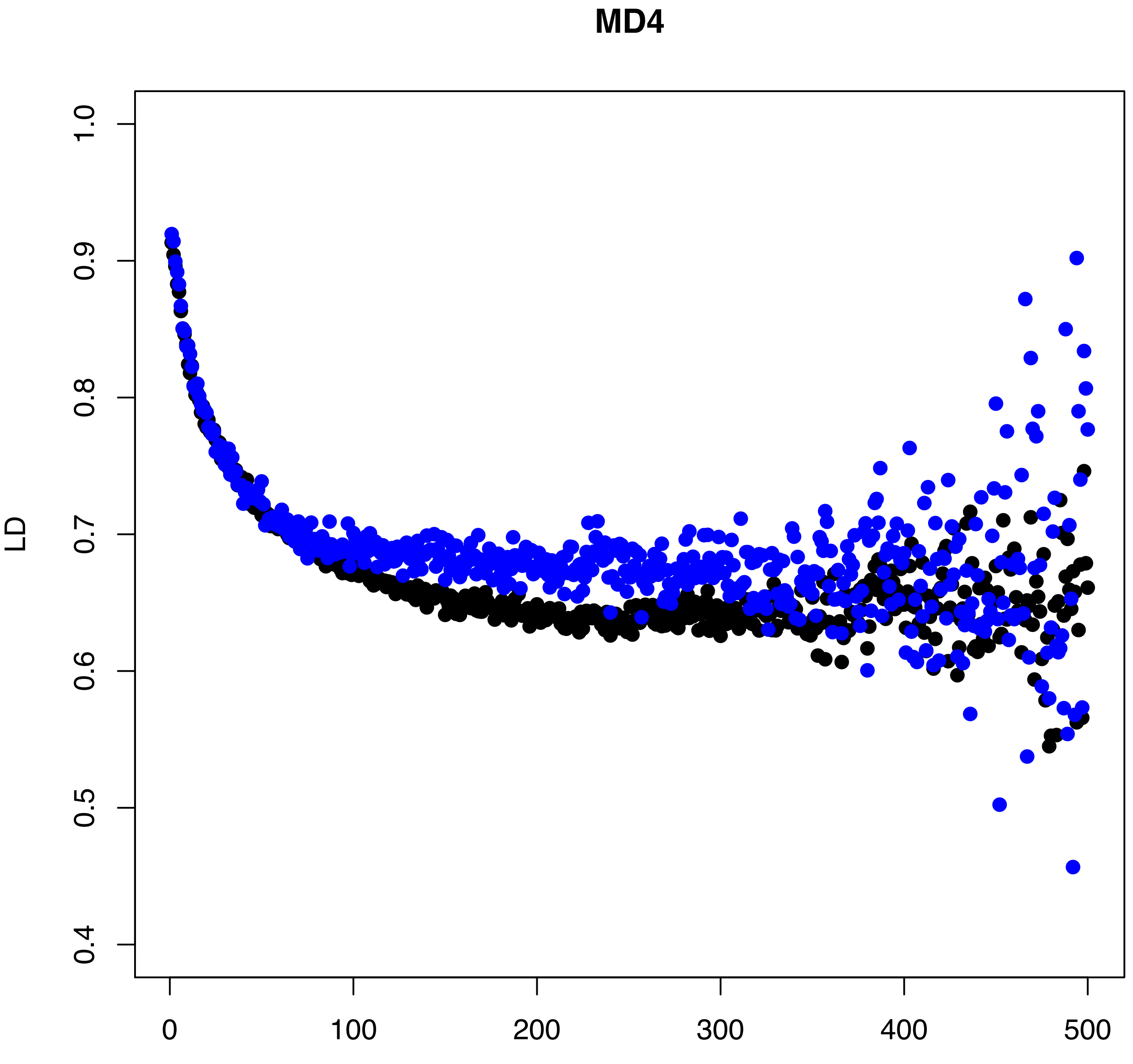

MD4
LD
1.0
0.9
0.8
0.7
0.6
0.5
0.4
0
100
200
300
400
500

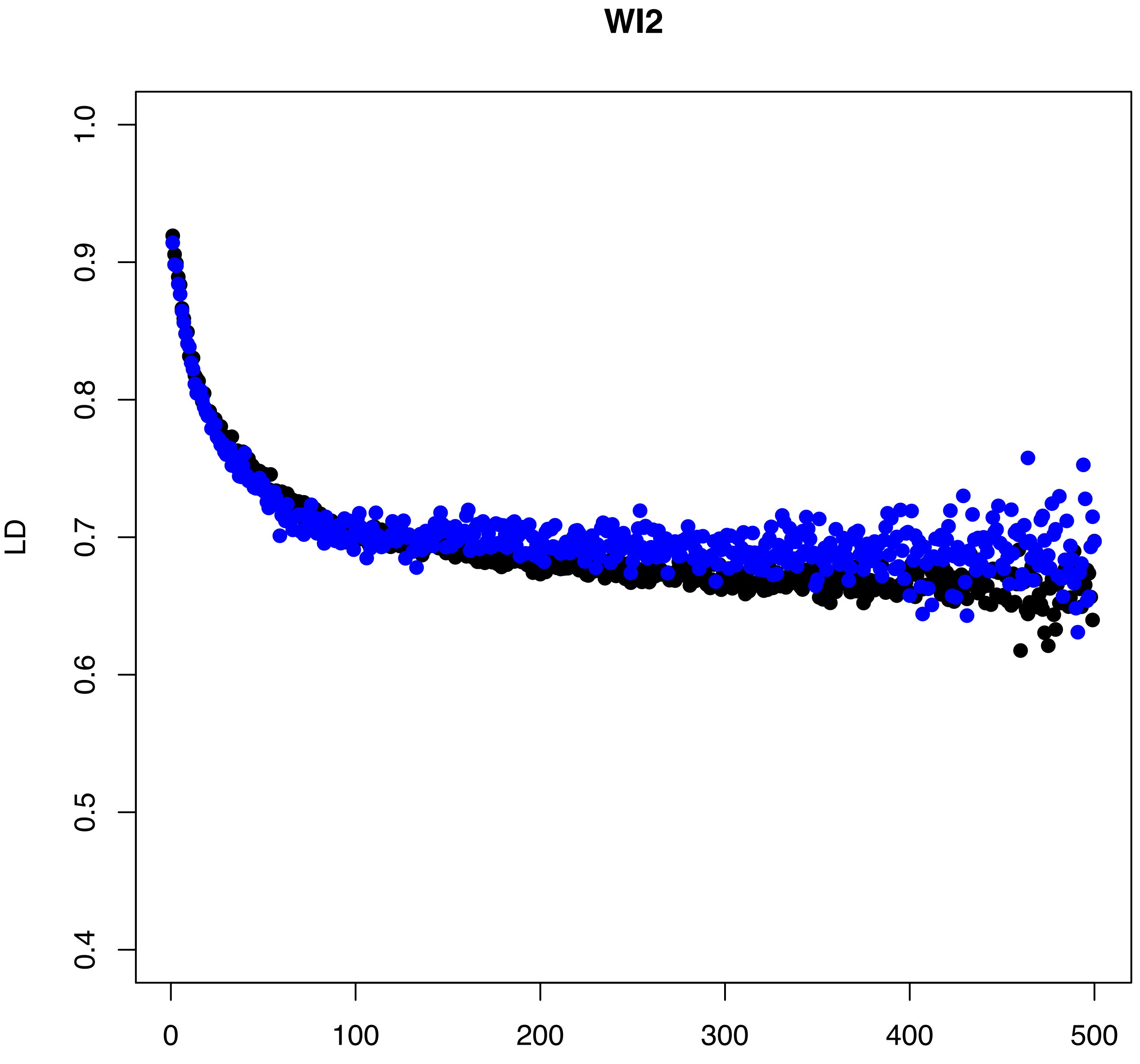
WI2
LD
1.0
0.9
0.8
0.7
0.6
0.5
0.4
0
100
200
300
400
500

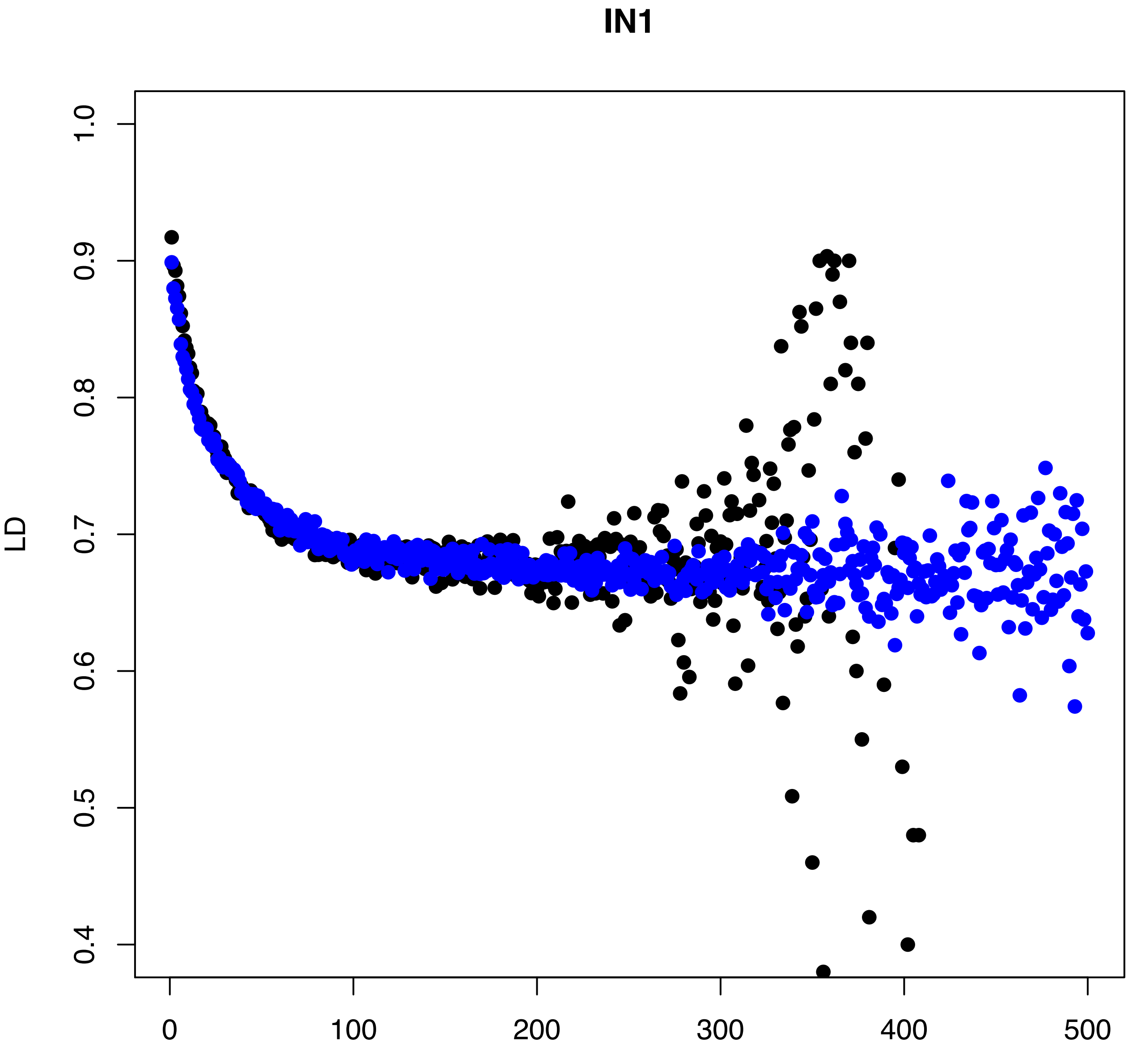

IN1
LD
1.0
0.9
0.8
0.7
0.6
0.5
0.4
0
100
200
300
400
500

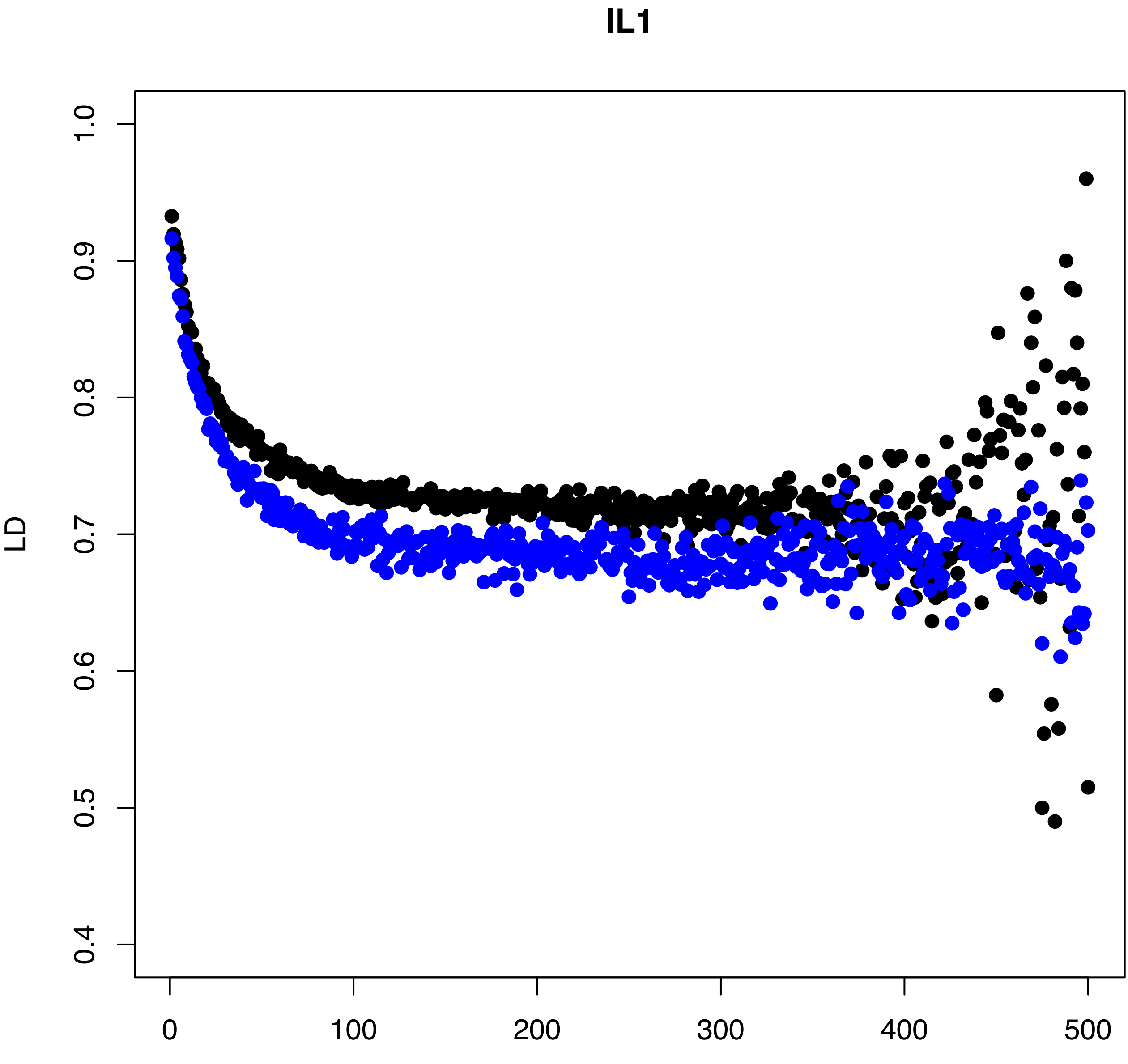

IL1
LD
1.0
0.9
0.8
0.7
0.6
0.5
0.4
0
100
200
300
400
500

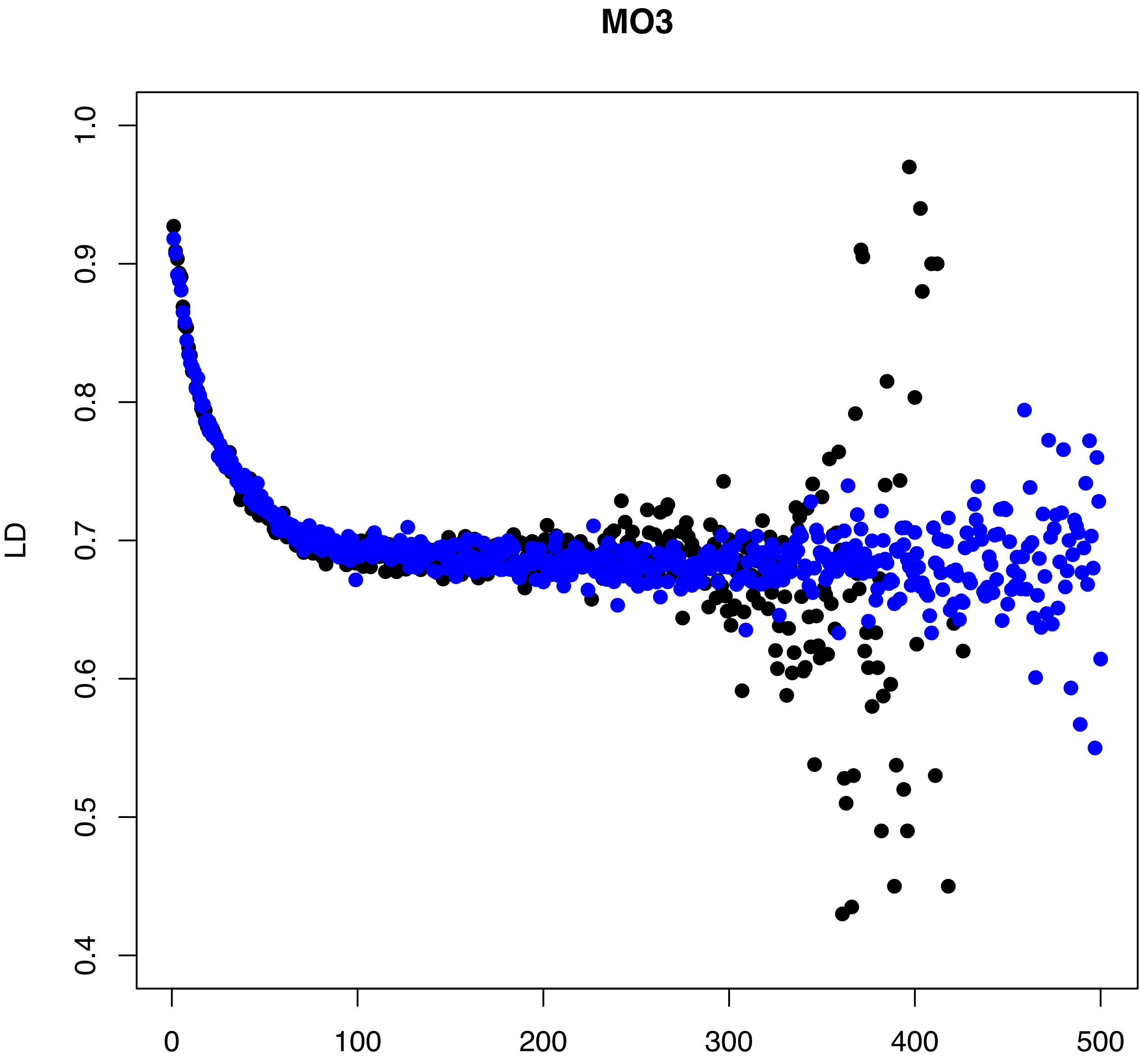
MO3
LD
1.0
0.9
0.8
0.7
0.6
0.5
0.4
0
100
200
300
400
500
