# Supplementary material for: Drivers of linkage disequilibrium across a species’ geographic range
Source: PLoS Genet. 2021 Mar 26;17(3):e1009477. doi: 10.1371/journal.pgen.1009477 (PMC8026057; doi:10.1371/journal.pgen.1009477)
Supplement: S2 Fig — Average LD, measured as r2 between SNPs from pool-seq data for each analyzed genic (black, grey) and intergenic (red, orange) region for each population across scaffolds I-VIII of the A. lyrata genome plotted for each population (S1 Table). (PDF) [file pgen.1009477.s002.pdf]

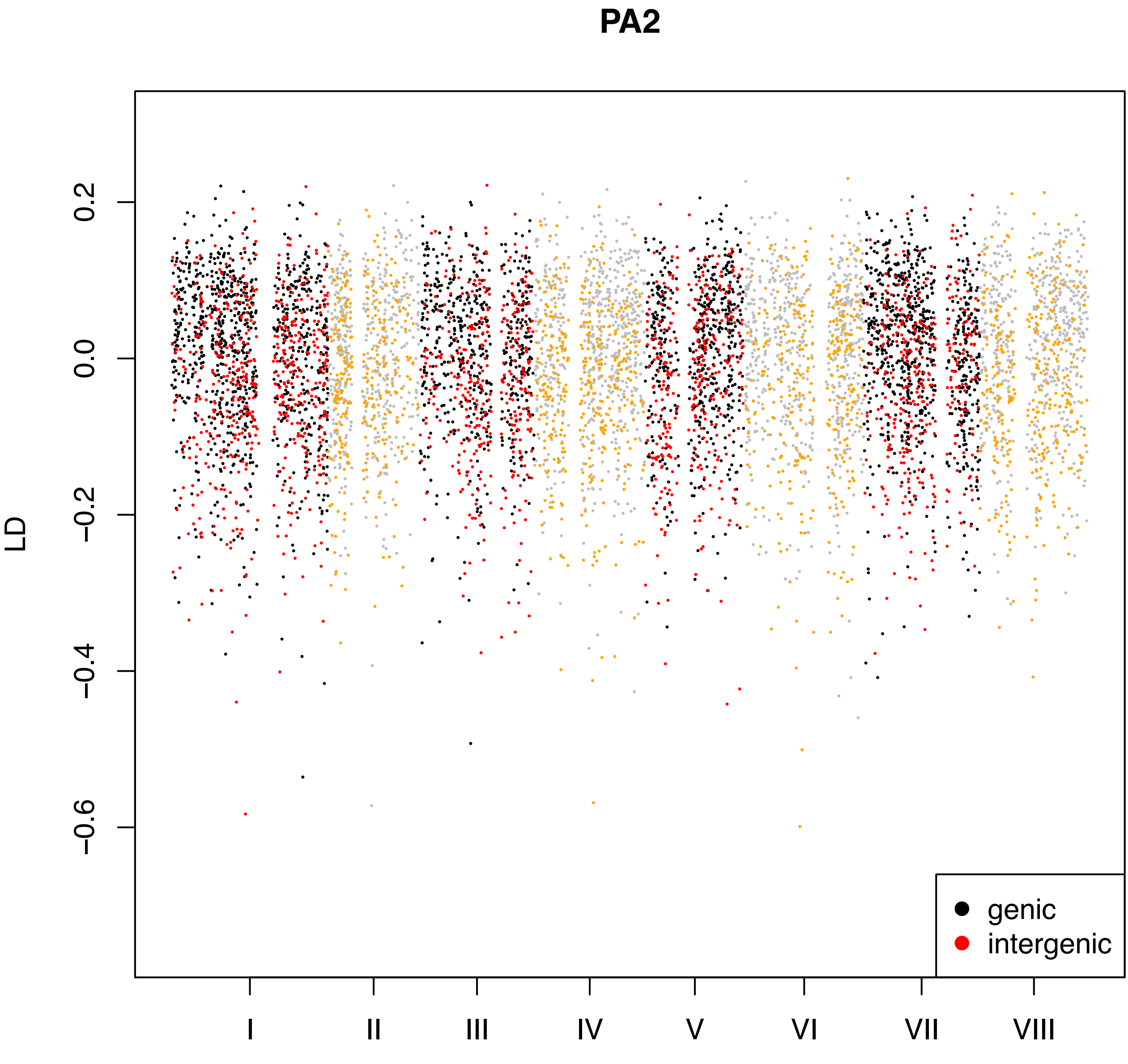
PA2
LD
0.2
0.0
−0.2
−0.4
−0.6
I
II
III
IV
V
VI
VII
VIII
genic
intergenic

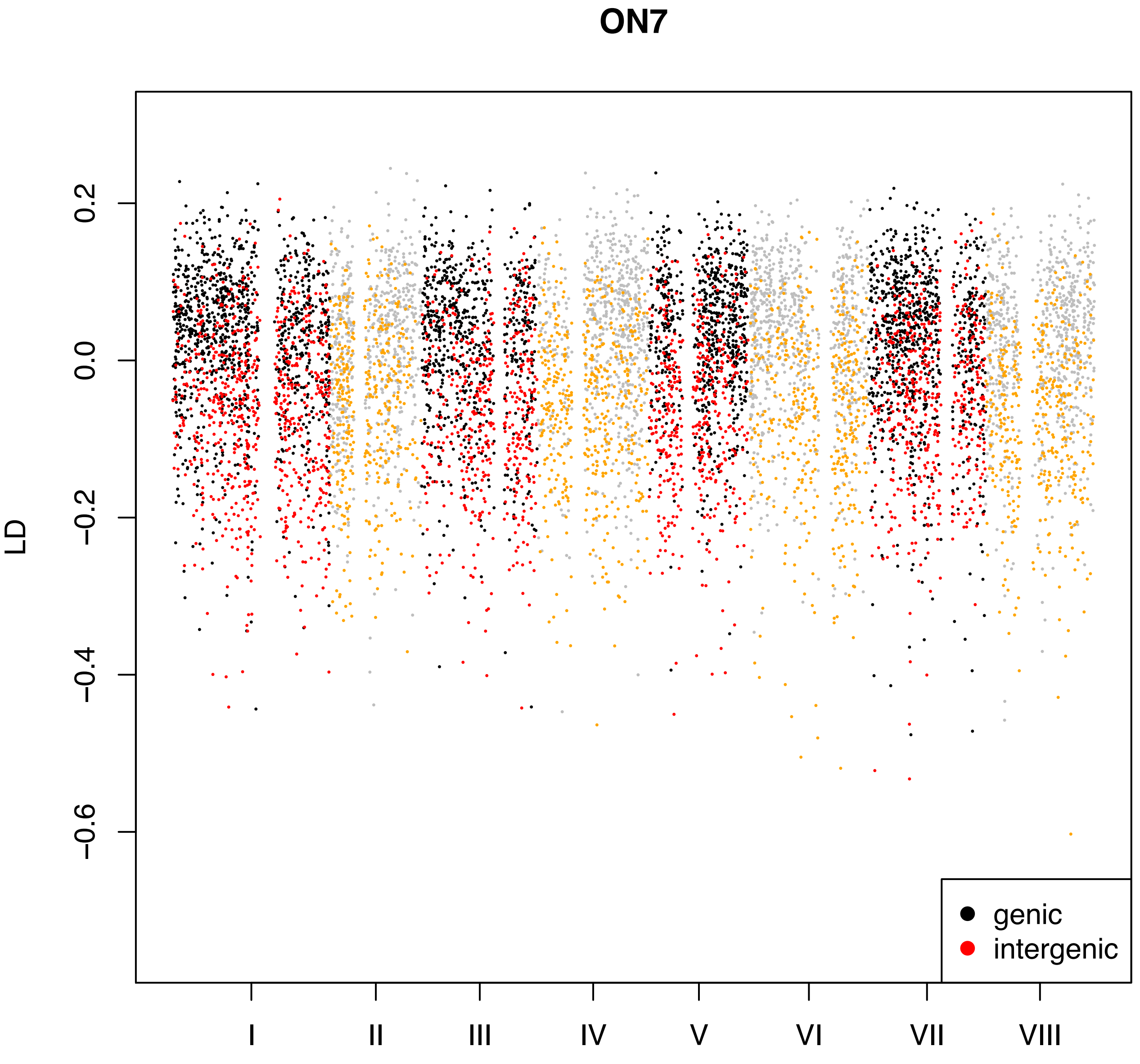
ON7
LD
0.2
0.0
−0.2
−0.4
−0.6
I
II
III
IV
V
VI
VII
VIII
genic
intergenic

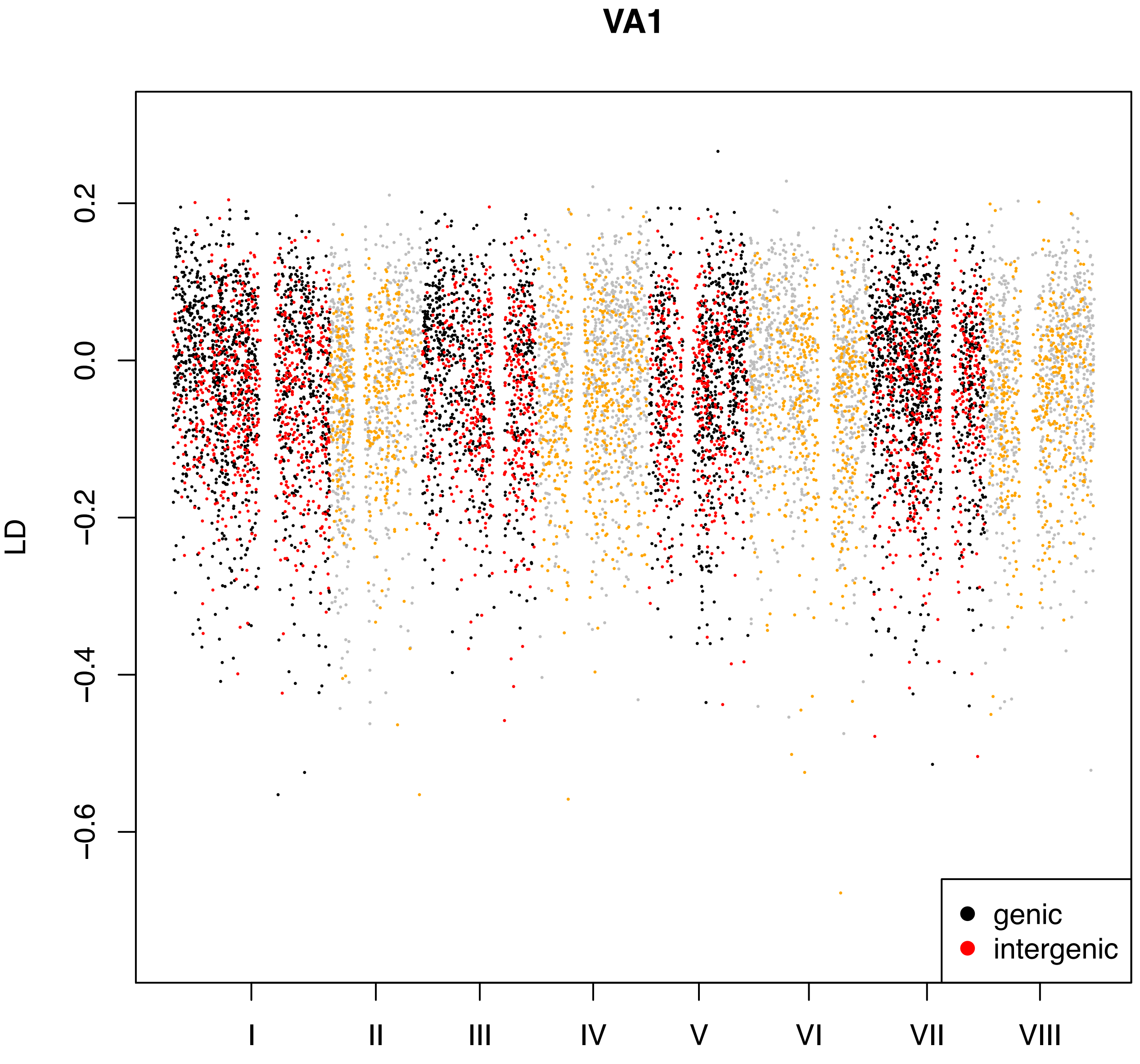

VA1
LD
0.2
0.0
−0.2
−0.4
−0.6
I
II
III
IV
V
VI
VII
VIII
genic
intergenic

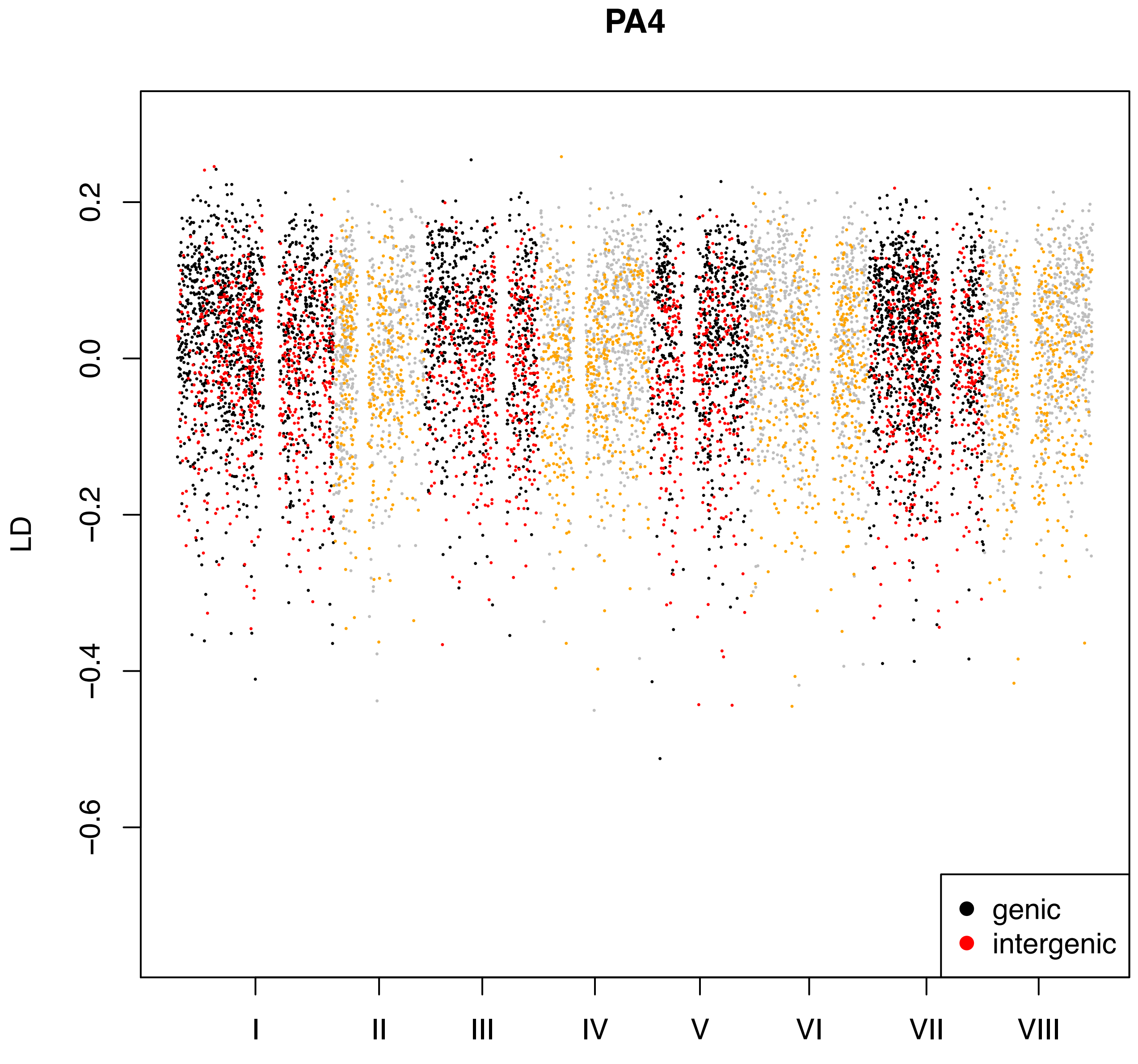
PA4
LD
0.2
0.0
−0.2
−0.4
−0.6
I
II
III
IV
V
VI
VII
VIII
genic
intergenic

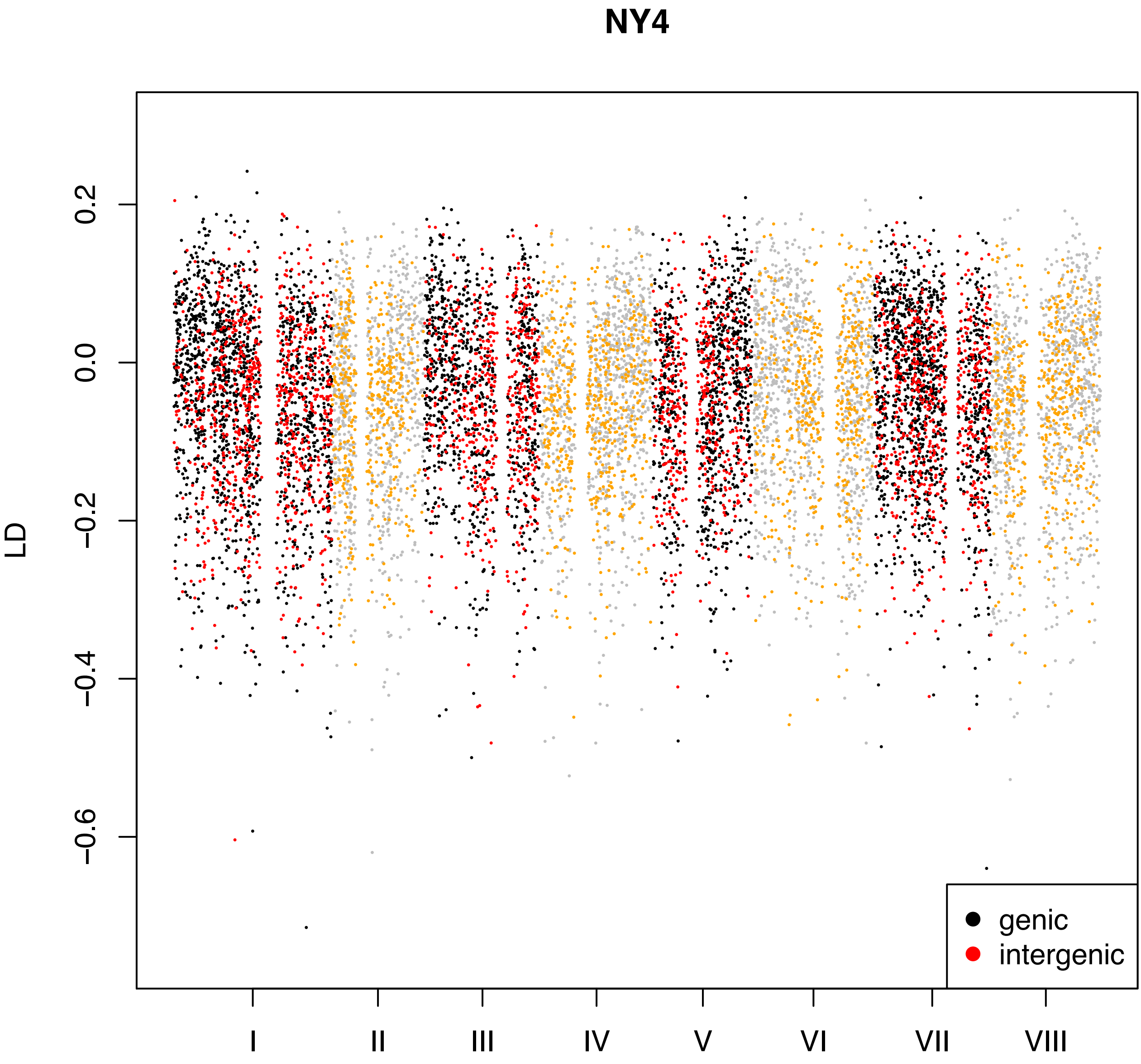
NY4
LD
0.2
0.0
−0.2
−0.4
−0.6
I
II
III
IV
V
VI
VII
VIII
genic
intergenic

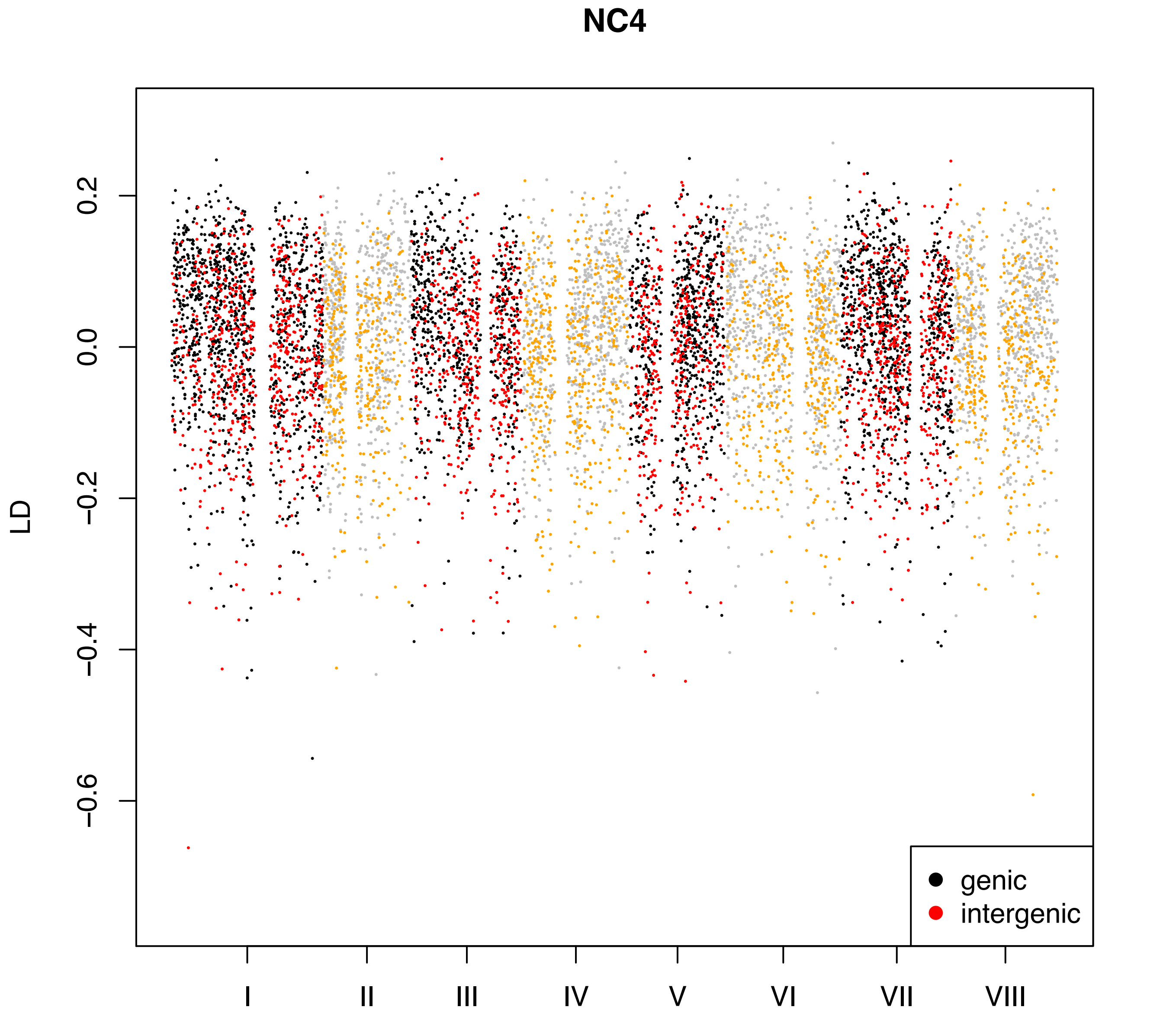
NC4
LD
0.2
0.0
−0.2
−0.4
−0.6
I
II
III
IV
V
VI
VII
VIII
genic
intergenic

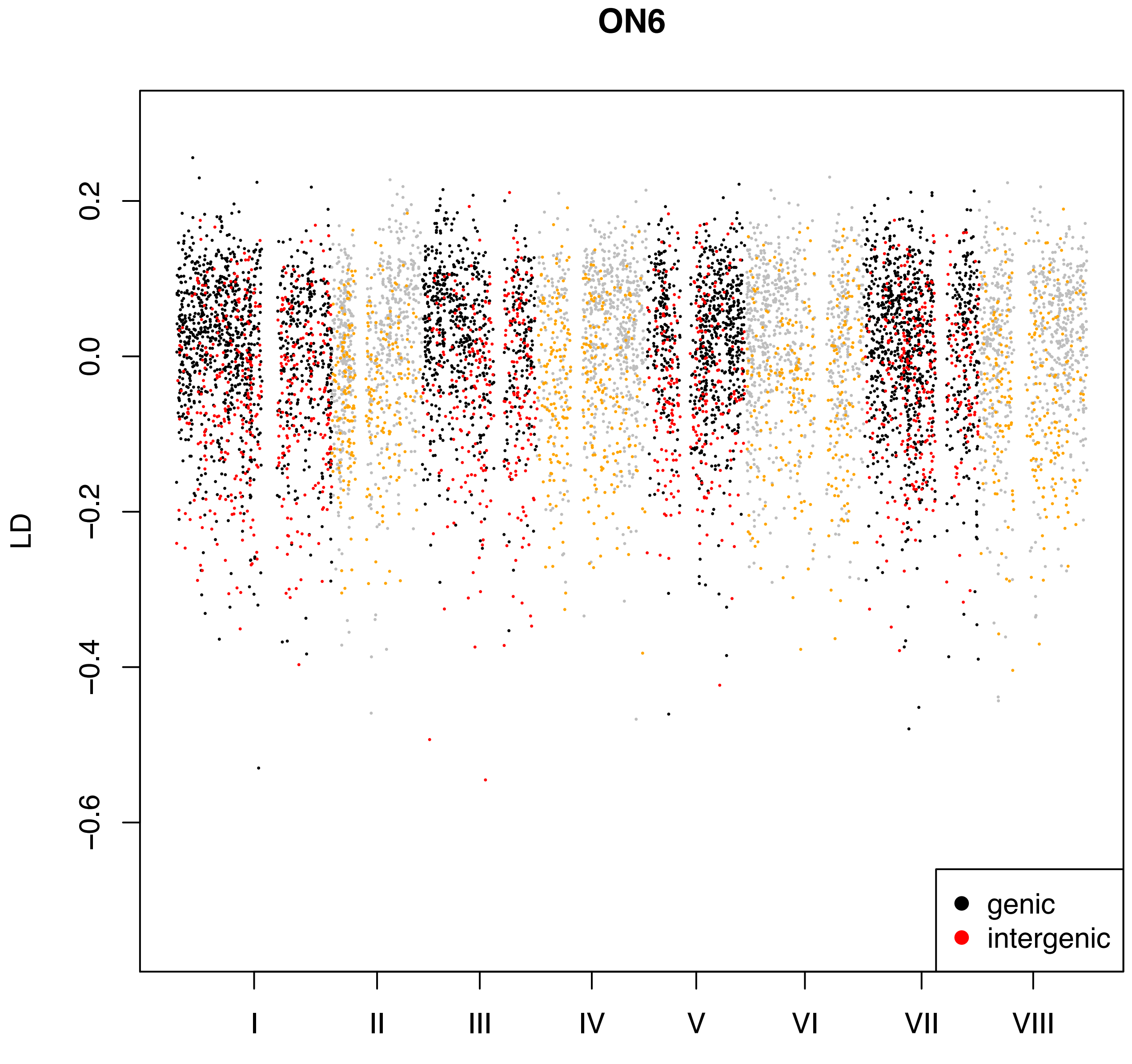
ON6
LD
0.2
0.0
−0.2
−0.4
−0.6
I
II
III
IV
V
VI
VII
VIII
genic
intergenic

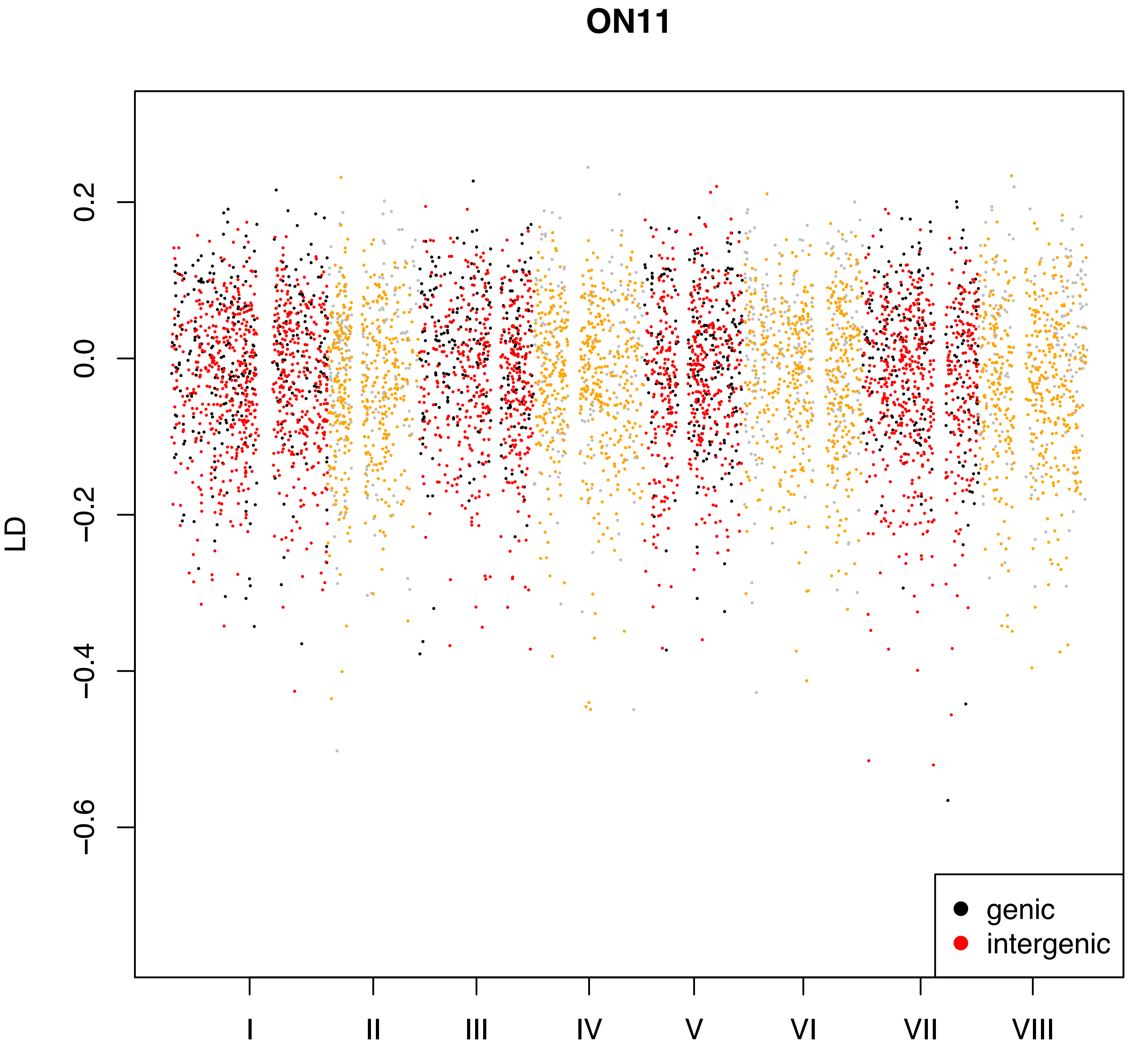
ON11
LD
0.2
0.0
−0.2
−0.4
−0.6
I
II
III
IV
V
VI
VII
VIII
genic
intergenic

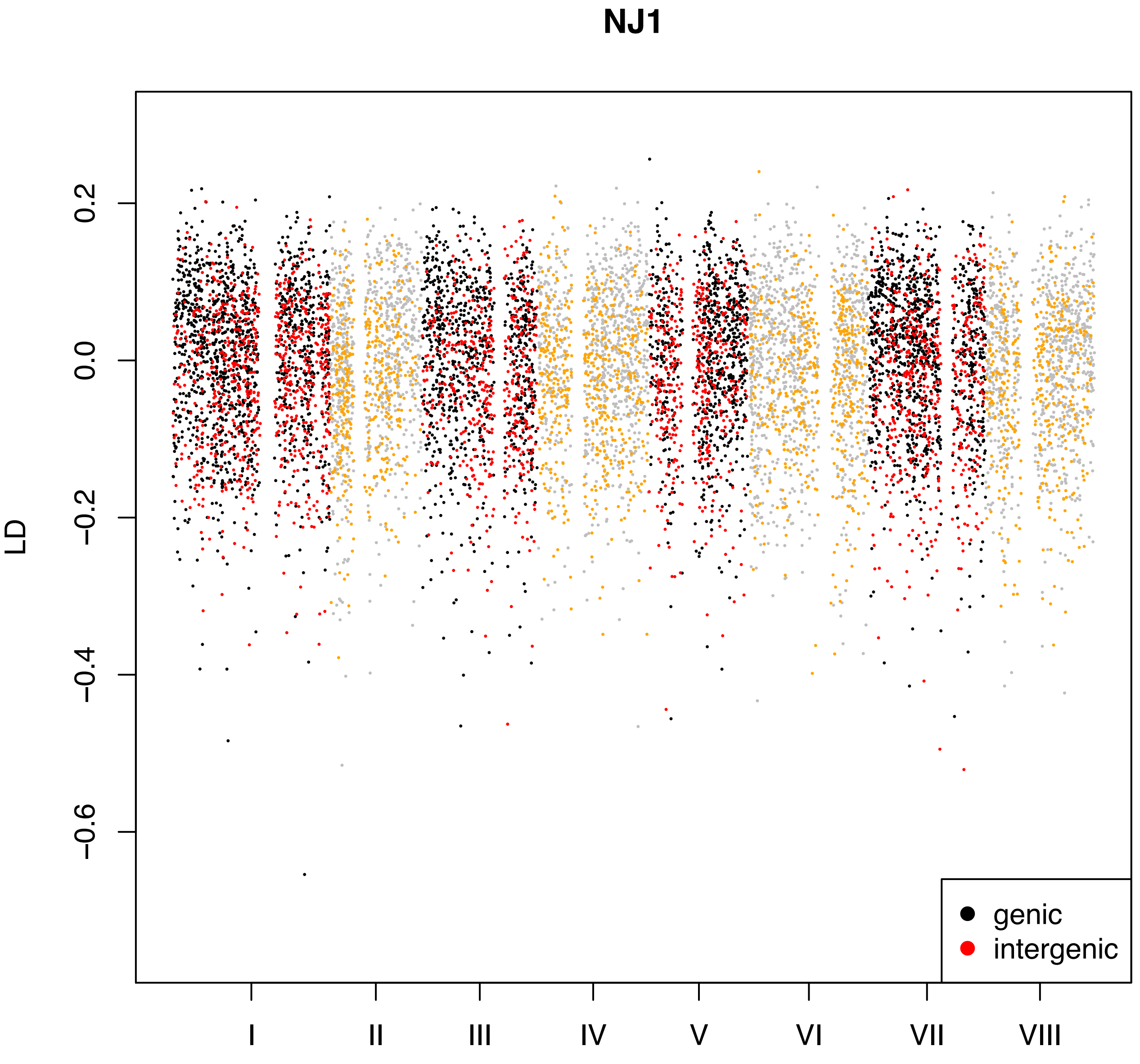
NJ1
LD
0.2
0.0
−0.2
−0.4
−0.6
I
II
III
IV
V
VI
VII
VIII
genic
intergenic

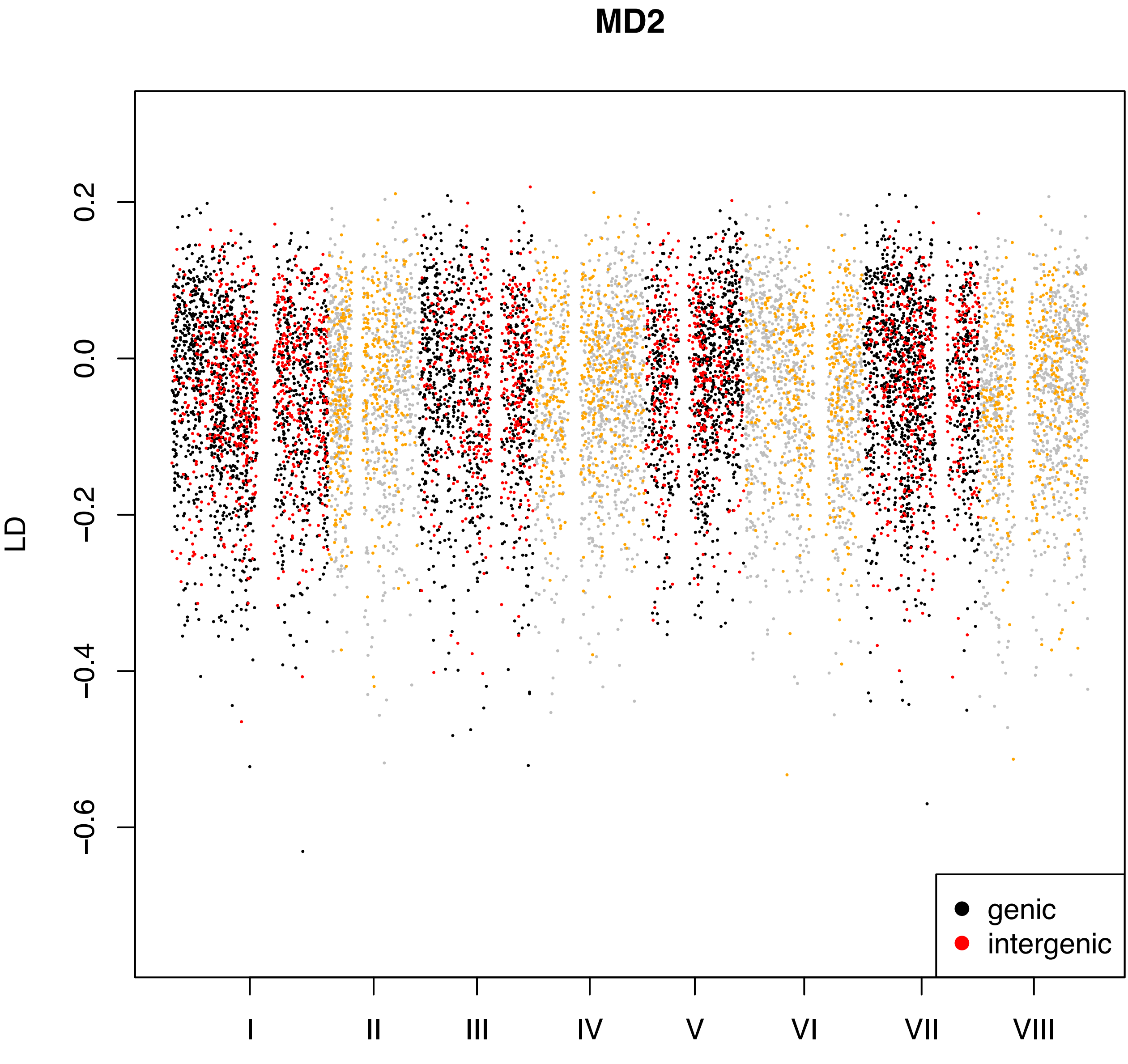
MD2
LD
0.2
0.0
−0.2
−0.4
−0.6
I
II
III
IV
V
VI
VII
VIII
genic
intergenic

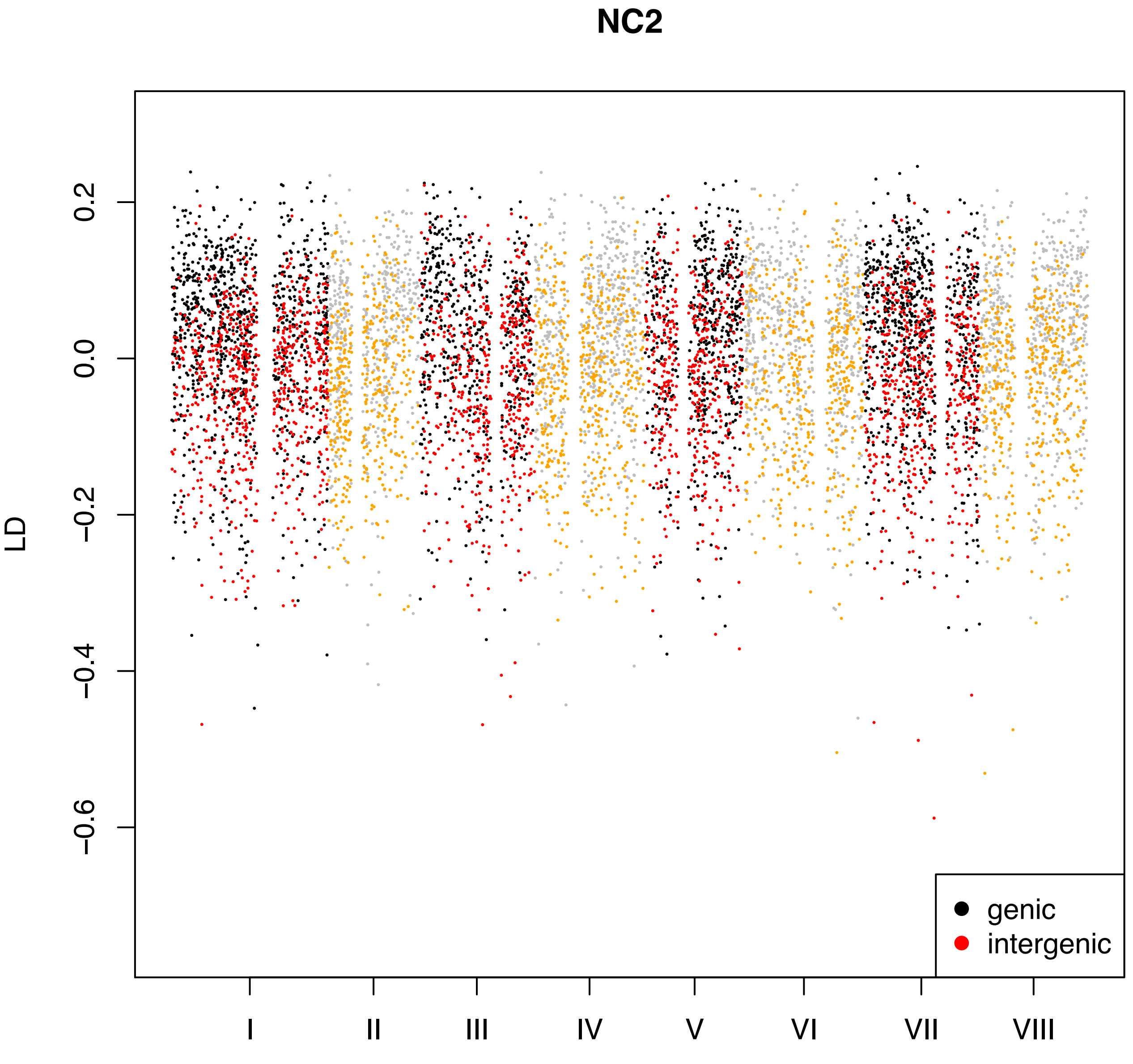
NC2
LD
0.2
0.0
-0.2
-0.4
-0.6
I
II
III
IV
V
VI
VII
VIII
genic
intergenic

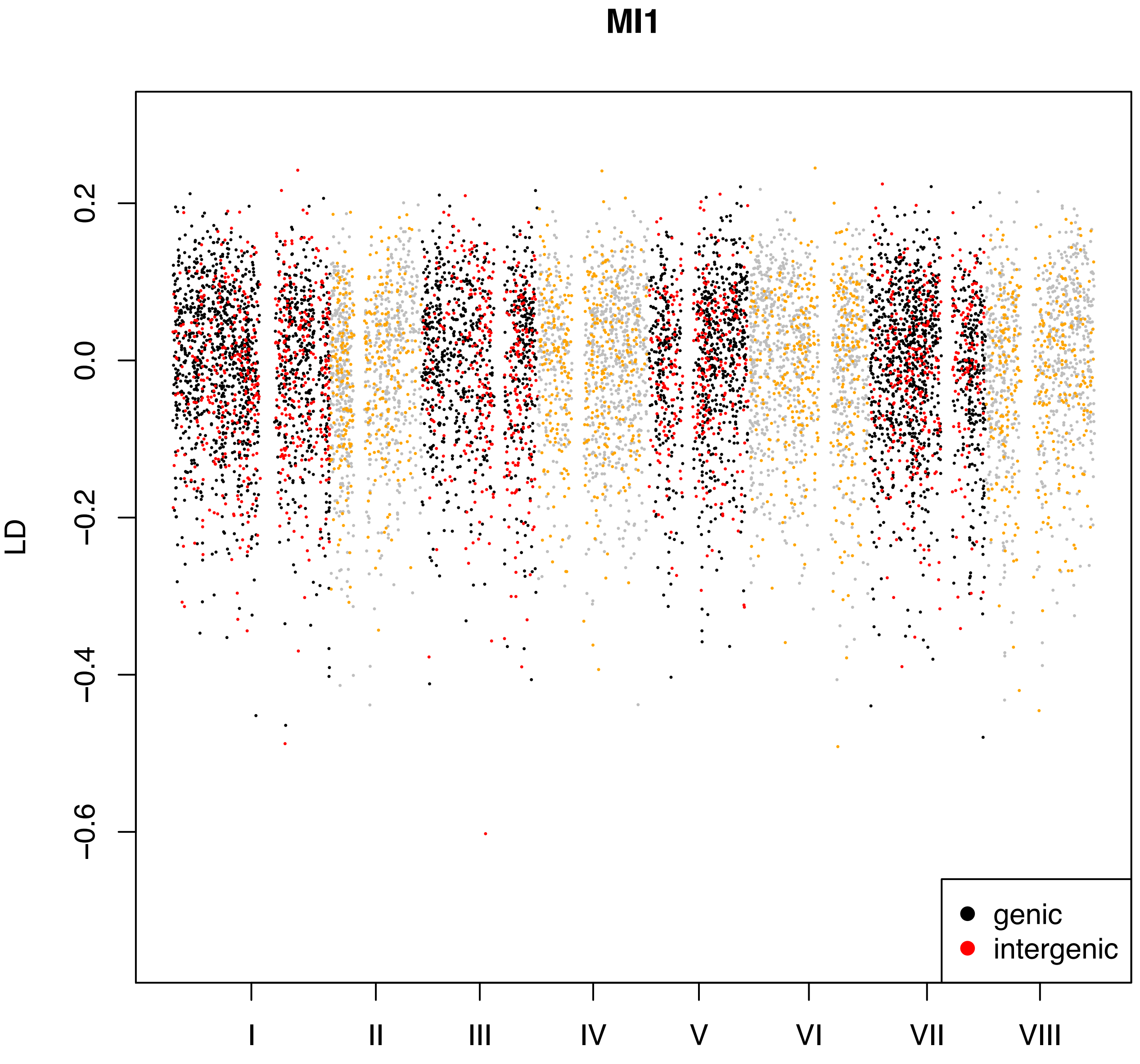

MI1
LD
0.2
0.0
−0.2
−0.4
−0.6
I
II
III
IV
V
VI
VII
VIII
genic
intergenic

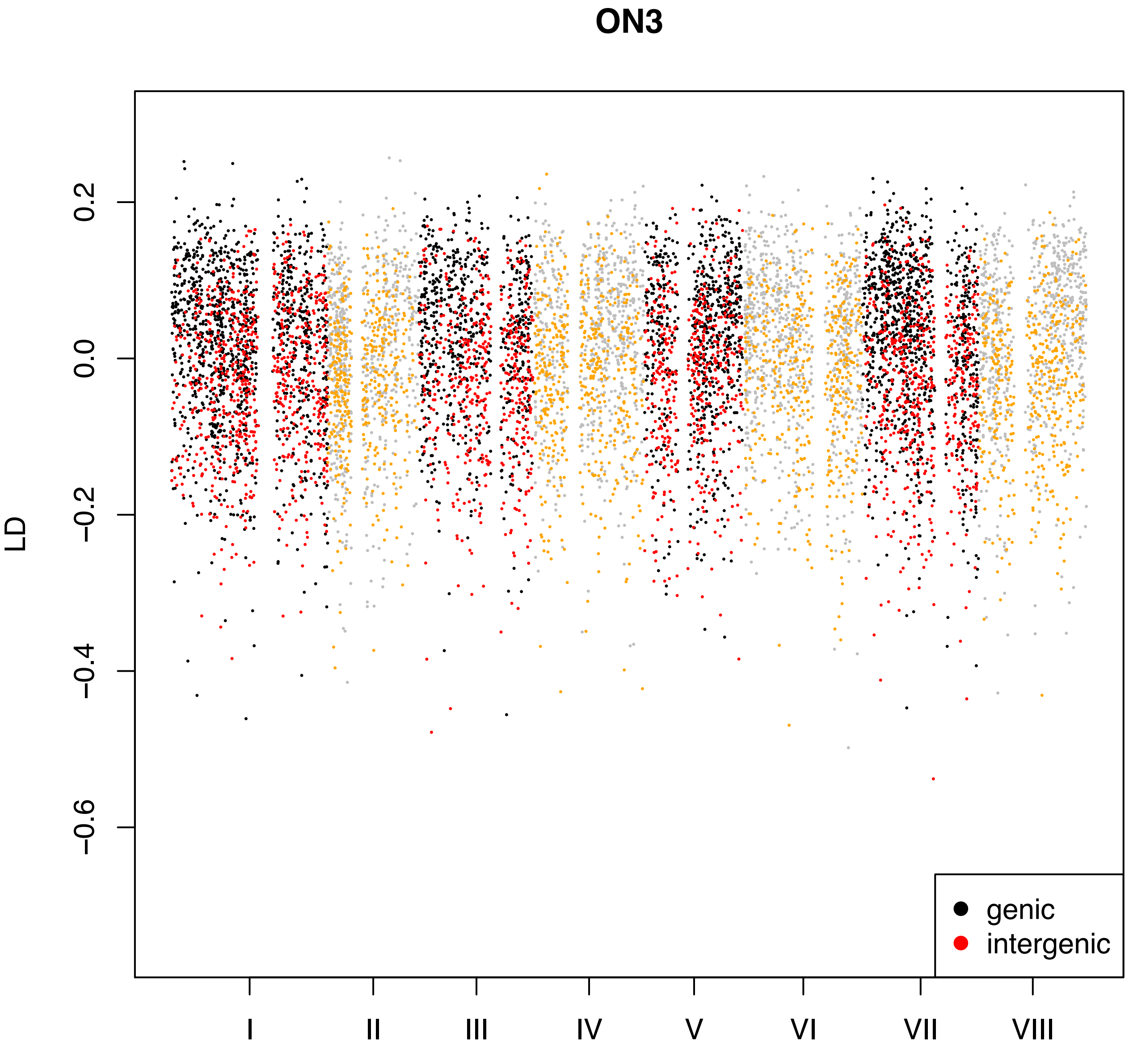
ON3
LD
0.2
0.0
−0.2
−0.4
−0.6
I
II
III
IV
V
VI
VII
VIII
genic
intergenic

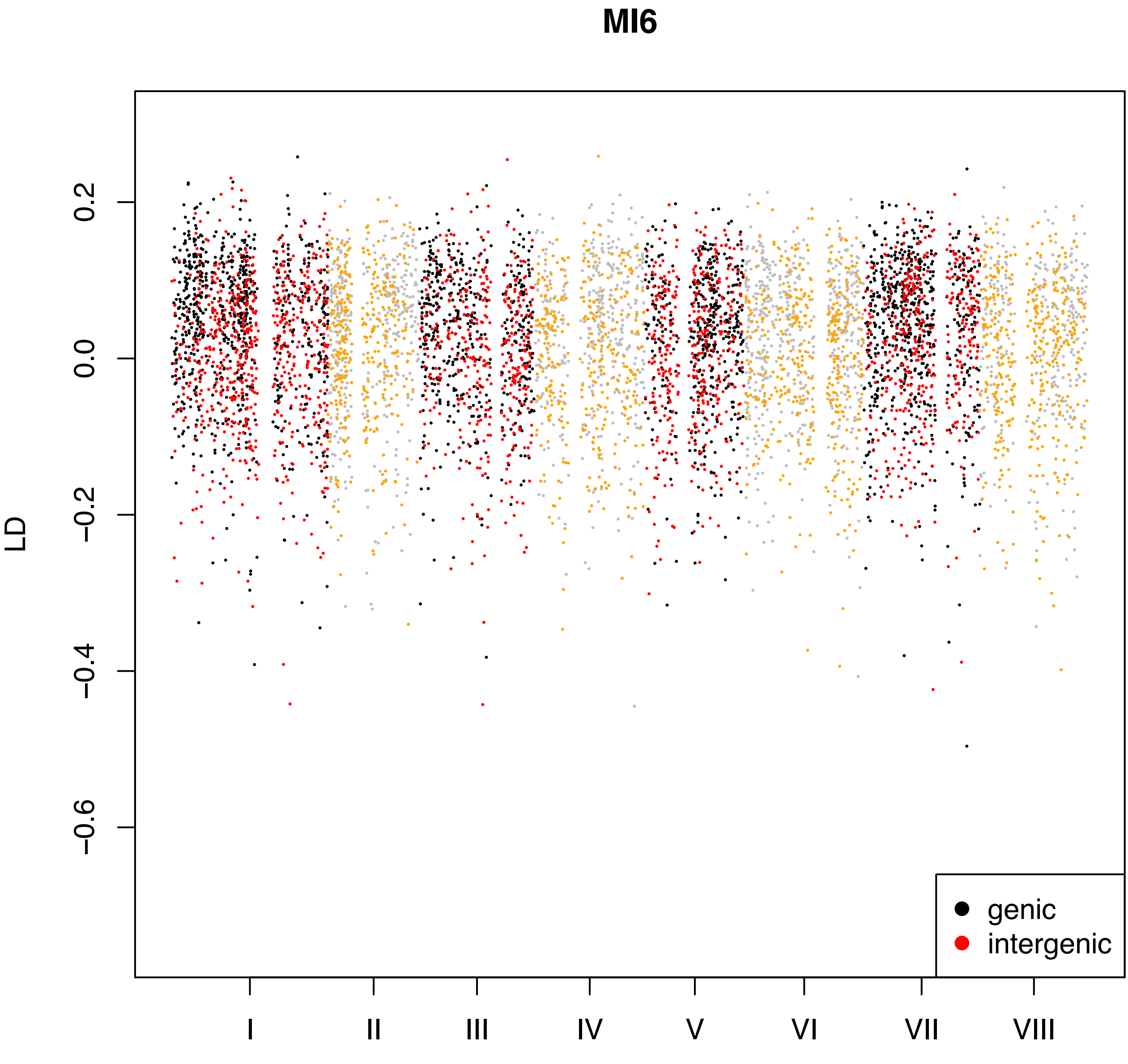
MI6
LD
0.2
0.0
−0.2
−0.4
−0.6
I
II
III
IV
V
VI
VII
VIII
genic
intergenic

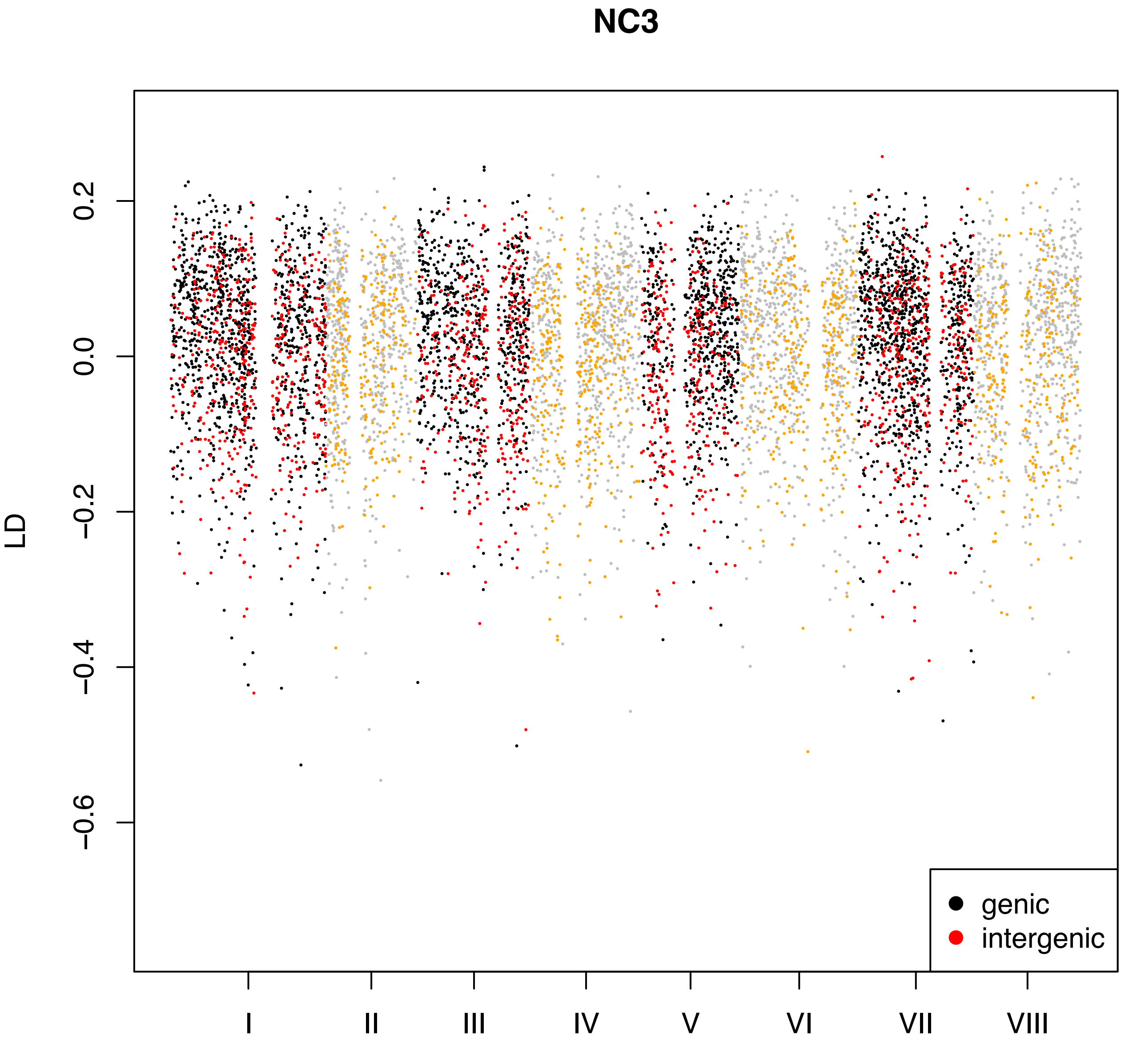
NC3
LD
0.2
0.0
−0.2
−0.4
−0.6
I
II
III
IV
V
VI
VII
VIII
genic
intergenic

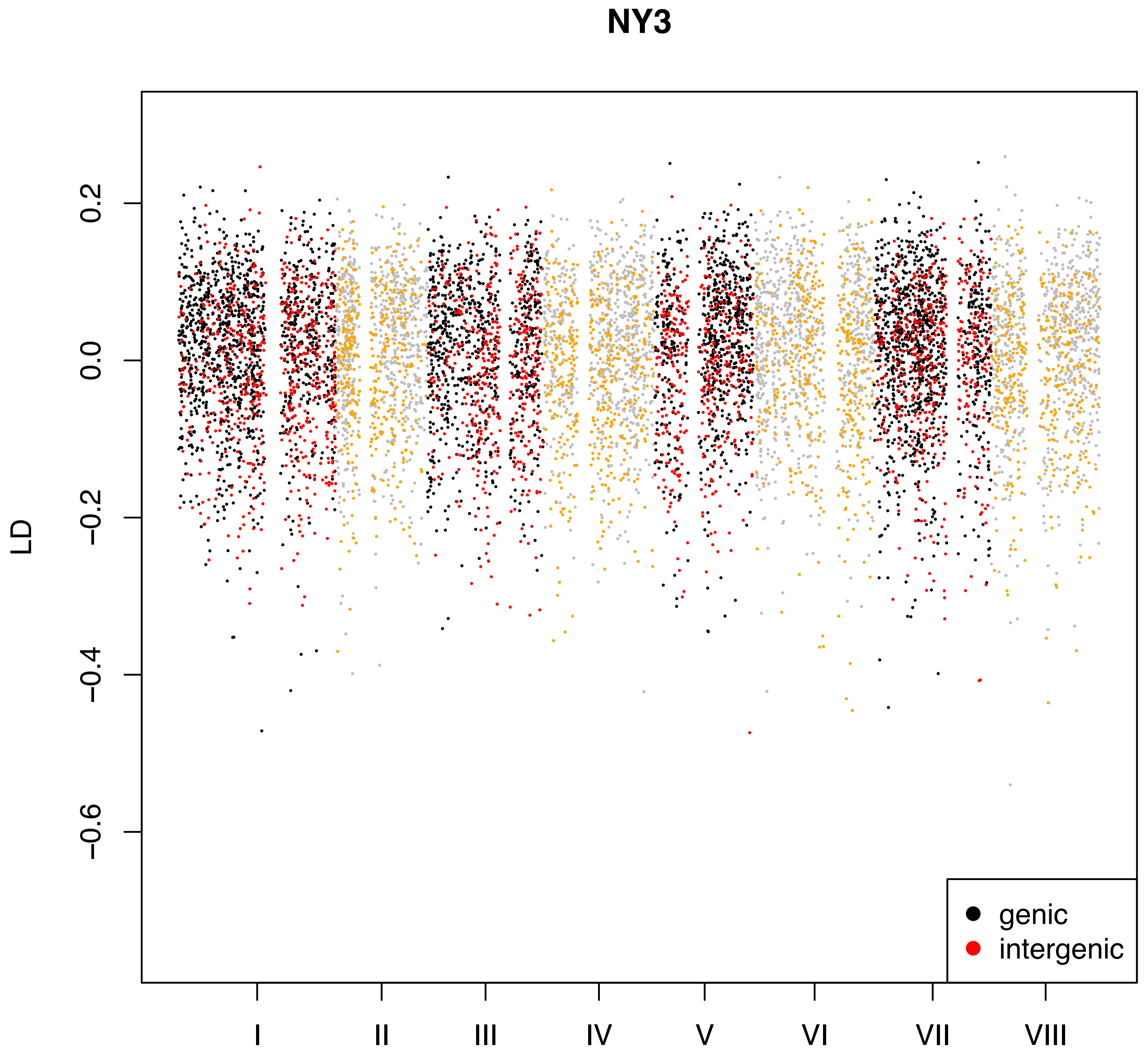
NY3
LD
0.2
0.0
−0.2
−0.4
−0.6
I
II
III
IV
V
VI
VII
VIII
genic
intergenic

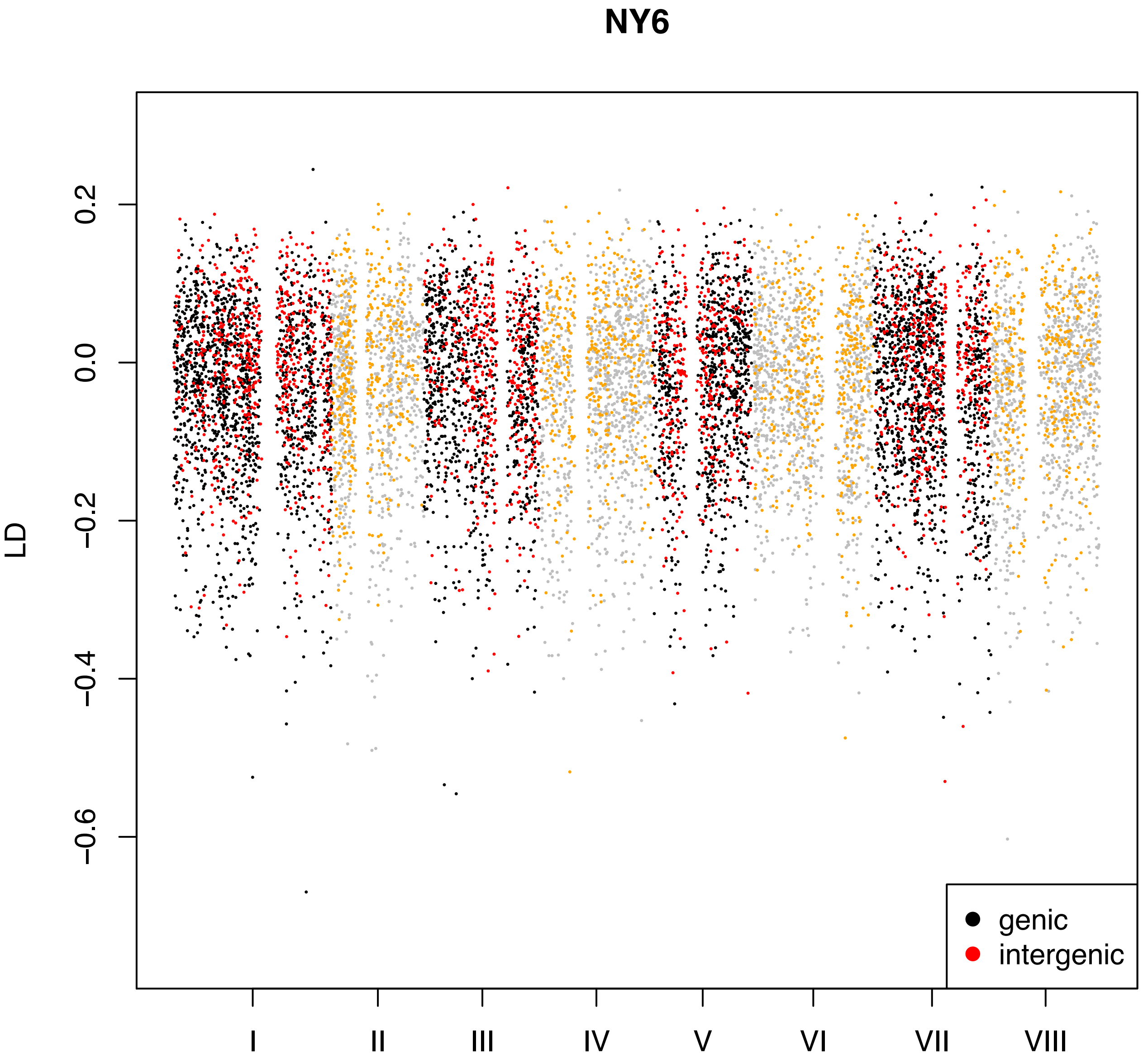
NY6
LD
0.2
0.0
-0.2
-0.4
-0.6
I
II
III
IV
V
VI
VII
VIII
genic
intergenic

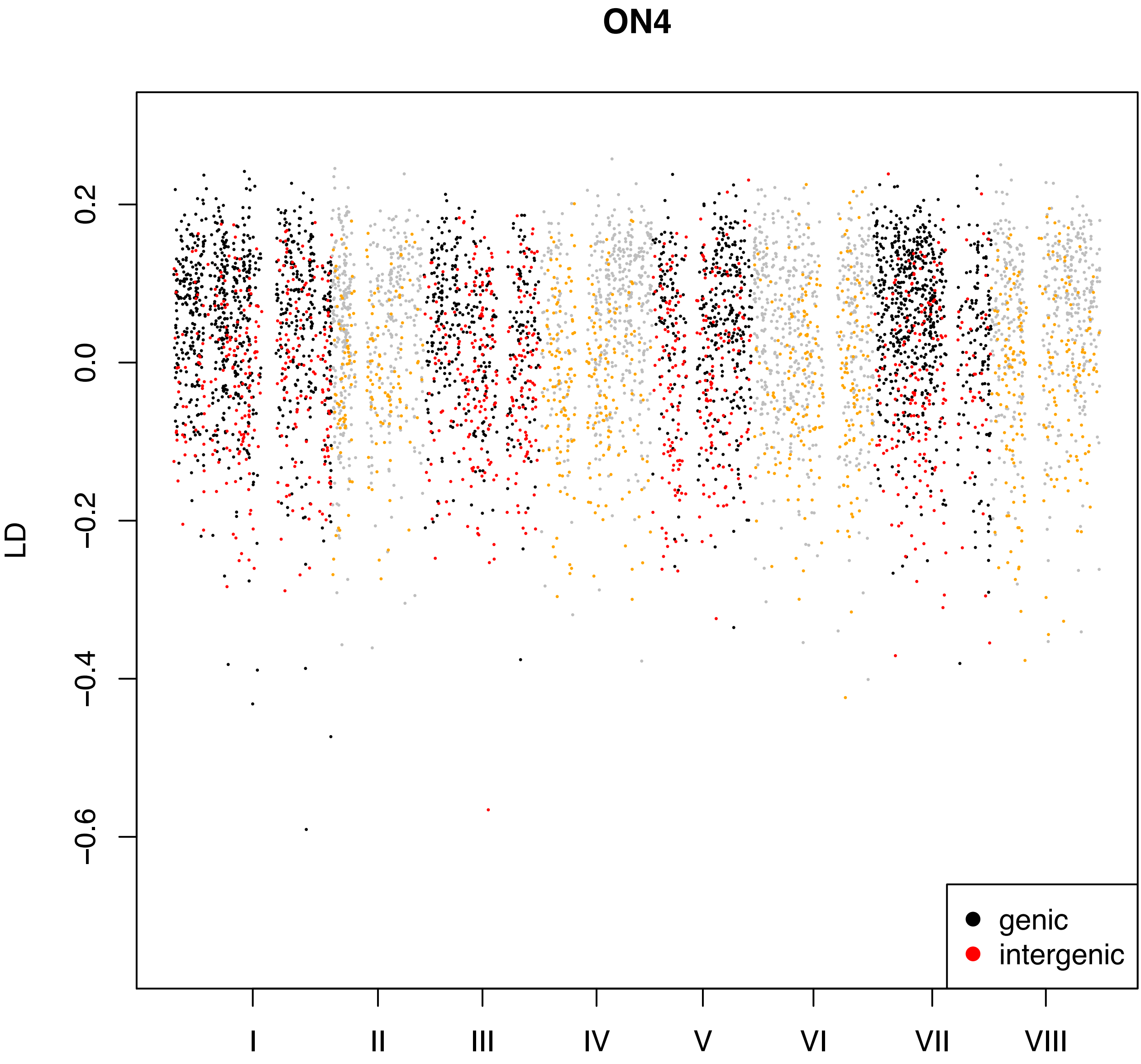

ON4
LD
0.2
0.0
−0.2
−0.4
−0.6
I
II
III
IV
V
VI
VII
VIII
genic
intergenic

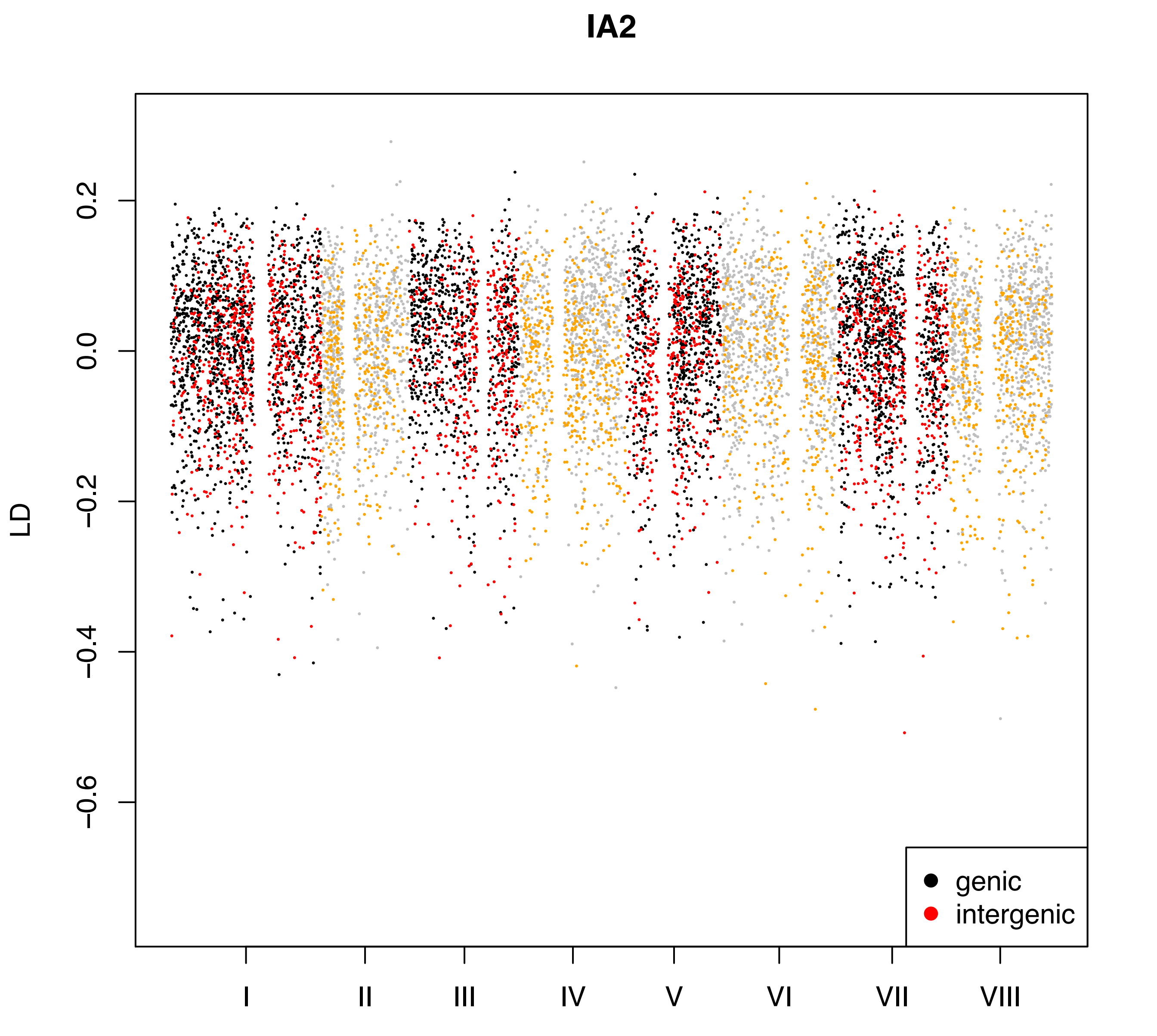

IA2
LD
0.2
0.0
−0.2
−0.4
−0.6
I
II
III
IV
V
VI
VII
VIII
genic
intergenic

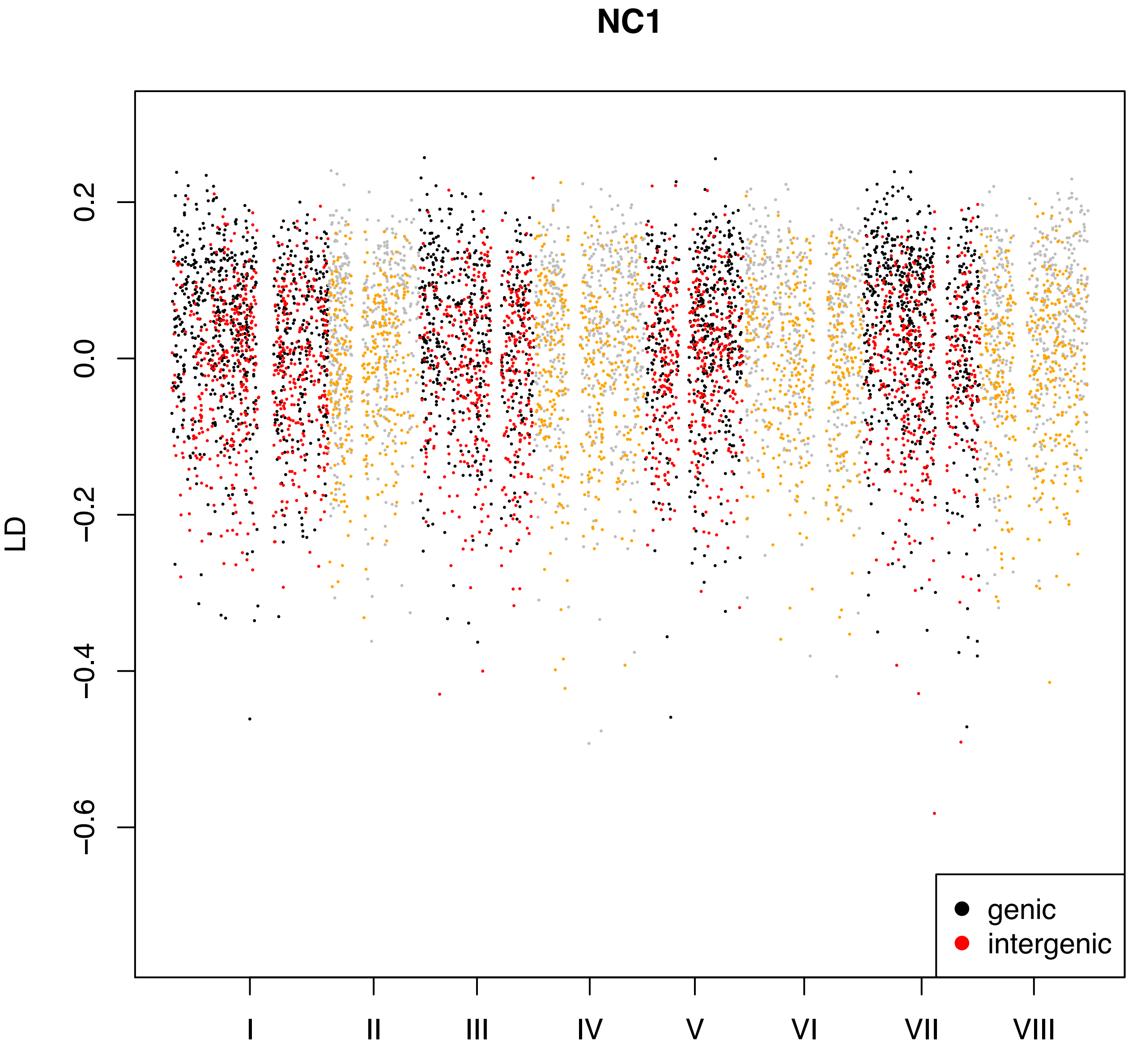
NC1
LD
0.2
0.0
-0.2
-0.4
-0.6
I
II
III
IV
V
VI
VII
VIII
genic
intergenic

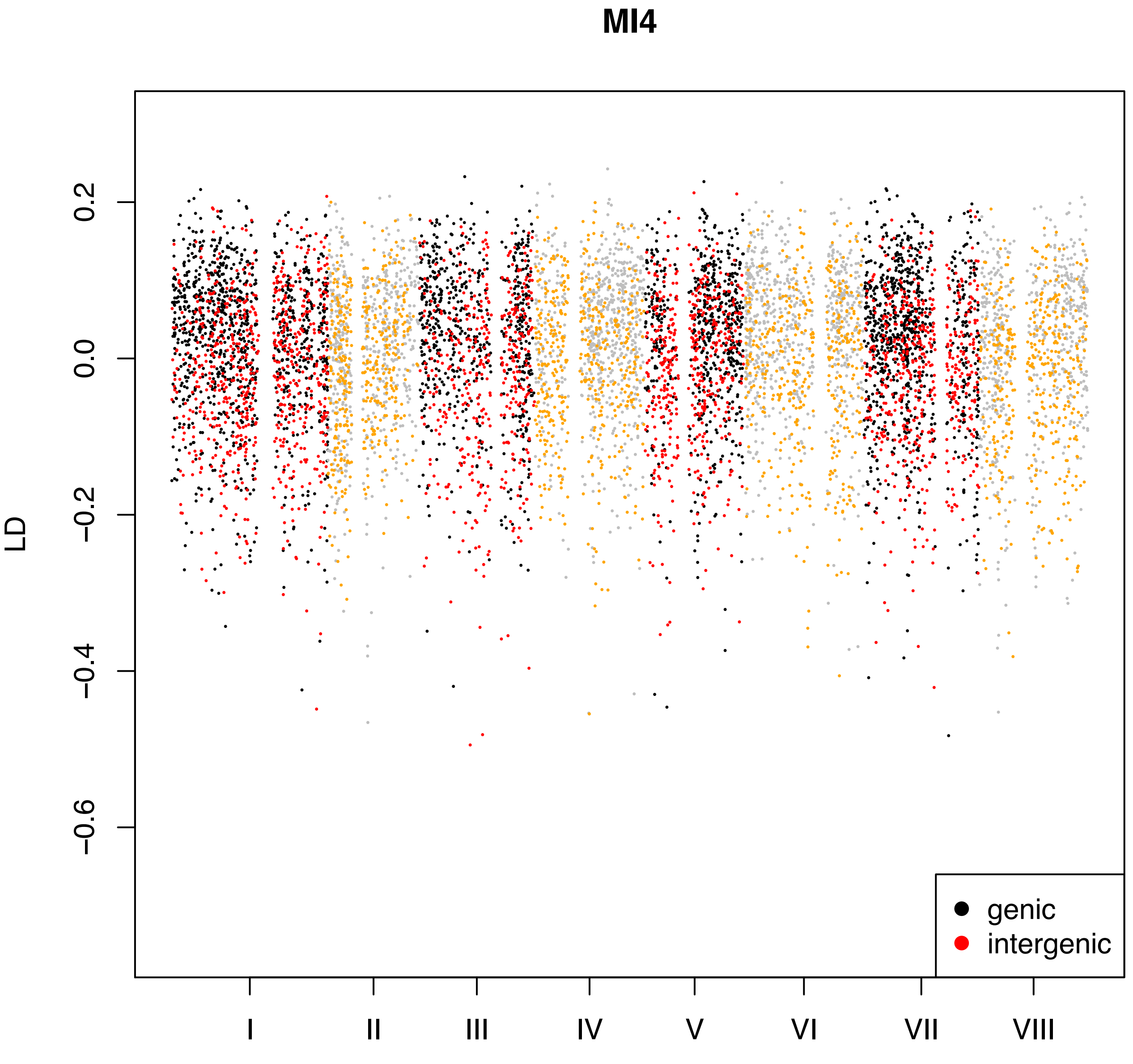
MI4
LD
0.2
0.0
−0.2
−0.4
−0.6
I
II
III
IV
V
VI
VII
VIII
genic
intergenic

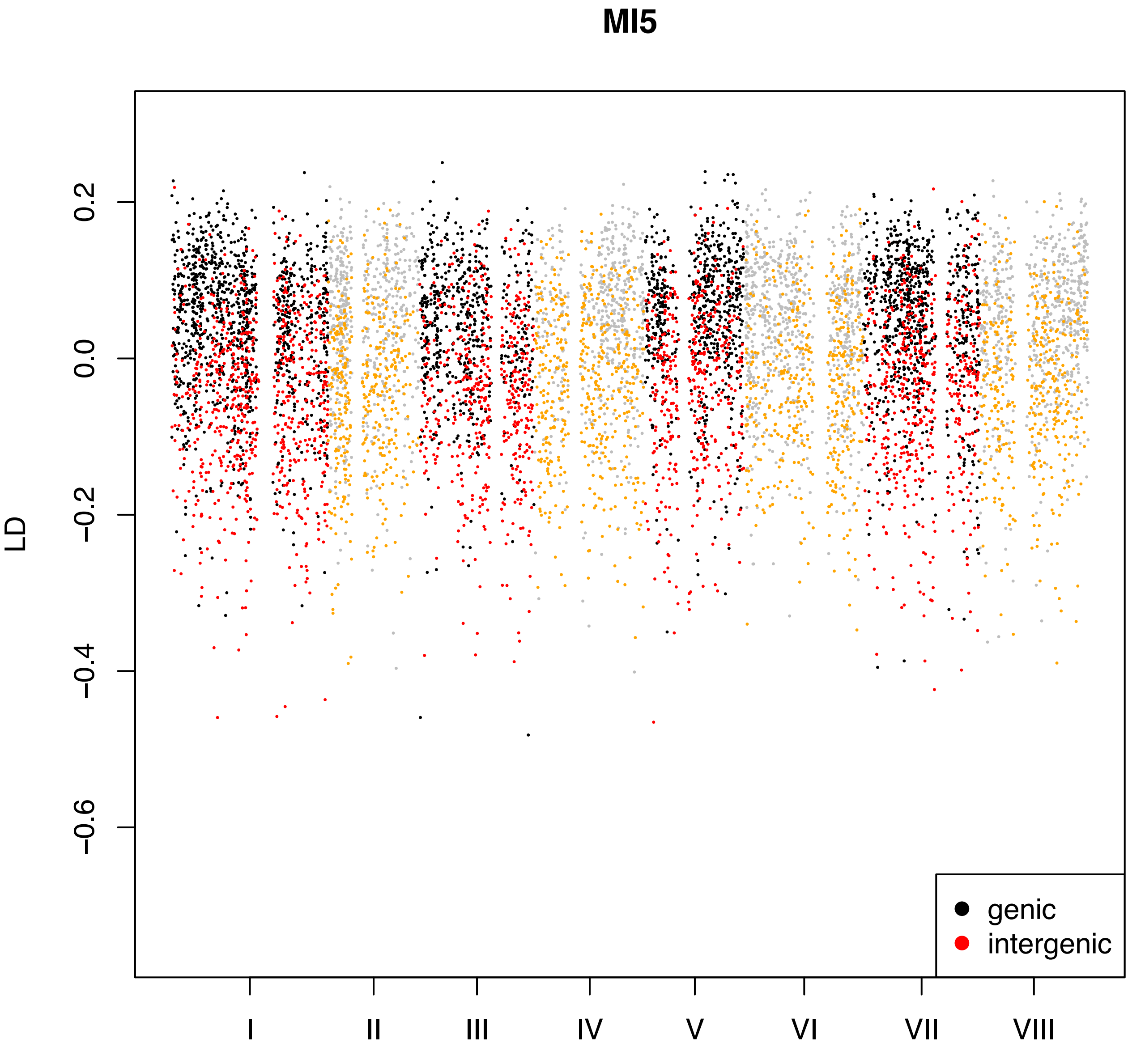

MI5
LD
0.2
0.0
−0.2
−0.4
−0.6
I
II
III
IV
V
VI
VII
VIII
genic
intergenic

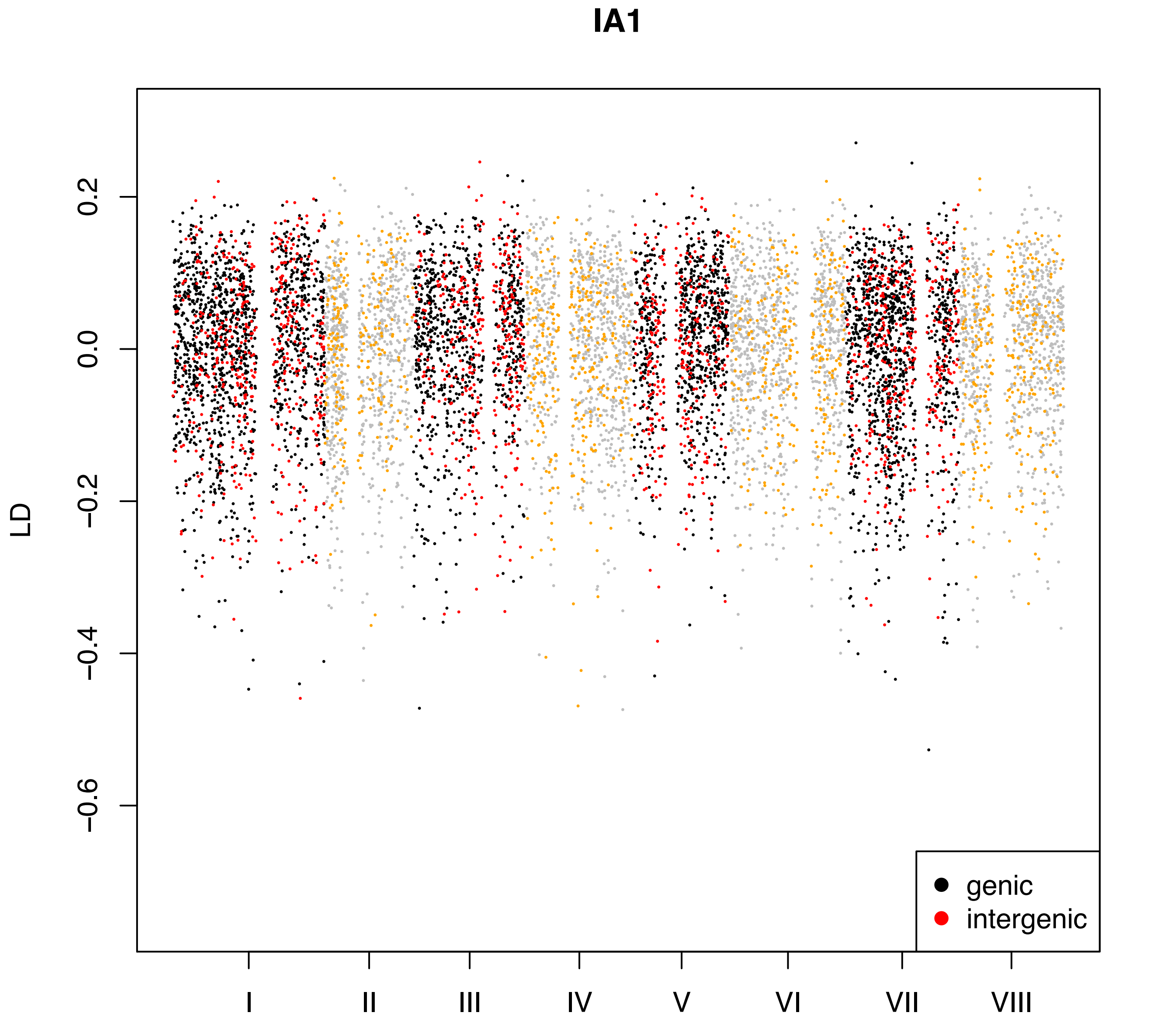

IA1
LD
0.2
0.0
−0.2
−0.4
−0.6
I
II
III
IV
V
VI
VII
VIII
genic
intergenic

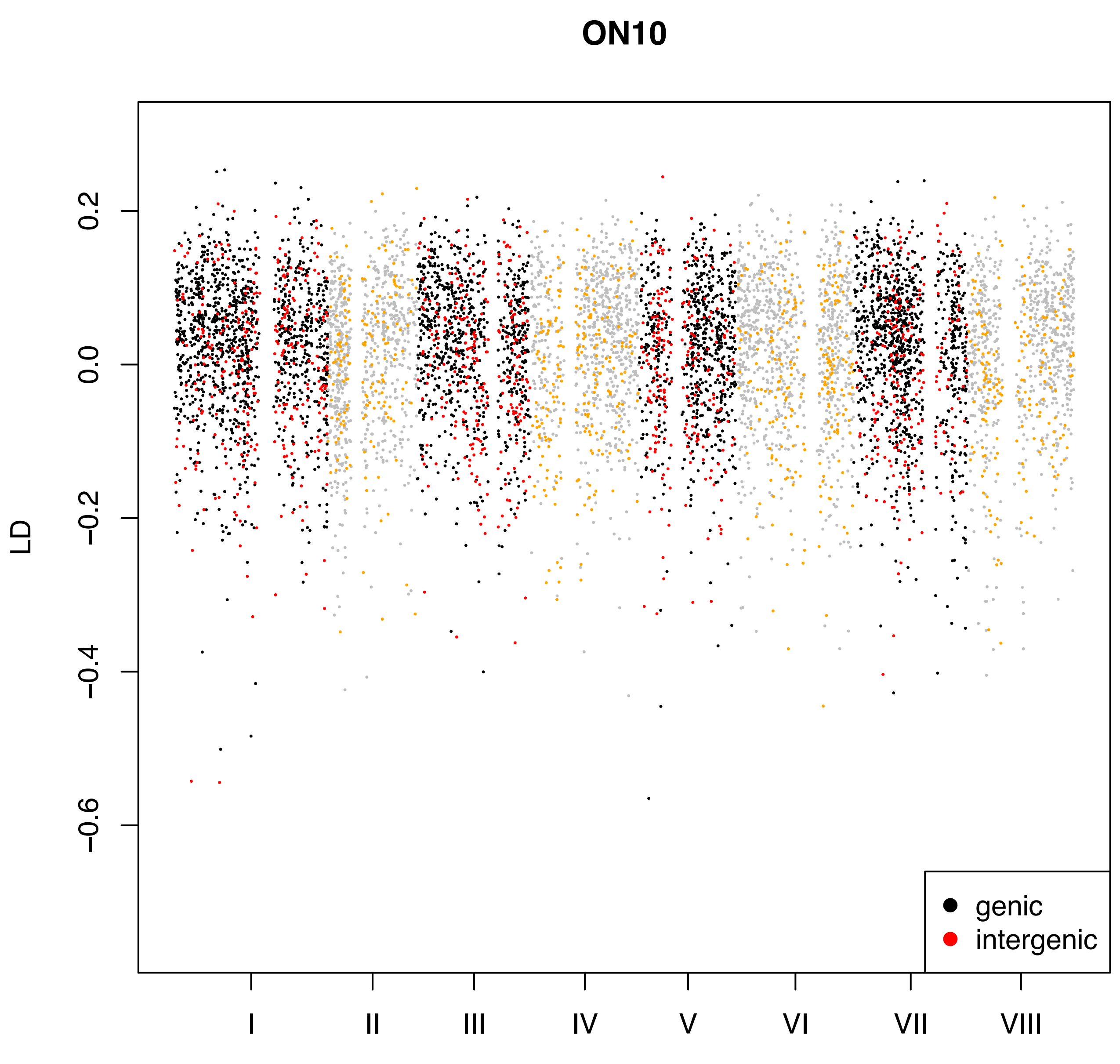

ON10
LD
0.2
0.0
−0.2
−0.4
−0.6
I
II
III
IV
V
VI
VII
VIII
genic
intergenic

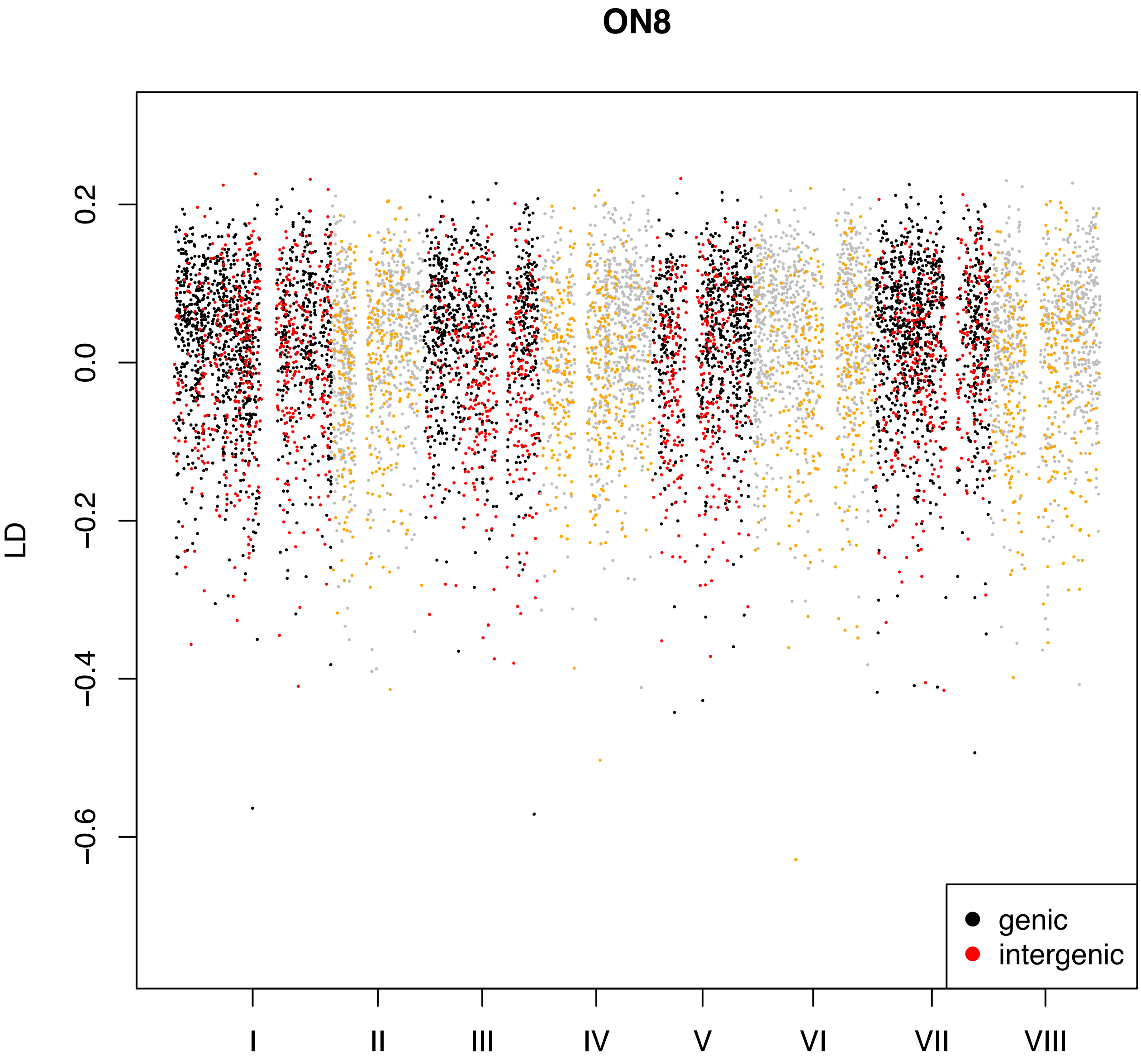
ON8
LD
0.2
0.0
-0.2
-0.4
-0.6
I
II
III
IV
V
VI
VII
VIII
genic
intergenic

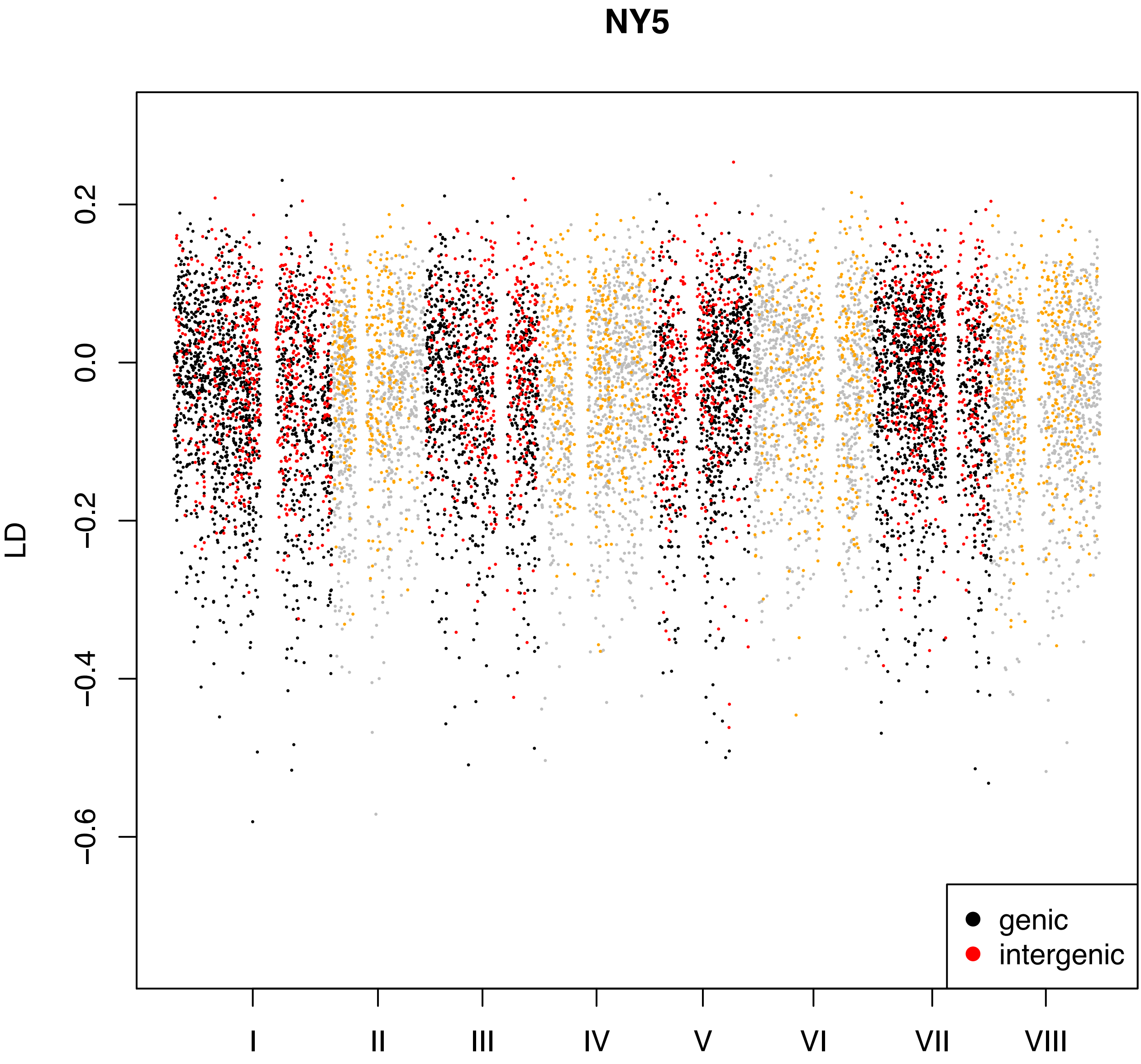

NY5
LD
0.2
0.0
−0.2
−0.4
−0.6
I
II
III
IV
V
VI
VII
VIII
genic
intergenic

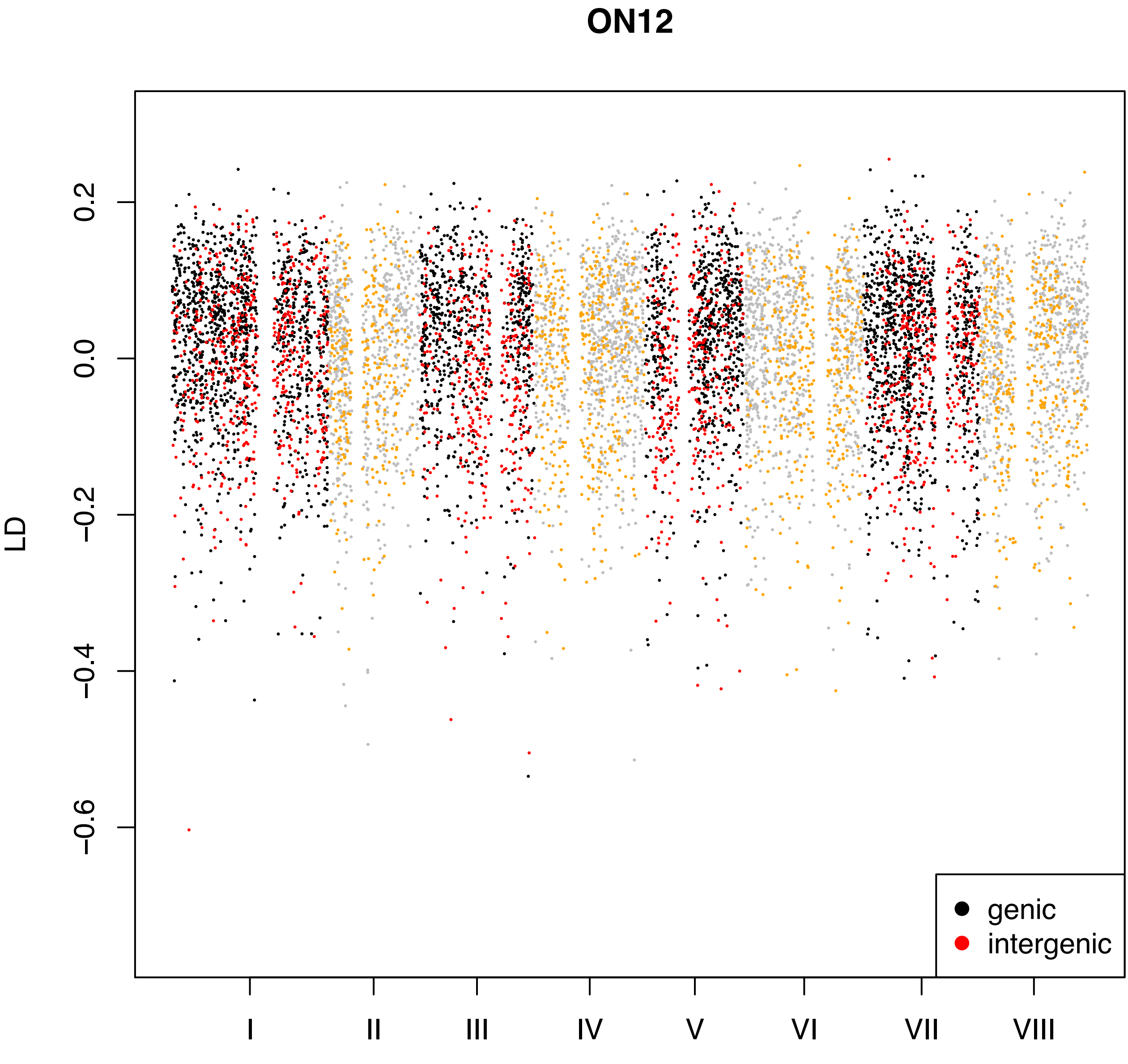
ON12
LD
0.2
0.0
-0.2
-0.4
-0.6
I
II
III
IV
V
VI
VII
VIII
genic
intergenic

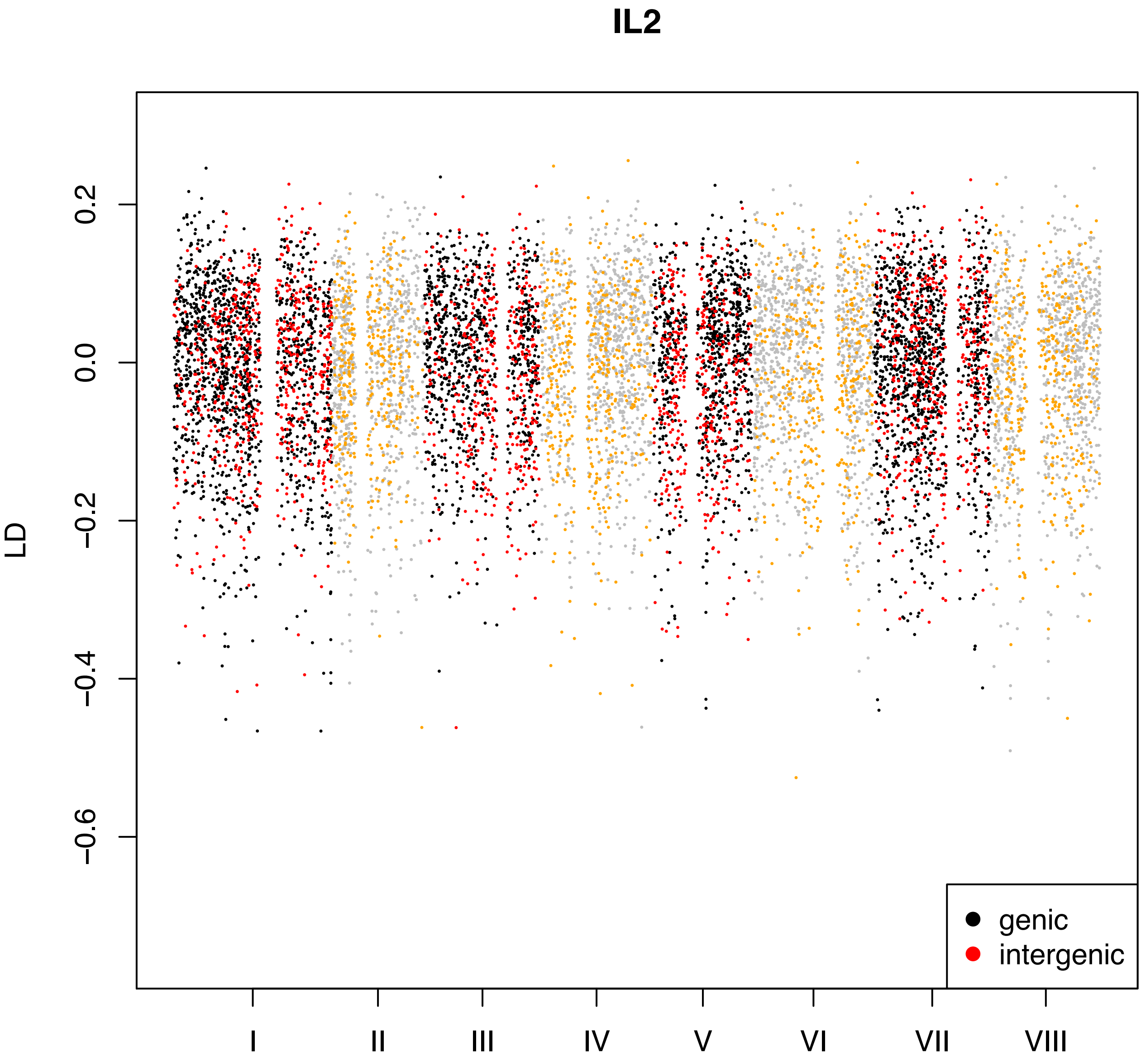

IL2
LD
0.2
0.0
-0.2
-0.4
-0.6
I
II
III
IV
V
VI
VII
VIII
genic
intergenic

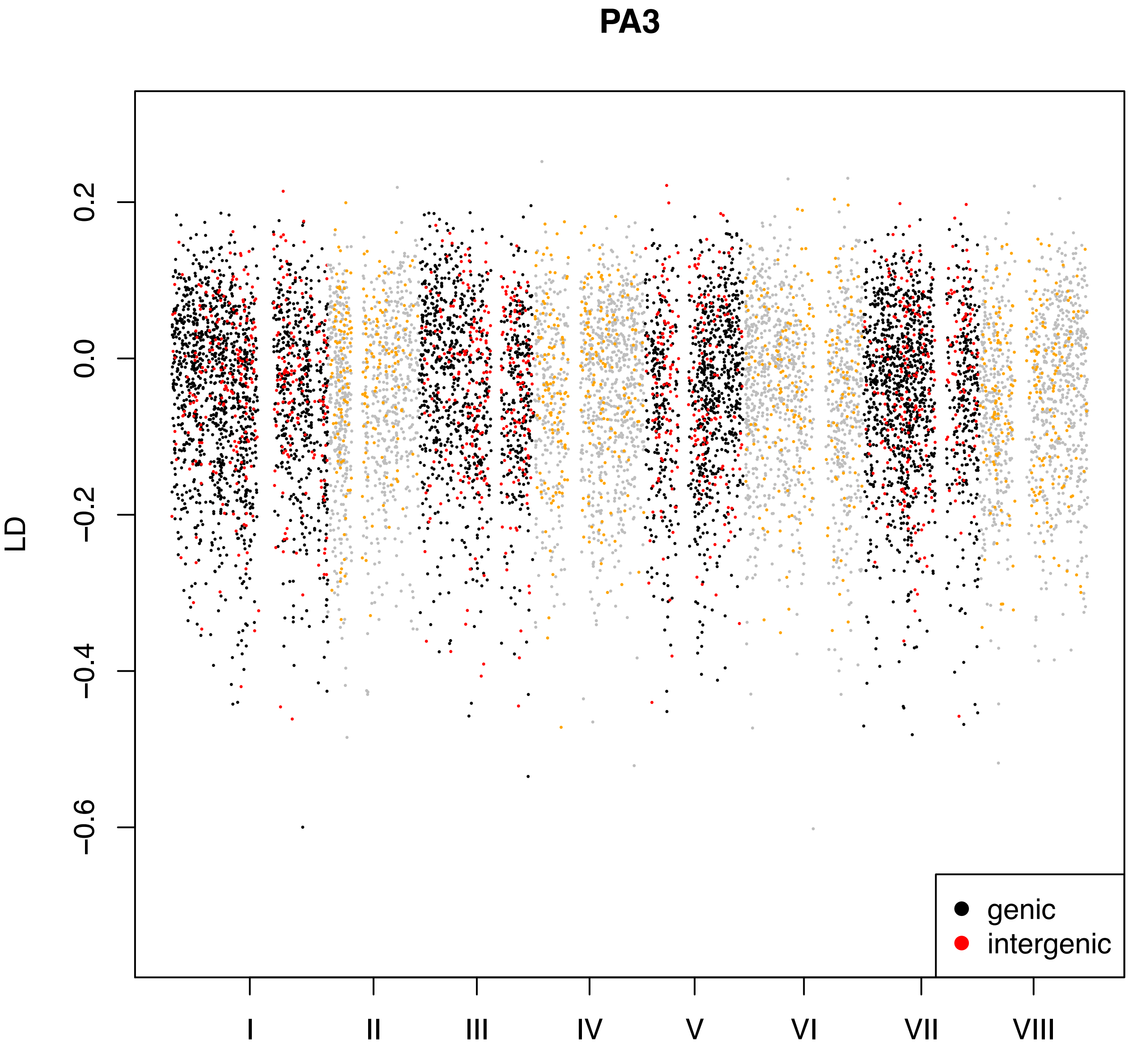

PA3
LD
0.2
0.0
−0.2
−0.4
−0.6
I
II
III
IV
V
VI
VII
VIII
genic
intergenic

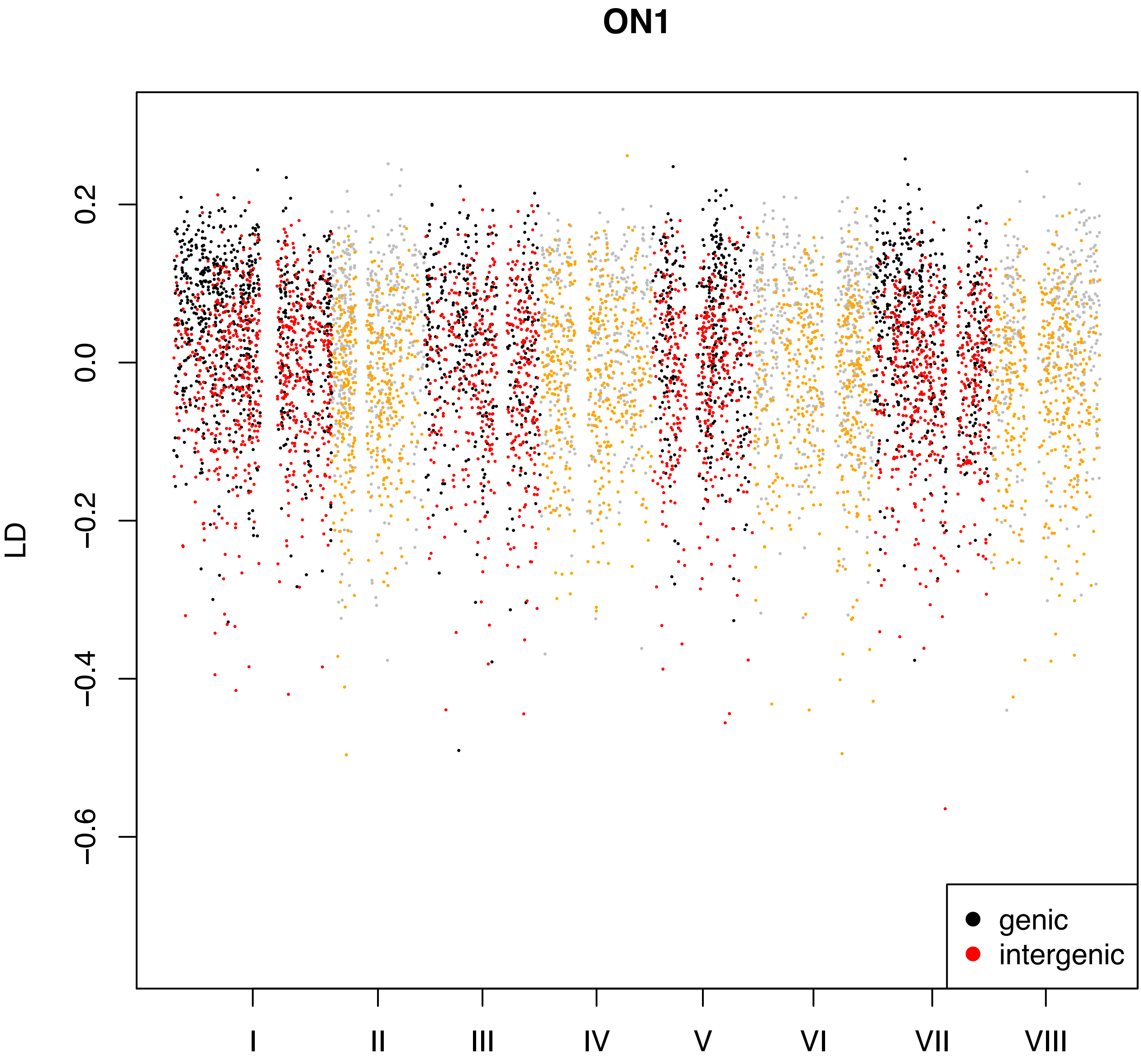
ON1
LD
0.2
0.0
−0.2
−0.4
−0.6
I
II
III
IV
V
VI
VII
VIII
genic
intergenic

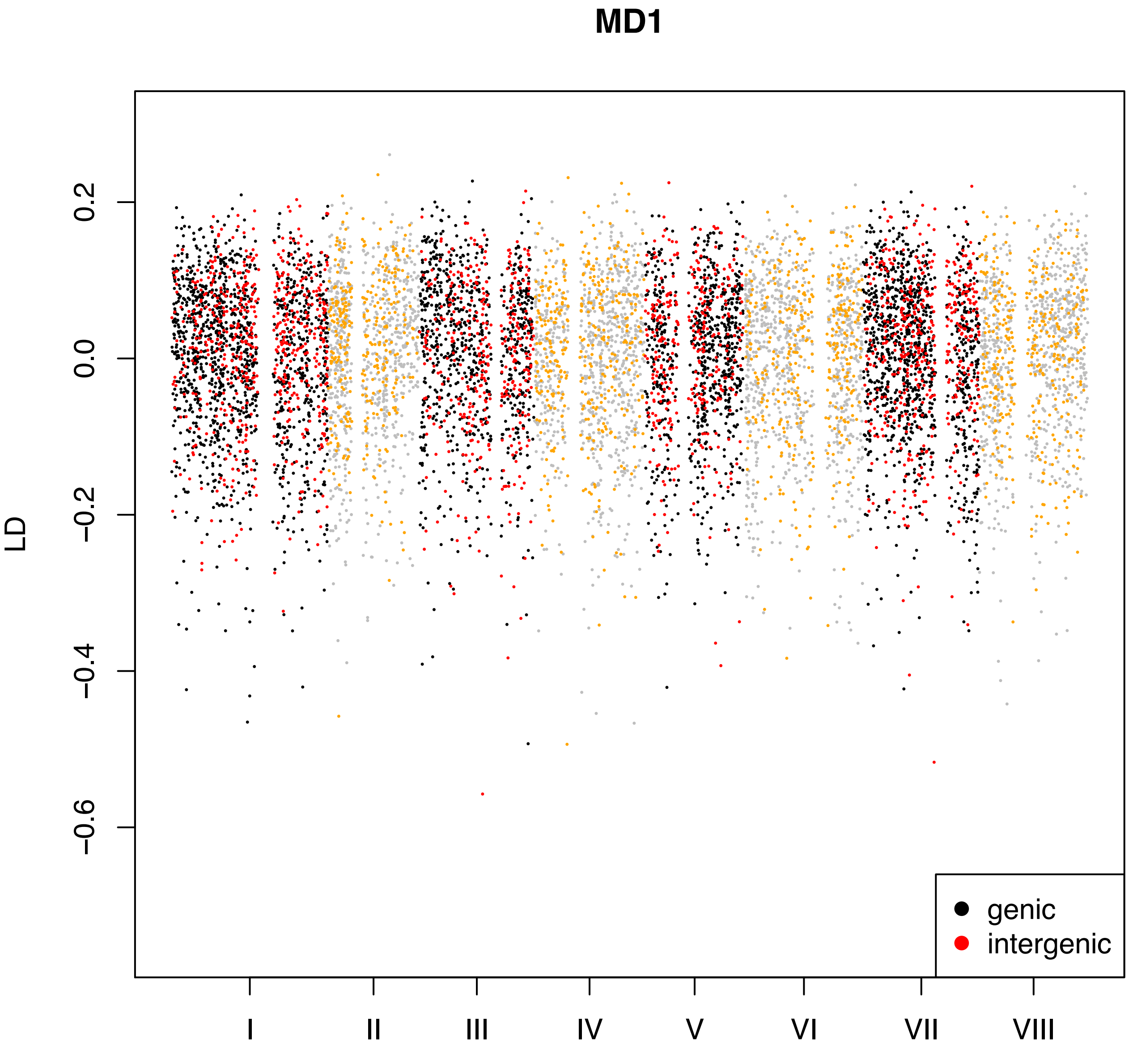

MD1
LD
0.2
0.0
−0.2
−0.4
−0.6
I
II
III
IV
V
VI
VII
VIII
genic
intergenic

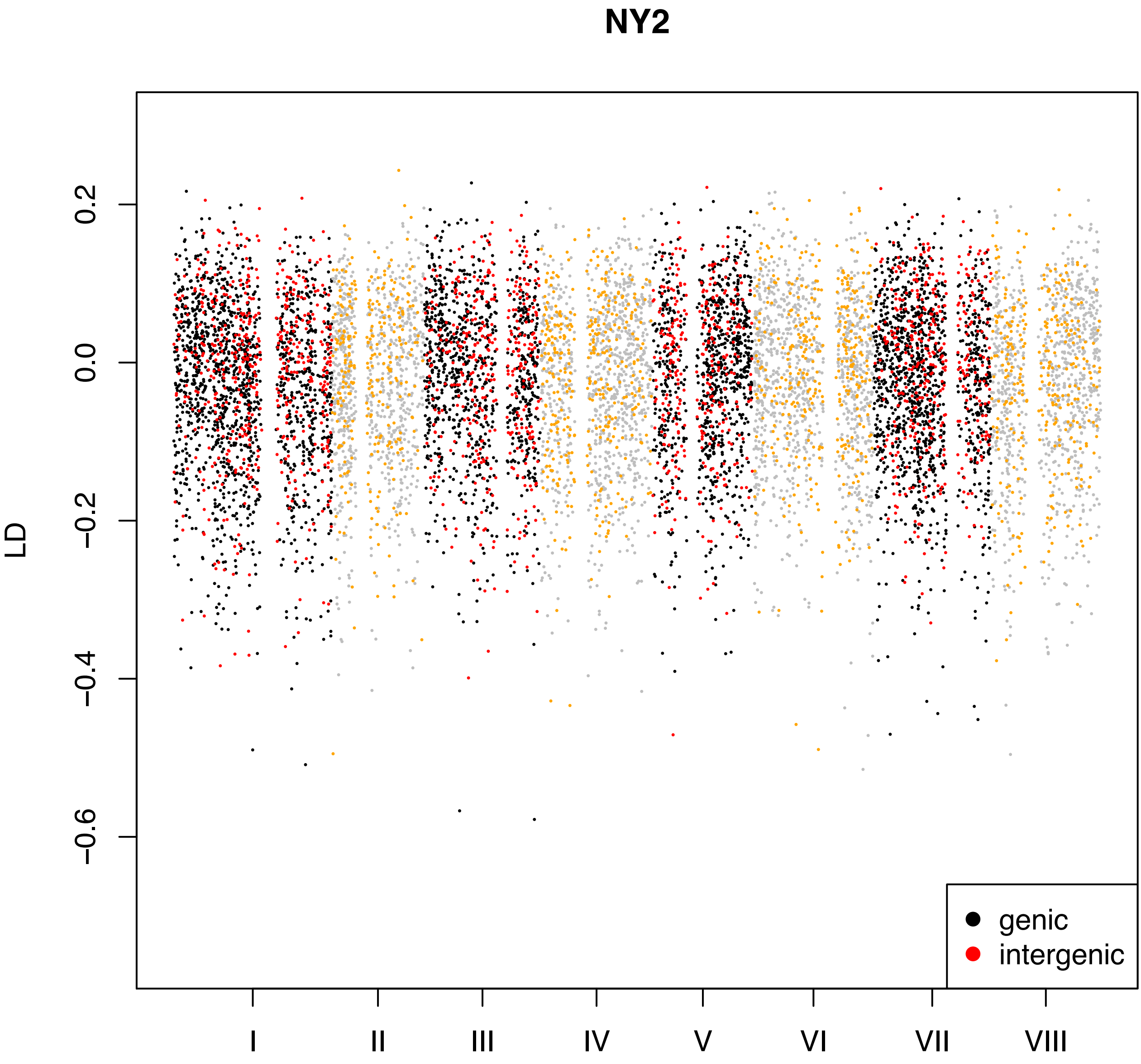
NY2
LD
0.2
0.0
-0.2
-0.4
-0.6
I
II
III
IV
V
VI
VII
VIII
genic
intergenic

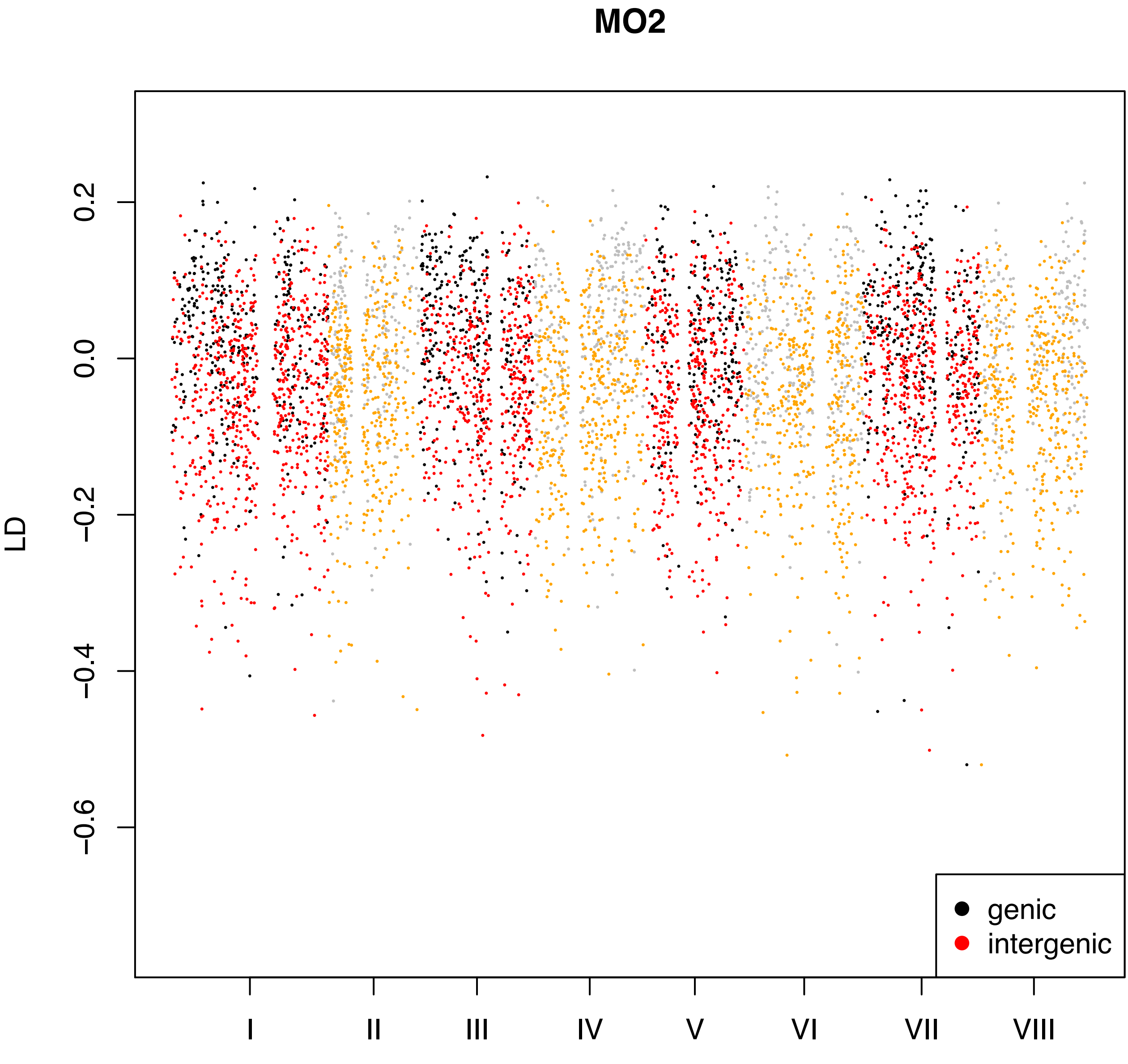
MO2
LD
0.2
0.0
−0.2
−0.4
−0.6
I
II
III
IV
V
VI
VII
VIII
genic
intergenic

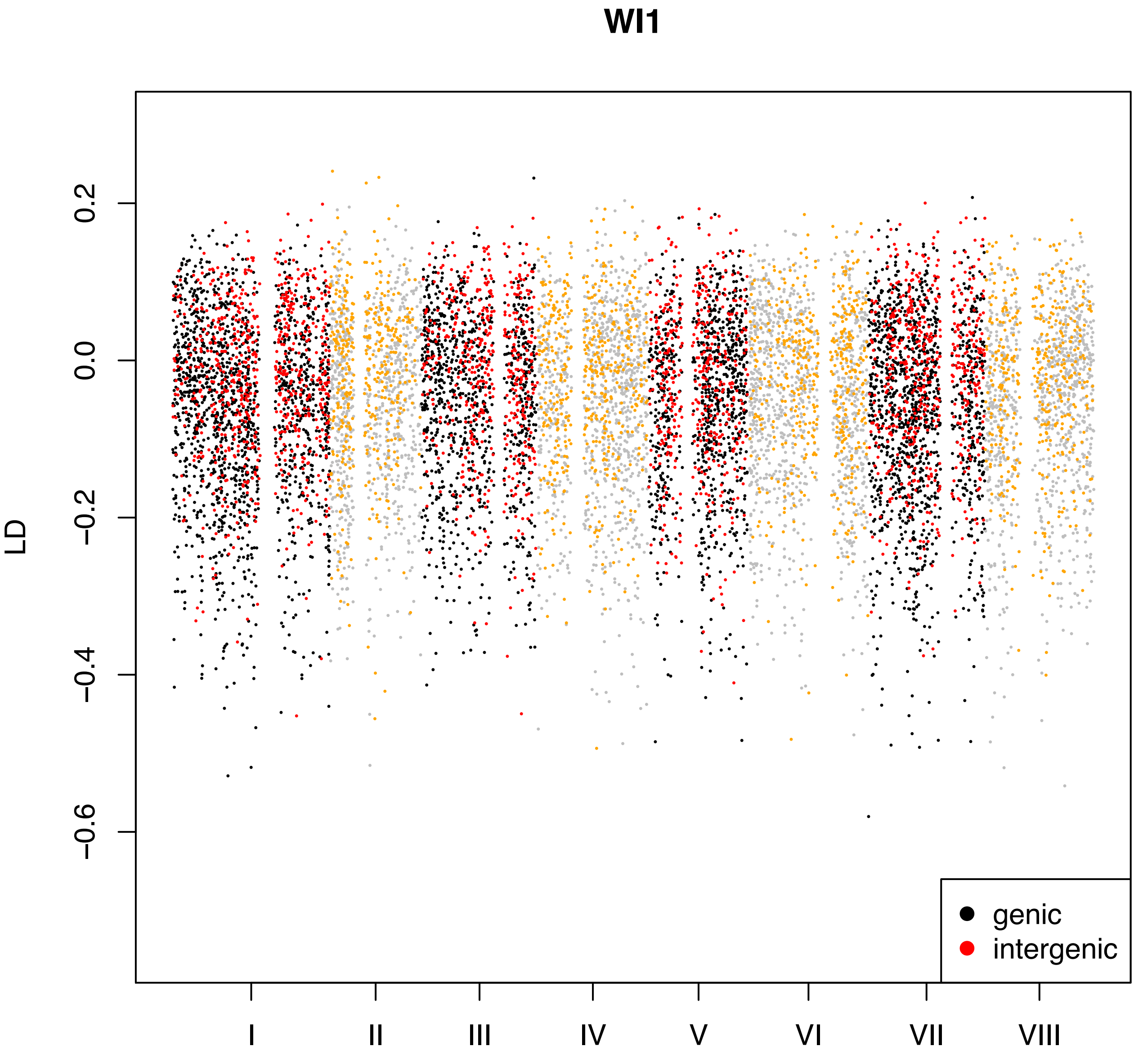
WI1
LD
0.2
0.0
−0.2
−0.4
−0.6
I
II
III
IV
V
VI
VII
VIII
genic
intergenic

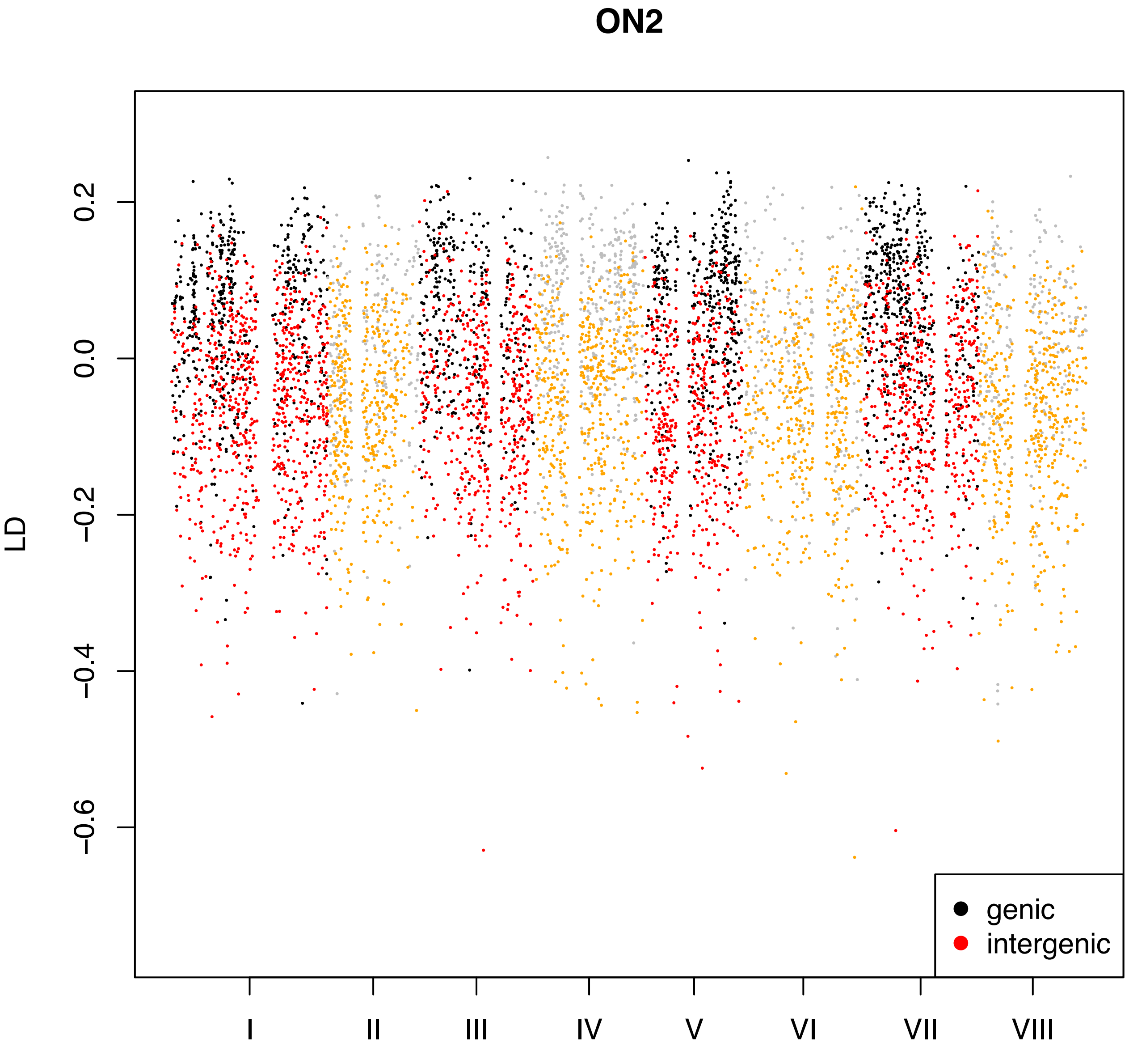
ON2
LD
0.2
0.0
-0.2
-0.4
-0.6
I
II
III
IV
V
VI
VII
VIII
genic
intergenic

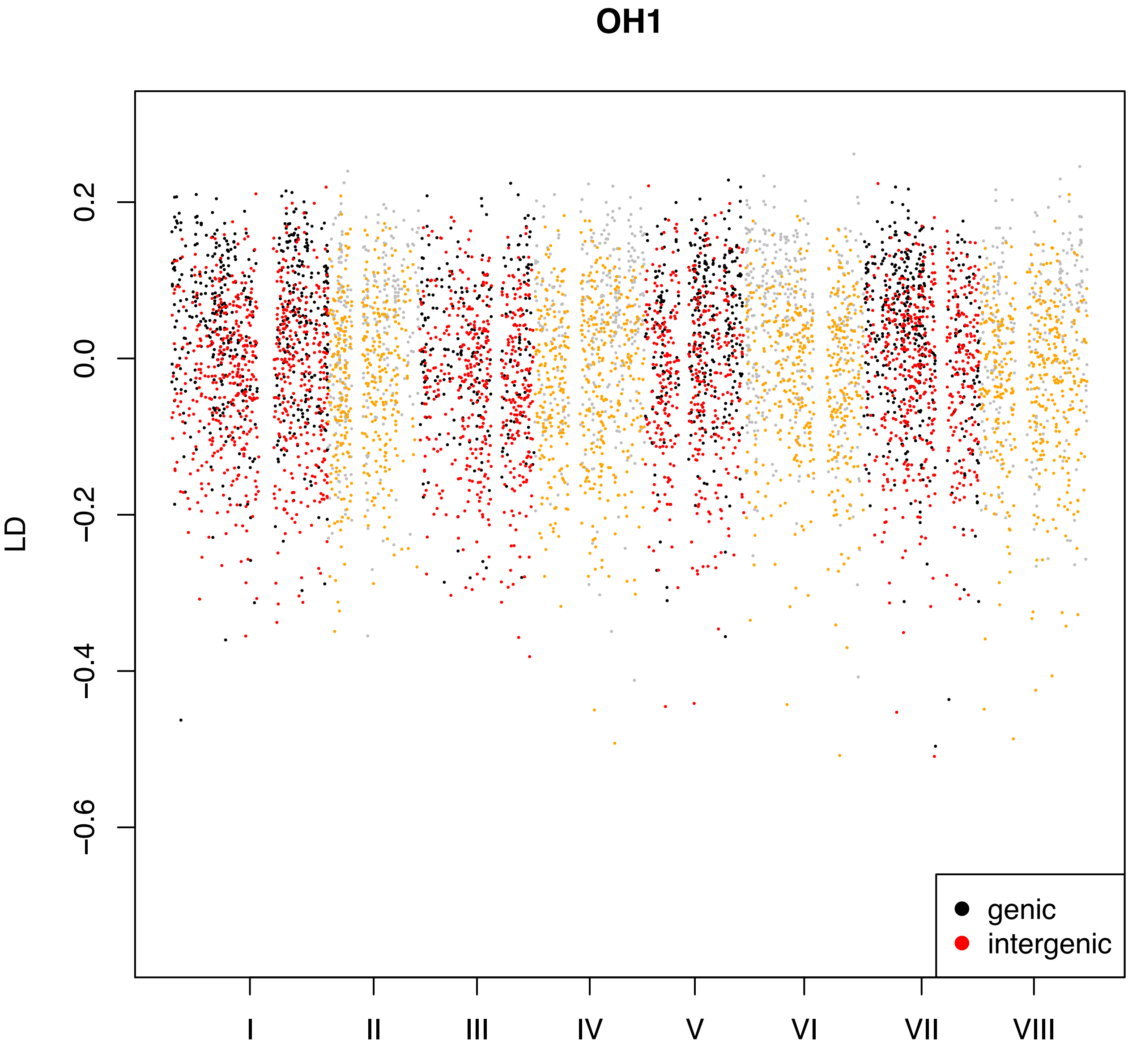
OH1
LD
0.2
0.0
−0.2
−0.4
−0.6
I
II
III
IV
V
VI
VII
VIII
genic
intergenic

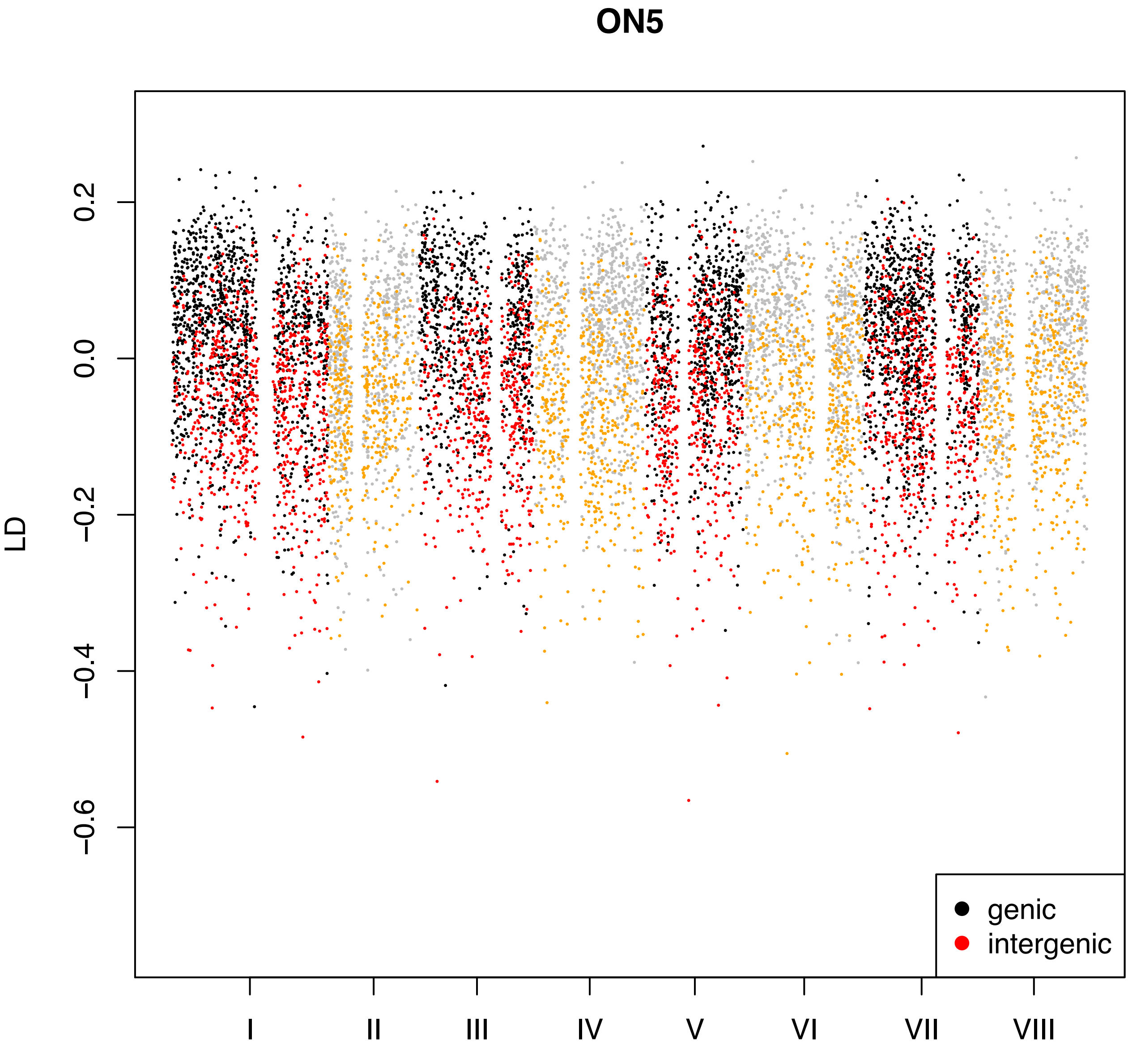

ON5
LD
0.2
0.0
-0.2
-0.4
-0.6
I
II
III
IV
V
VI
VII
VIII
genic
intergenic

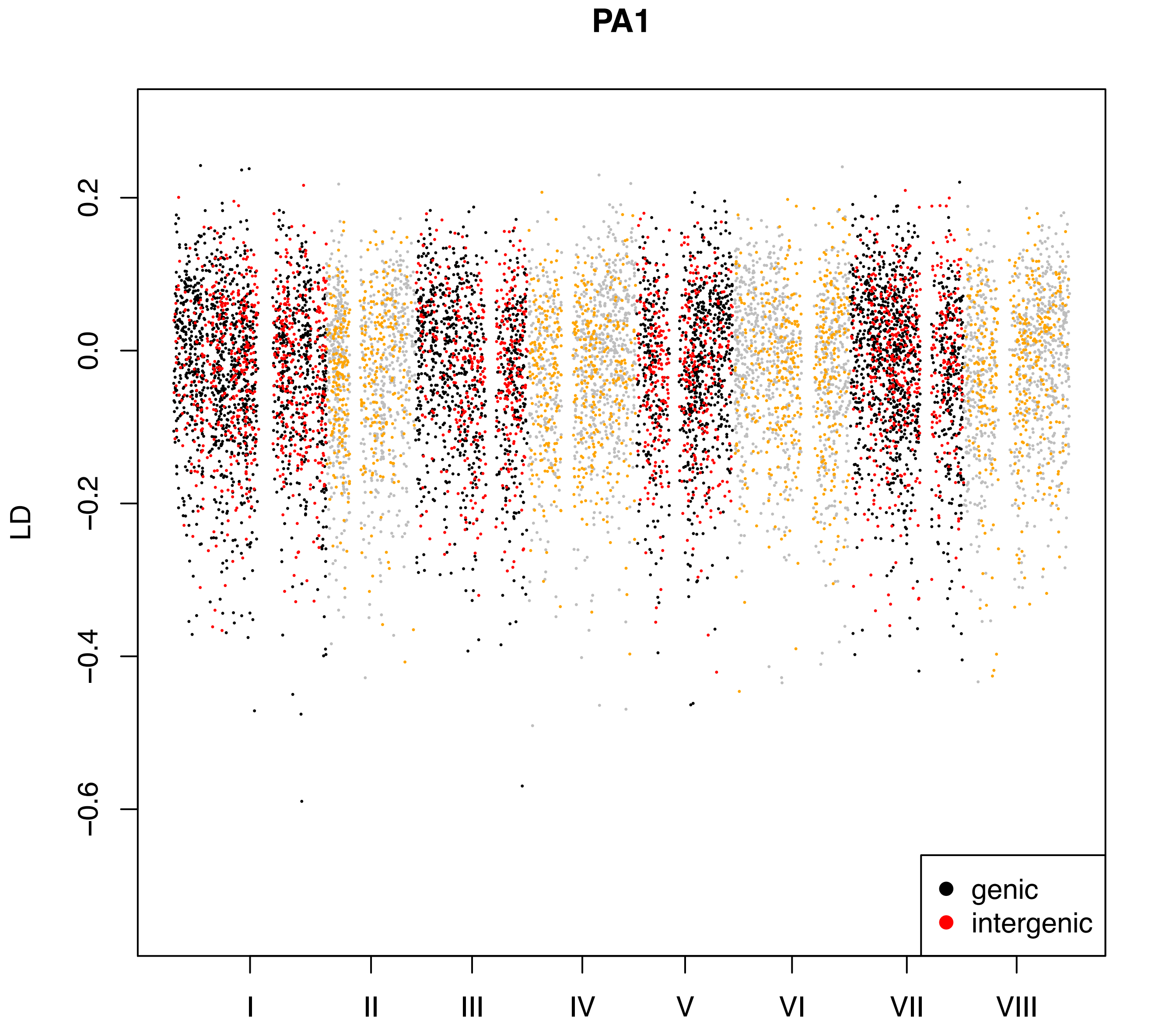

PA1
LD
0.2
0.0
−0.2
−0.4
−0.6
I
II
III
IV
V
VI
VII
VIII
genic
intergenic

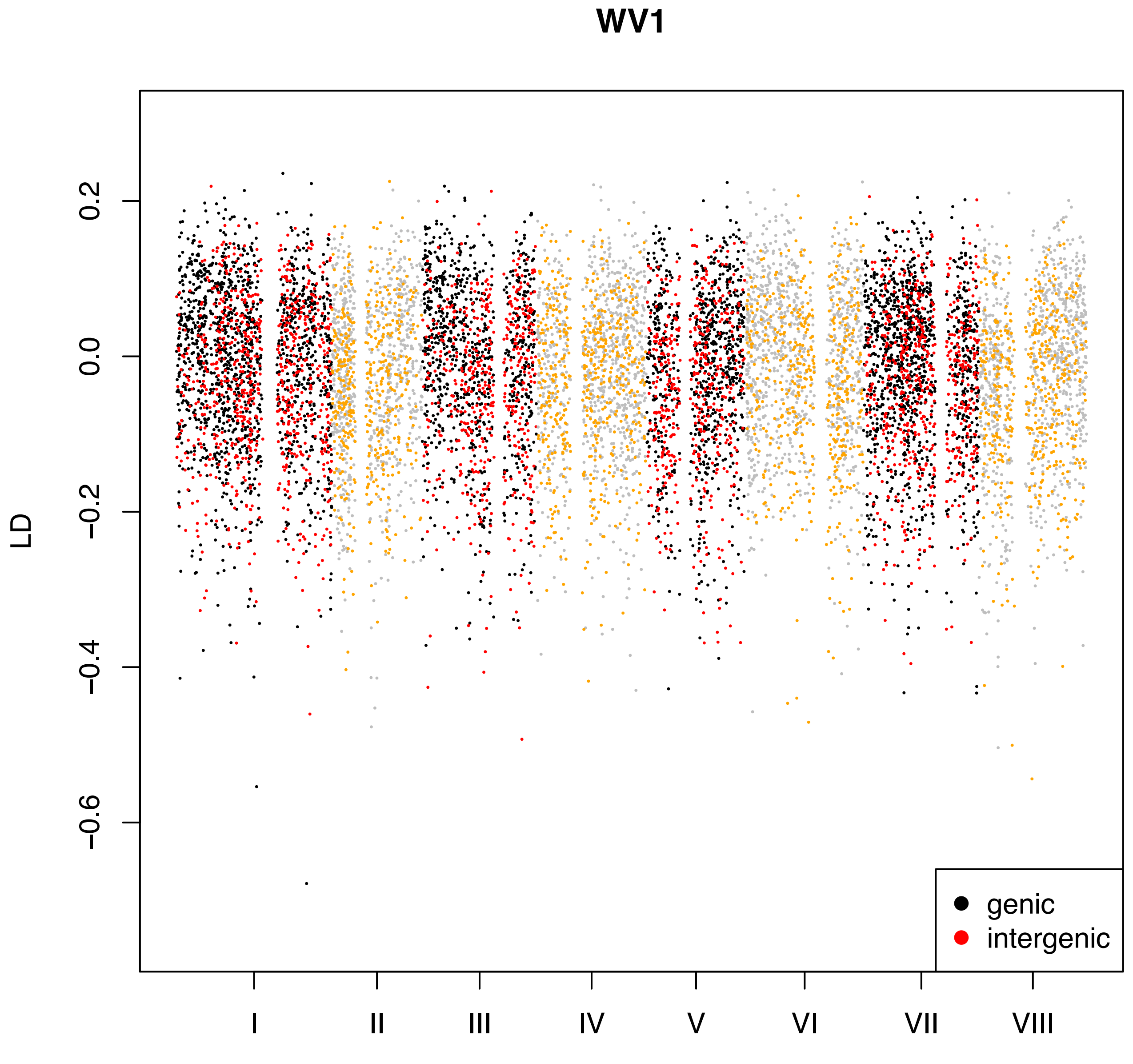

WV1
LD
0.2
0.0
−0.2
−0.4
−0.6
I
II
III
IV
V
VI
VII
VIII
genic
intergenic

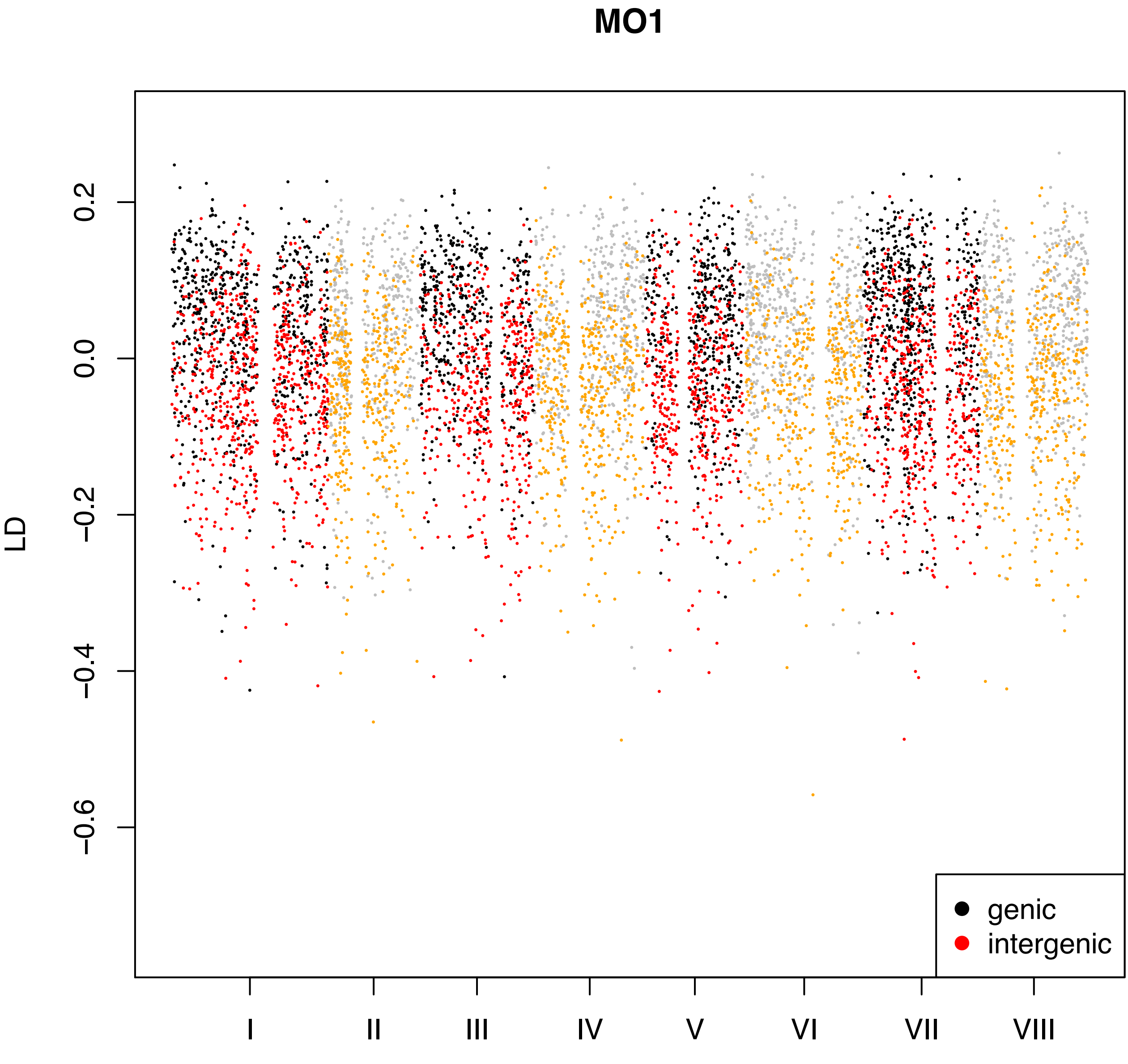

MO1
LD
0.2
0.0
−0.2
−0.4
−0.6
I
II
III
IV
V
VI
VII
VIII
genic
intergenic

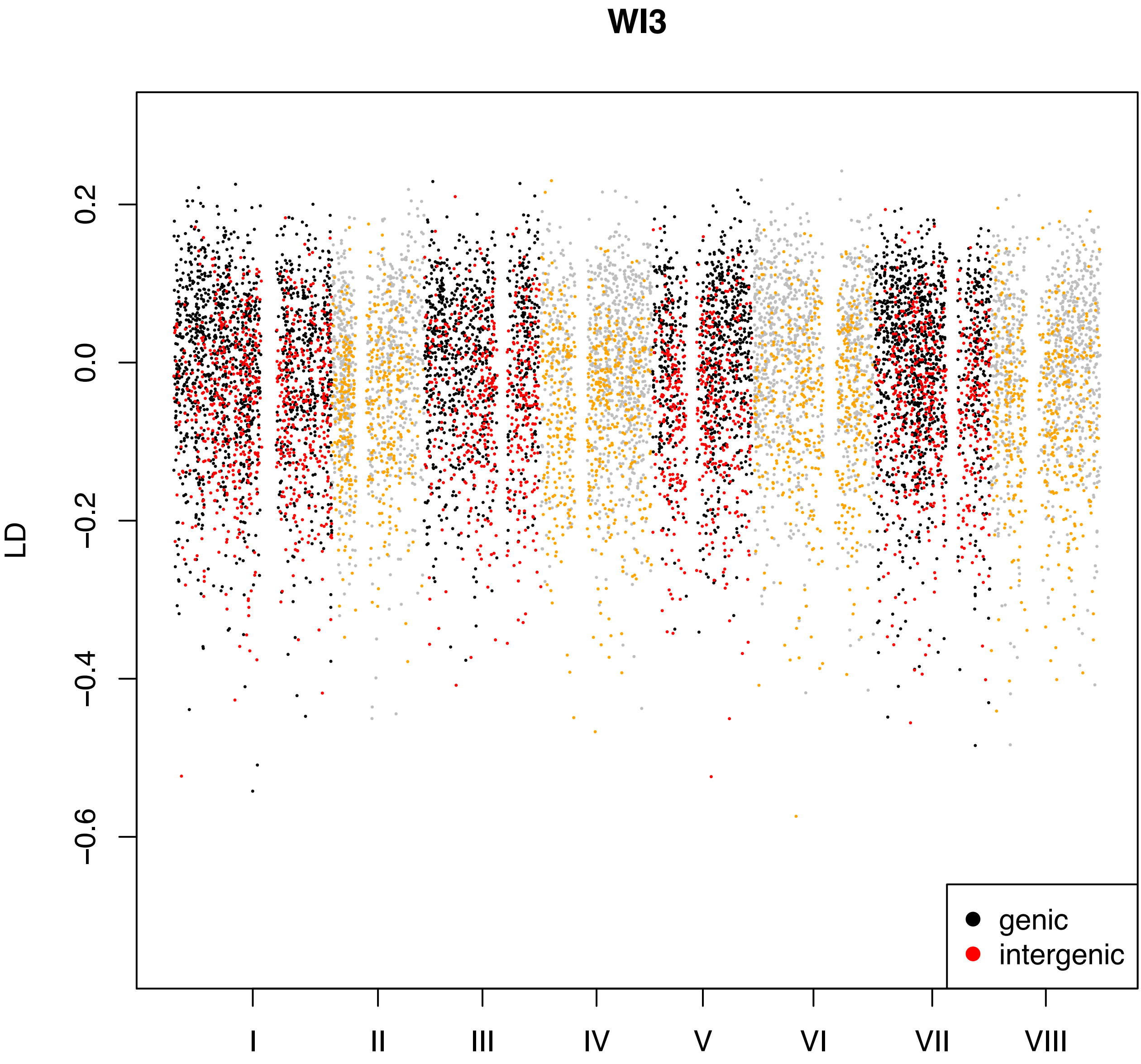
WI3
LD
0.2
0.0
−0.2
−0.4
−0.6
I
II
III
IV
V
VI
VII
VIII
genic
intergenic

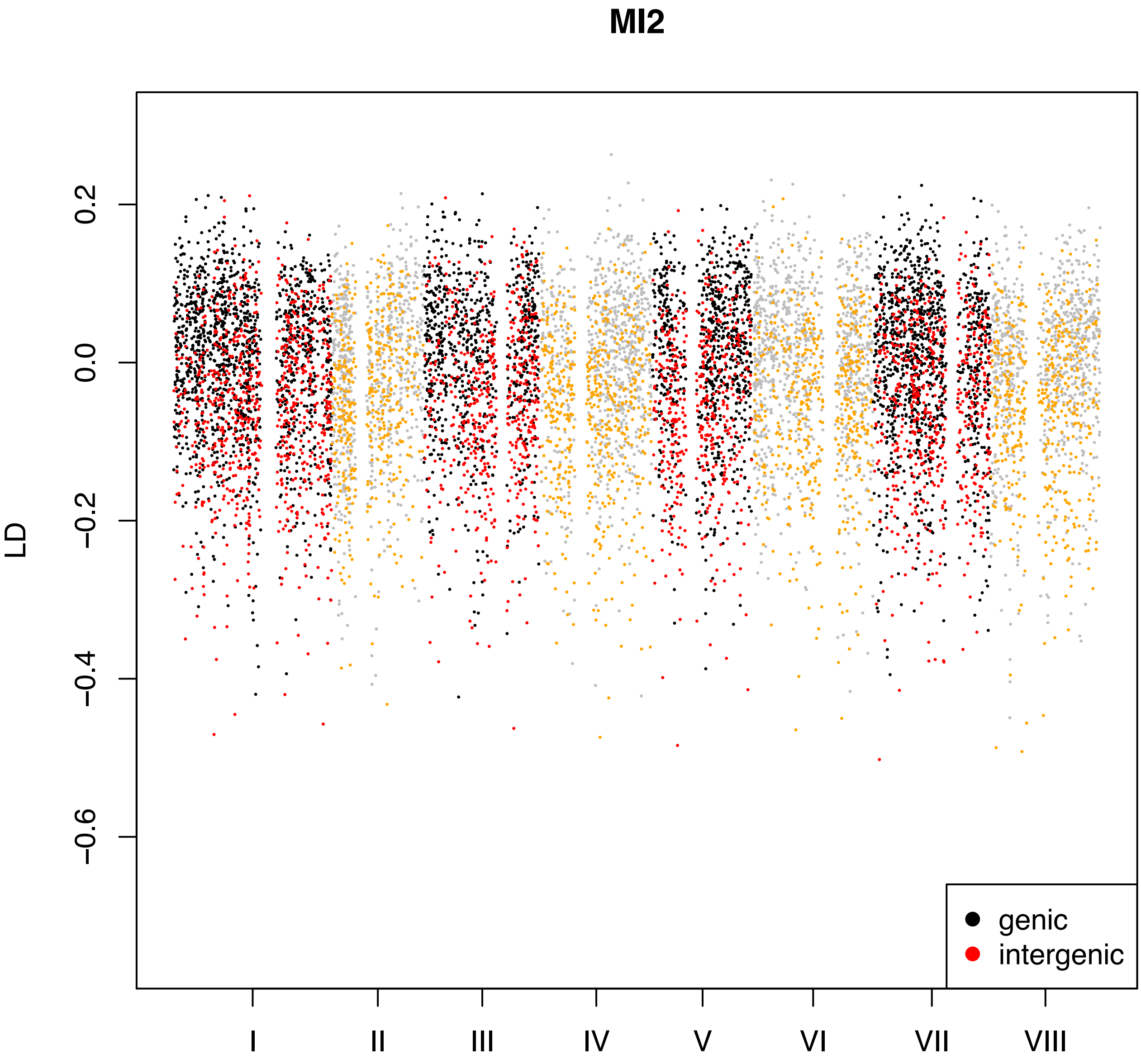

MI2
LD
0.2
0.0
−0.2
−0.4
−0.6
I
II
III
IV
V
VI
VII
VIII
genic
intergenic

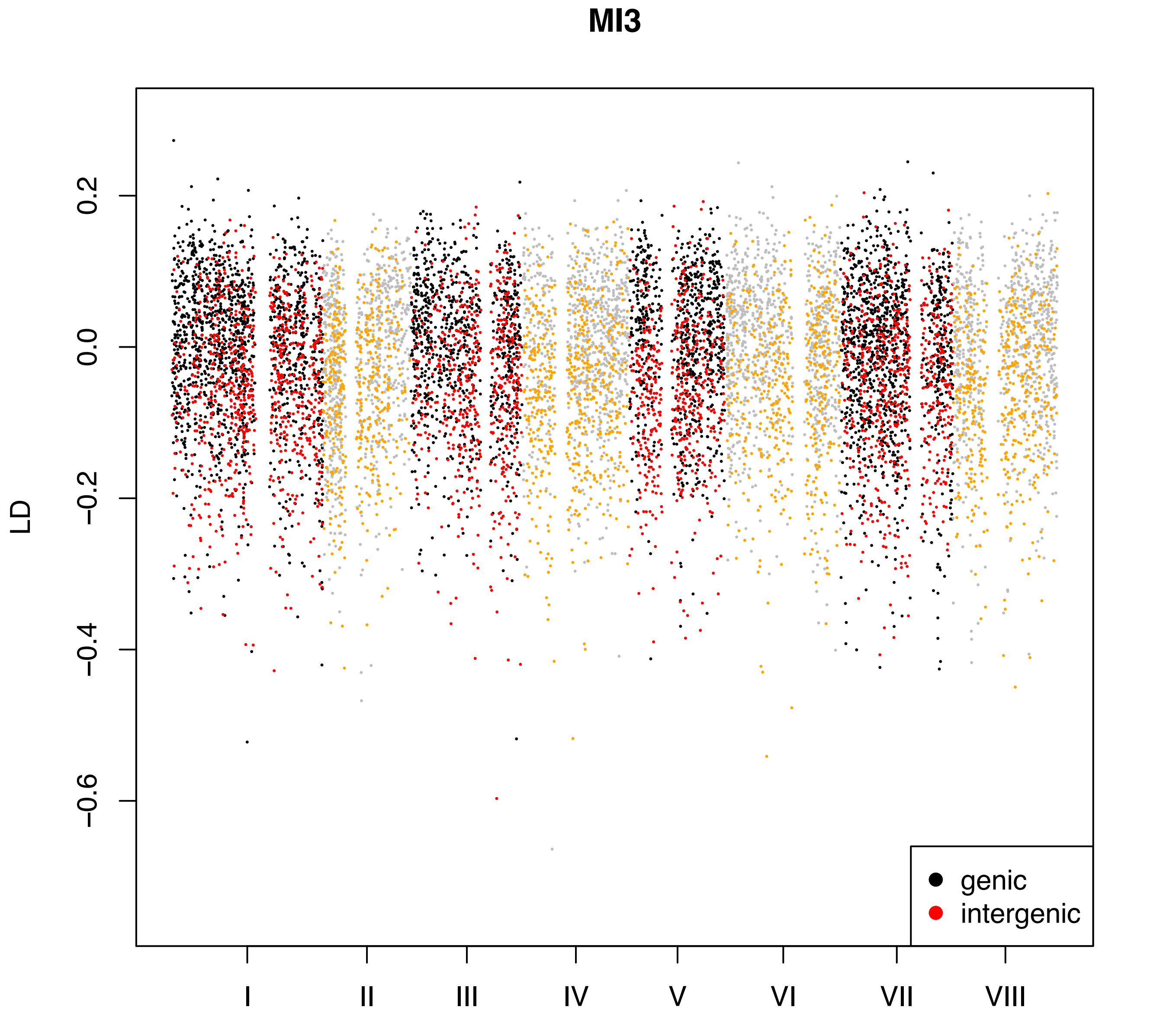

MI3
LD
0.2
0.0
−0.2
−0.4
−0.6
I
II
III
IV
V
VI
VII
VIII
genic
intergenic

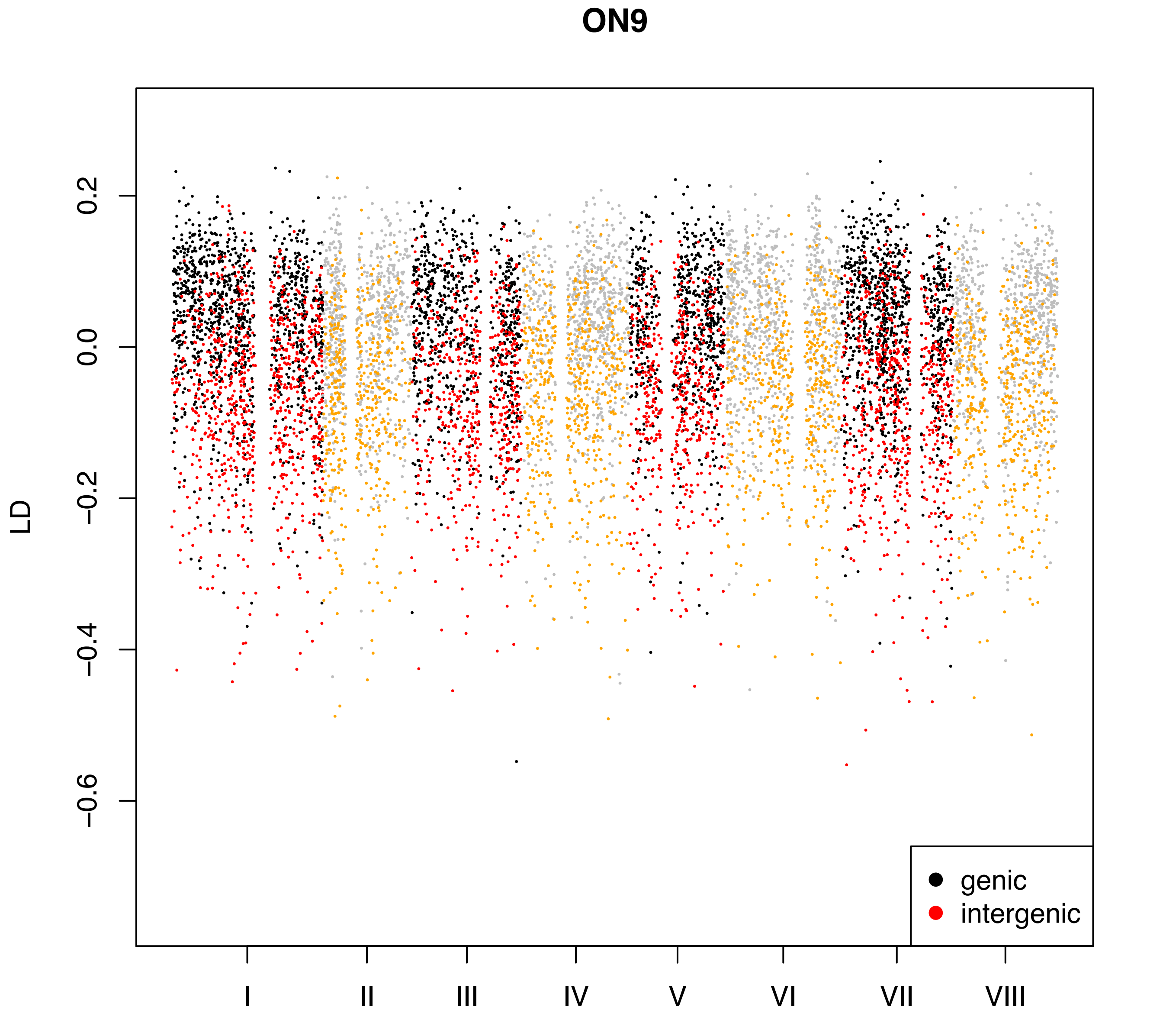

ON9
LD
0.2
0.0
-0.2
-0.4
-0.6
genic
intergenic
I
II
III
IV
V
VI
VII
VIII

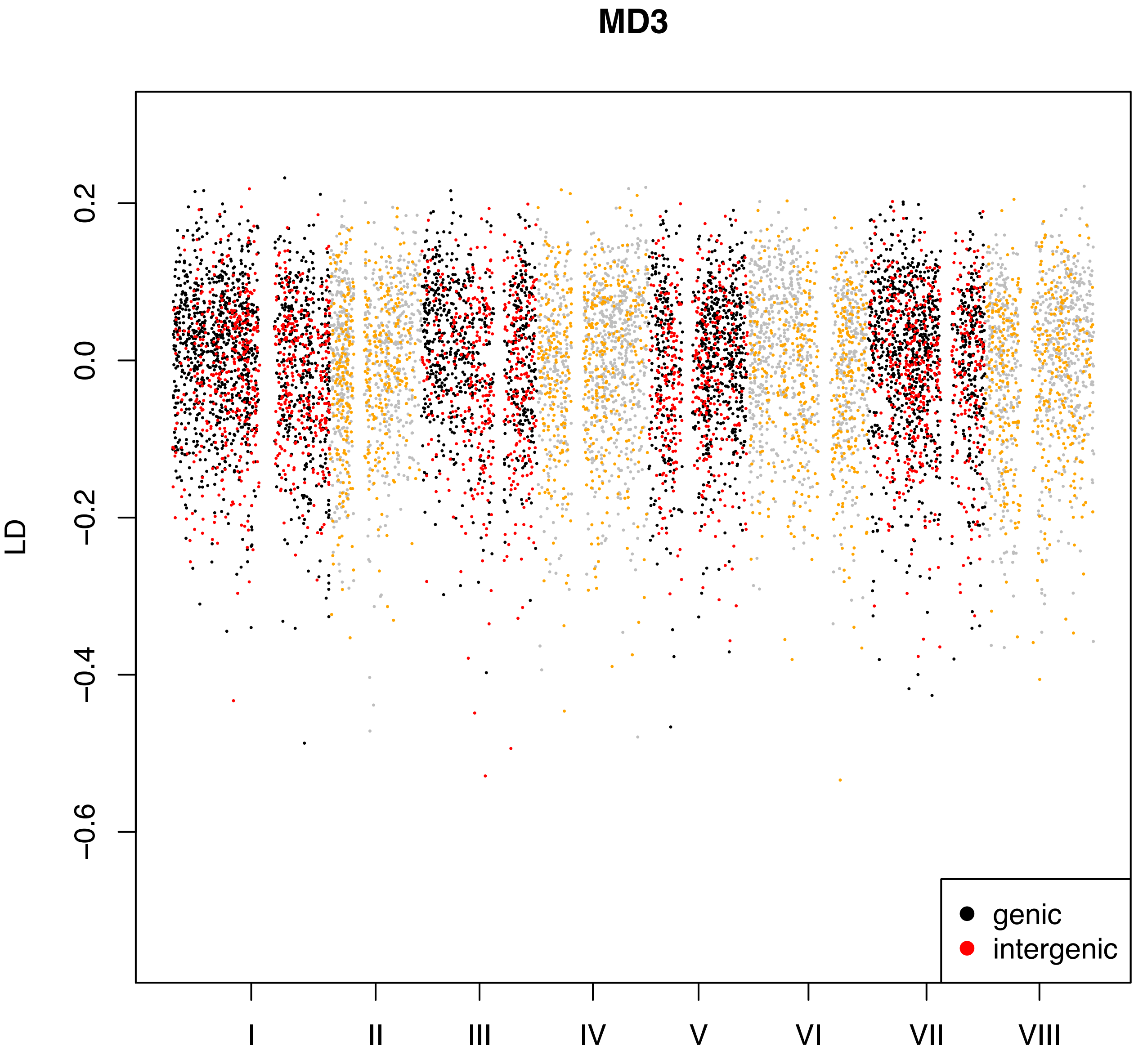
MD3
LD
0.2
0.0
−0.2
−0.4
−0.6
I
II
III
IV
V
VI
VII
VIII
genic
intergenic

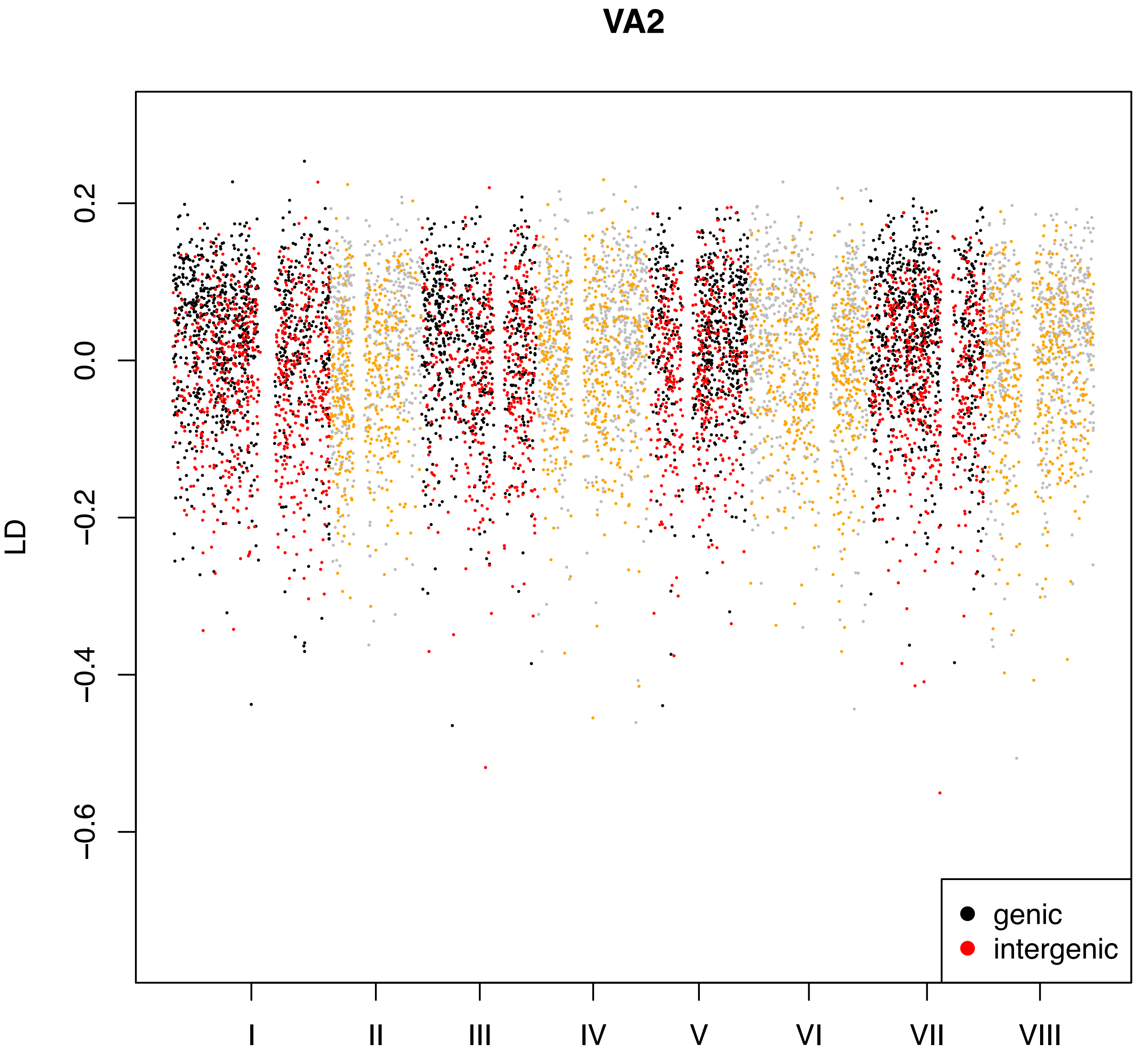

VA2
LD
0.2
0.0
-0.2
-0.4
-0.6
I
II
III
IV
V
VI
VII
VIII
genic
intergenic

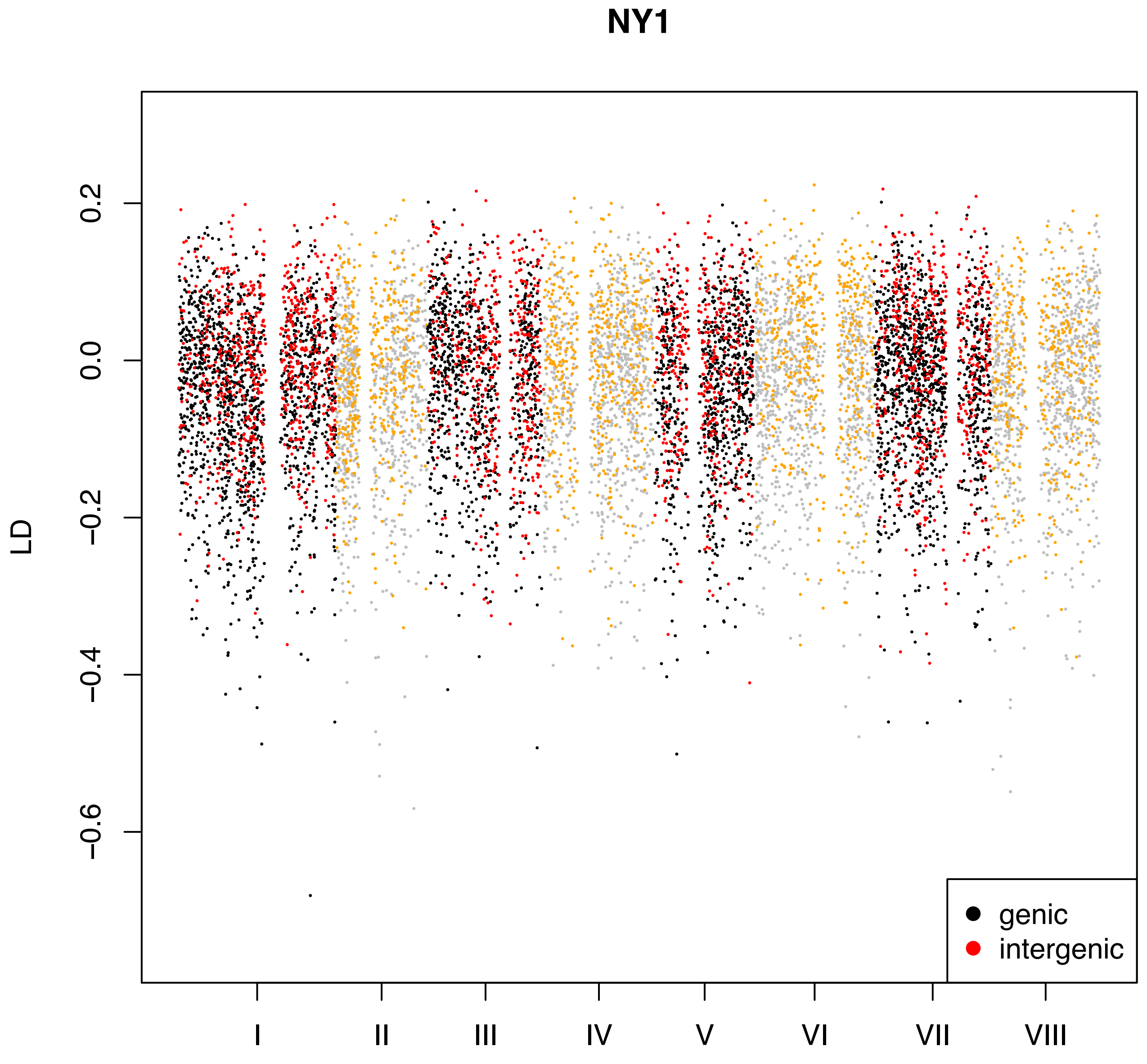

NY1
LD
0.2
0.0
−0.2
−0.4
−0.6
I
II
III
IV
V
VI
VII
VIII
genic
intergenic

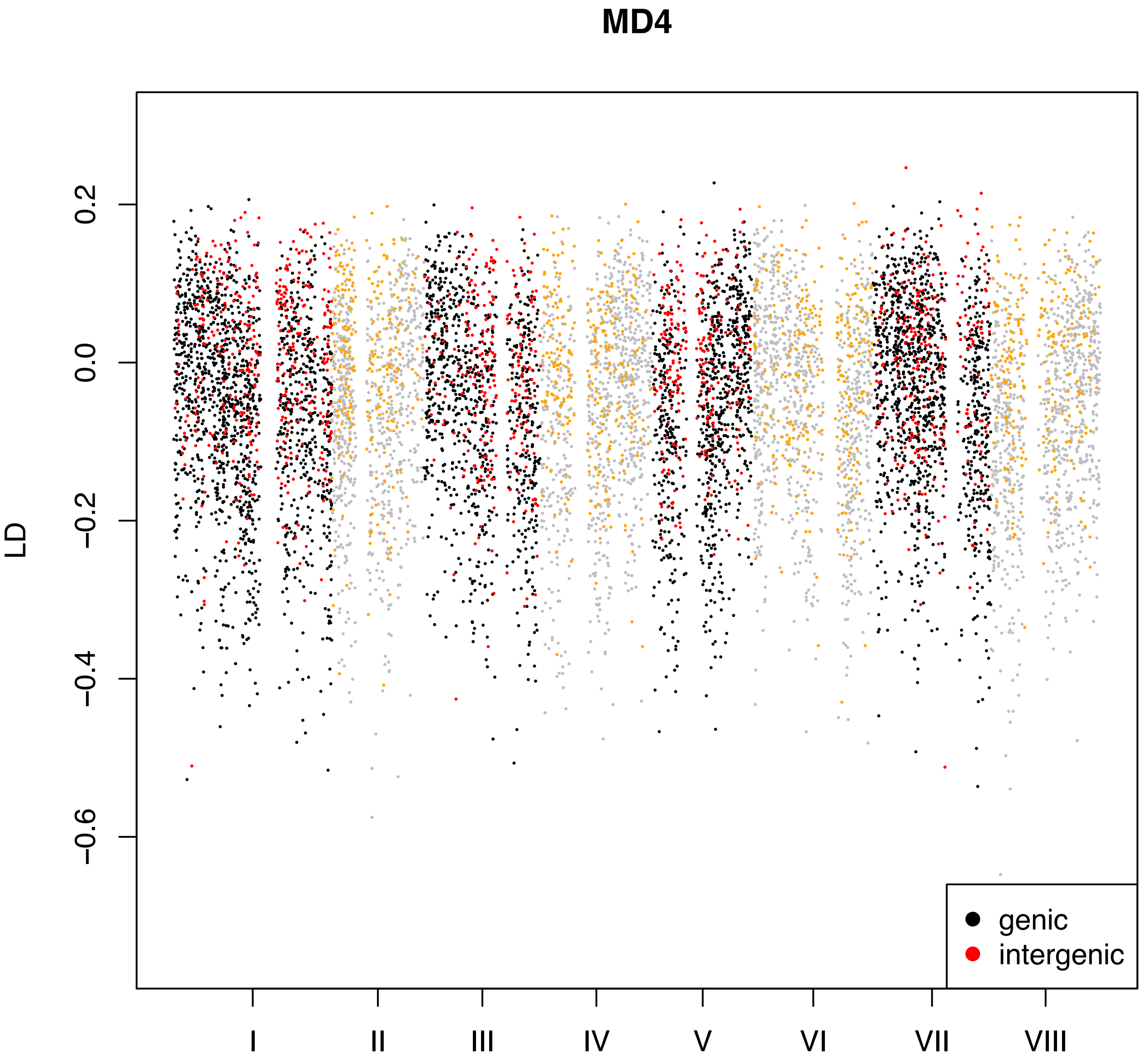

MD4
LD
0.2
0.0
−0.2
−0.4
−0.6
I
II
III
IV
V
VI
VII
VIII
genic
intergenic

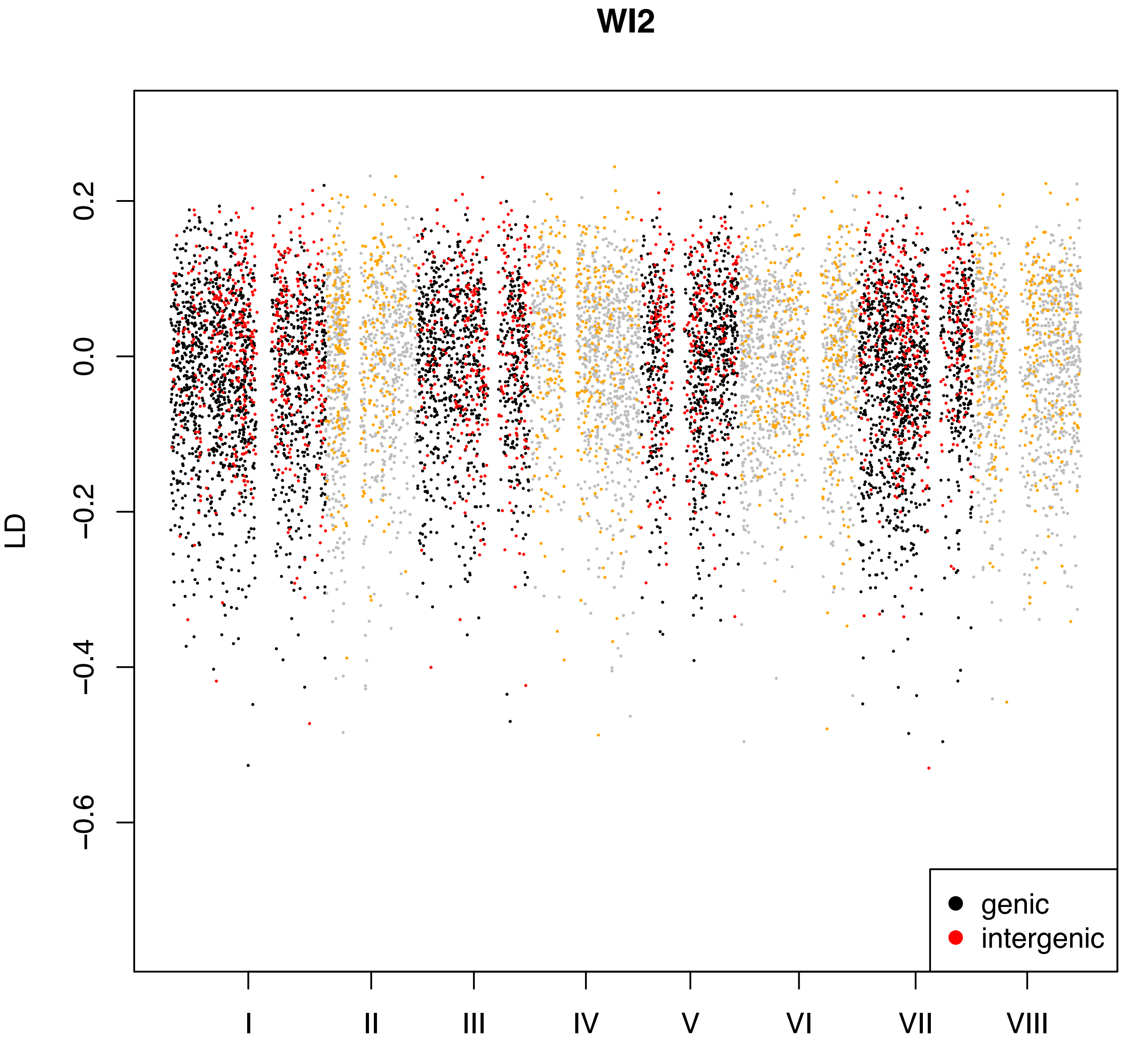

WI2
LD
0.2
0.0
−0.2
−0.4
−0.6
I
II
III
IV
V
VI
VII
VIII
genic
intergenic

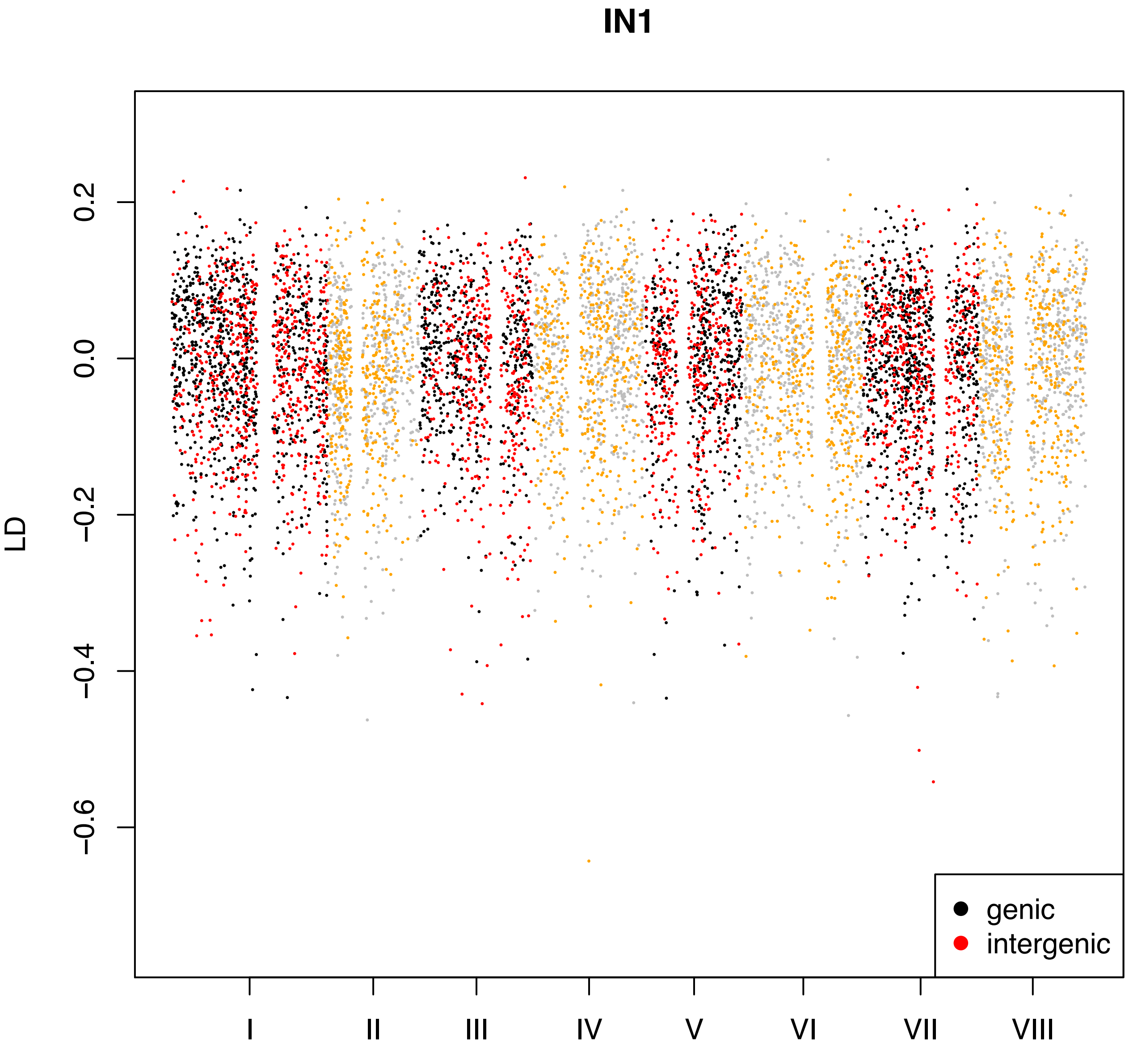

IN1
LD
0.2
0.0
−0.2
−0.4
−0.6
I
II
III
IV
V
VI
VII
VIII
genic
intergenic

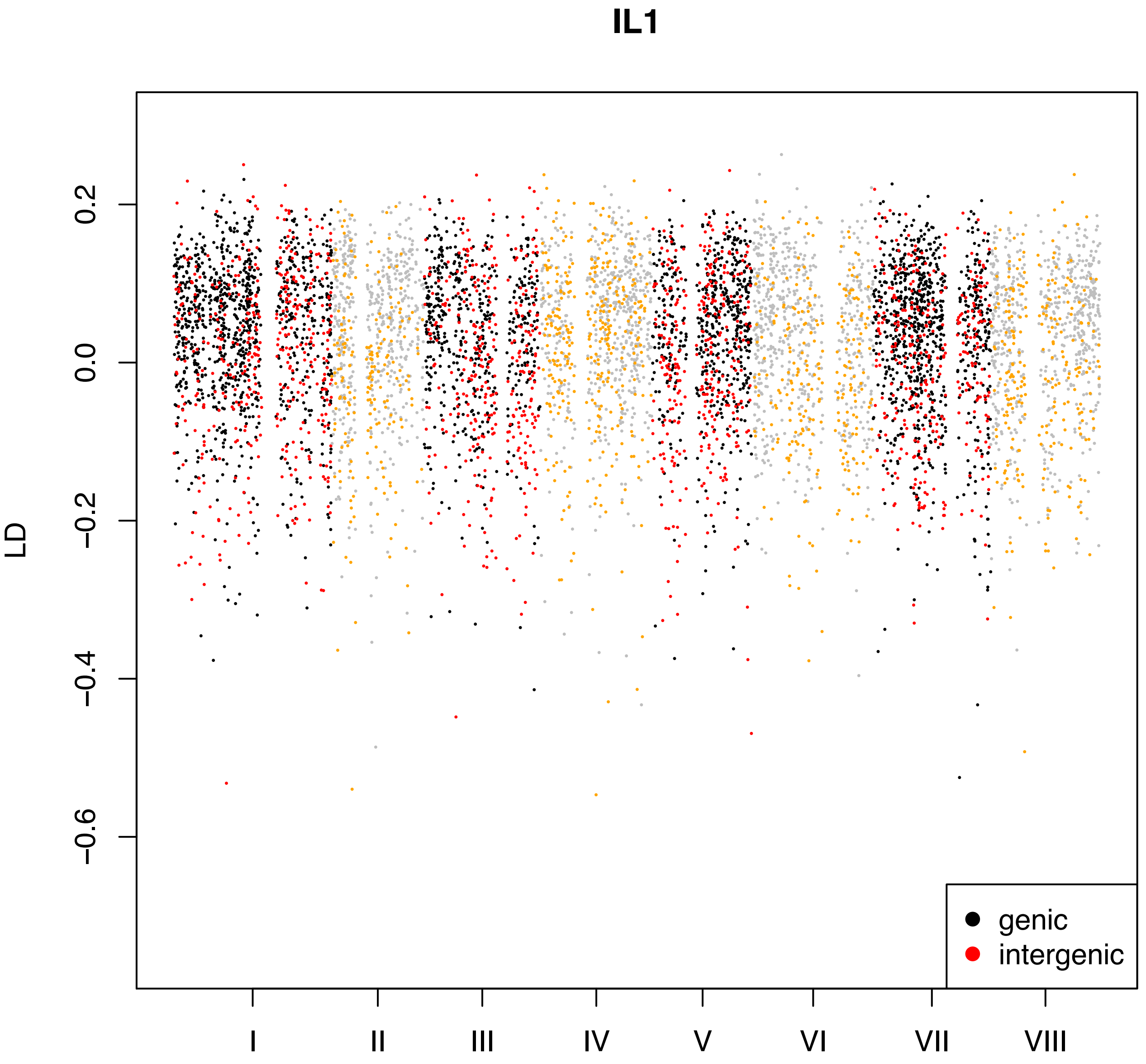
IL1
LD
0.2
0.0
−0.2
−0.4
−0.6
I
II
III
IV
V
VI
VII
VIII
genic
intergenic

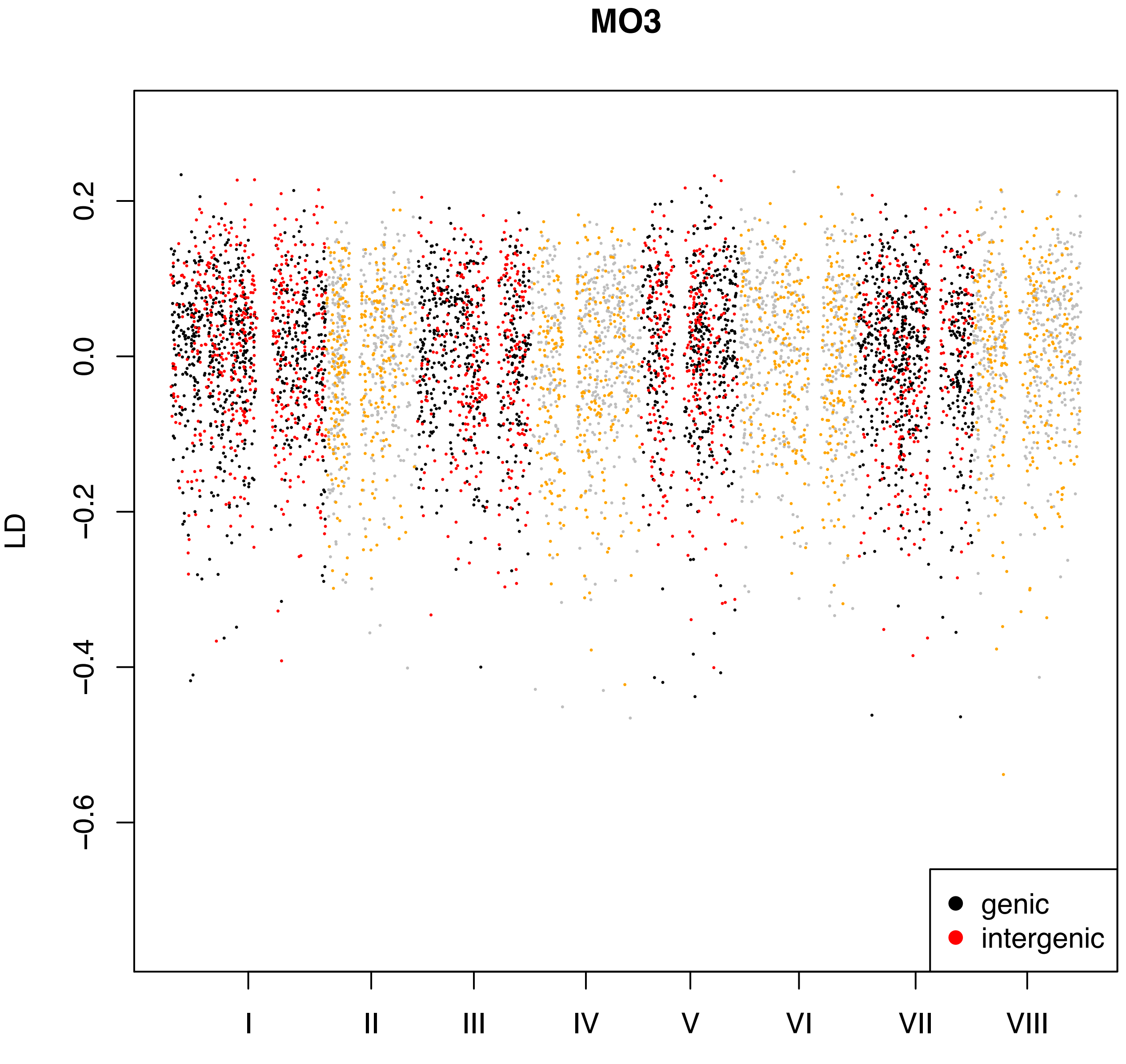
MO3
LD
0.2
0.0
−0.2
−0.4
−0.6
I
II
III
IV
V
VI
VII
VIII
genic
intergenic
