# Supplementary figures and images for: Drivers of linkage disequilibrium across a species’ geographic range
Source: PLoS Genet. 2021 Mar 26;17(3):e1009477. doi: 10.1371/journal.pgen.1009477 (PMC8026057; doi:10.1371/journal.pgen.1009477)

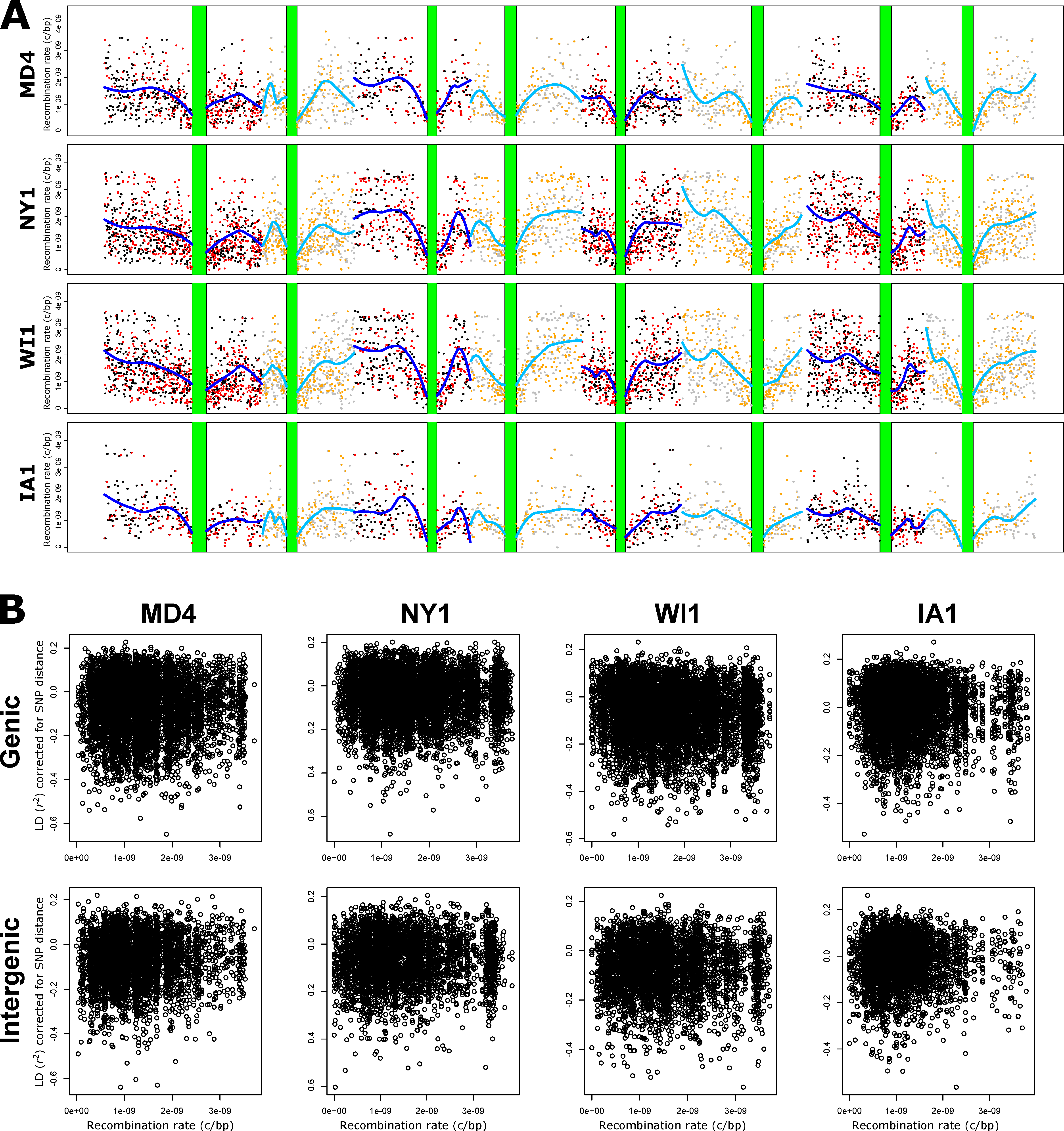

Supplement: S5 Fig — A–recombination (c/bp) estimated for genic (black/grey) and intergenic (red/orange) regions used in this study with the blue lines indicating the smoothed average recombination across each genome. The eight scaffolds of the A. lyrata genome are indicated with alternating shading (black/red vs. grey/orange). Green bars indicate centromeric regions. B–relationship between recombination rate and LD estimated from pool-seq data for genic (top) or intergenic (bottom) regions. (TIFF) [file pgen.1009477.s005.tiff]

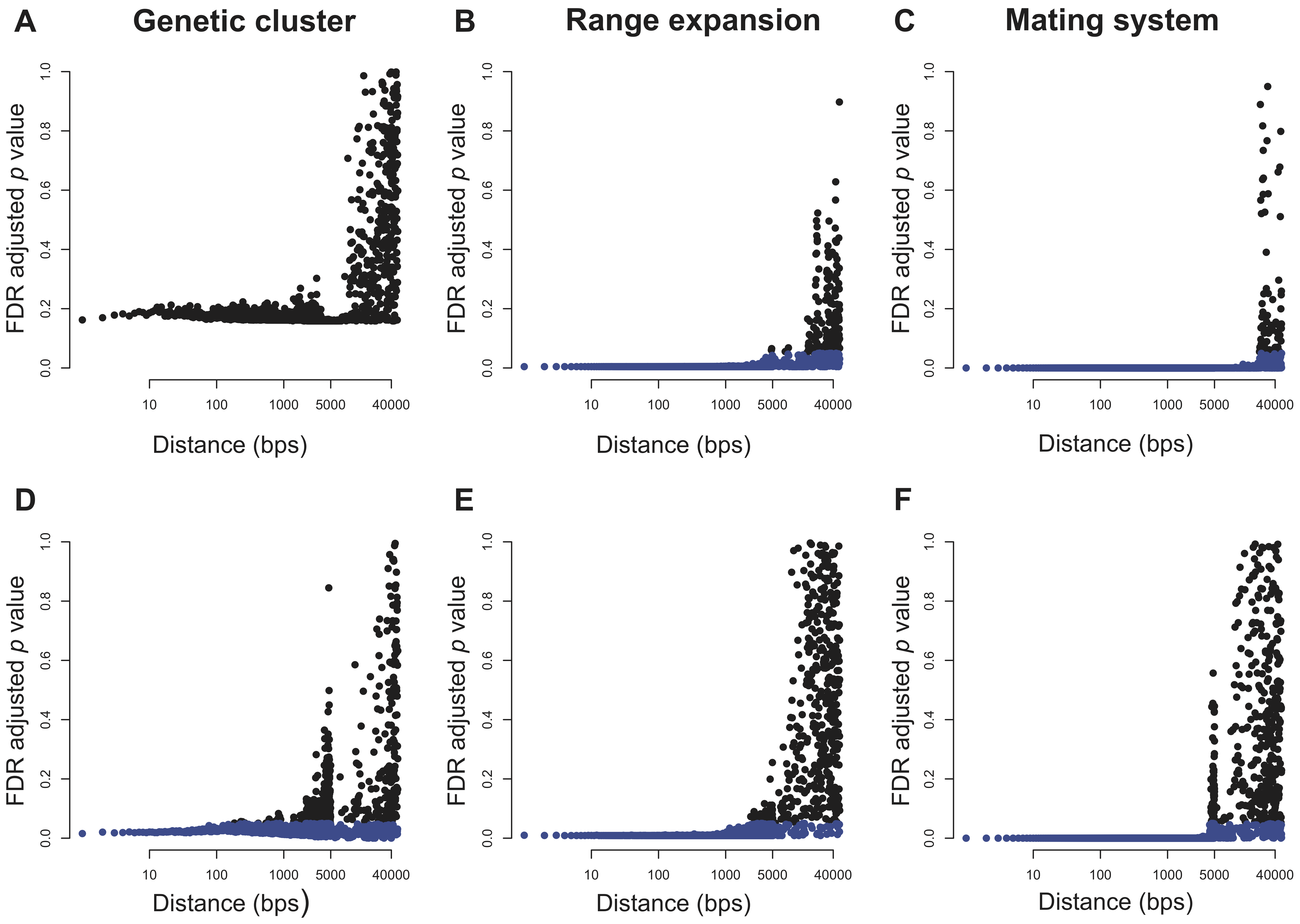

Supplement: S7 Fig — The level of significance (p values) adjusted for multiple testing with a false discovery rate (FDR) for the different fixed effects of linear mixed-effects models for genic (A-C) and intergenic (D-F) regions. Response variable was the correlation of zygosity (Δ) scaled by the level of genome-wide heterozygosity (θ) for each bp distance for 1–5,000 bps and in bins of 100 bps between 5'001–50'000 bps. P values that were significant (i.e. p < 0.05) after an FDR are highlighted in blue. (TIFF) [file pgen.1009477.s007.tiff]
