# Supplementary material for: Drivers of linkage disequilibrium across a species’ geographic range
Source: PLoS Genet. 2021 Mar 26;17(3):e1009477. doi: 10.1371/journal.pgen.1009477 (PMC8026057; doi:10.1371/journal.pgen.1009477)
Supplement: S1 Text — (DOCX) [file pgen.1009477.s011.docx]

**S1 Text for “Drivers of linkage disequilibrium across a species’ geographic range”**

Six populations were old, i.e. established before the retreat of the glaciers following the last glaciation period: three located in Missouri, two along the Mississippi River in Iowa and one in New Jersey (S1 Table). We tested for an effect of old populations on LD in response to the distance from the respective core of each glacial refugia using the genome-wide averaged LD estimates. We employed a linear model separately for genic and intergenic regions. The model included the status of a population (old *vs*. postglacial) and the expansion distance as fixed effects. While both models were significant (genic: *R*^2^=0.359, *F*_2,49_=13.7, *p*<0.001; intergenic: *R*^2^=0.306, *F*_2,49_=10.8, *p*<0.001), old populations explained no variation (genic: *t*=0.38, *p*=0.705; intergenic: *t*=0.51, *p*=0.614). This suggests that old populations show a similar level of LD as populations along the expansion at a similar distance (Figure A). We further compared the slopes of the relationships between LD and expansion distance as well as LD and mutational load between the datasets comprising all 52 populations or the reduced dataset without the six old populations for both genic and intergenic regions. None of the slopes did significantly differ (expansion distance genic regions: *p*=0.534; expansion distance intergenic regions: *p*=0.620; mutational load genic regions: *p*=0.285; mutational load intergenic regions: *p*=0.400).

*Figure A: Relationship between genome-wide linkage disequilibrium (LD, distance-corrected r^2^ between pairs of SNPs) for genic (left) and intergenic (right) regions and expansion distance or distance from core for rear-edge populations. Each symbol represents the average estimate of a population. The symbol type reflects the genetic clusters (dot for west, triangle for east) and the symbol colour the mating system (black for predominant outcrossing, white for predominant selfing, gray for mixed mating). Evolutionary old populations are highlighted in red.*

We further repeated the genome-wide analyses as outlined in the main text but removing the six old populations. Removing the old populations changed little, i.e. genome-wide LD was strongly associated with expansion distance and mating system for both genic and intergenic portions of the genome (Tables B and C; Fig A). LD increased with range expansion and with increasing *F*_IS_, i.e., with the switch to an inbreeding/selfing reproductive mode. Moreover, LD declined with increasing average distance from the neighbouring genes, indicating that higher gene density is associated with higher LD.

Table B: *Results of the linear mixed-effects model with genome-wide linkage disequilibrium (LD, distance-corrected r^2^ between pairs of SNPs) of genic regions as the dependent variable. Only results for fixed effects are shown (for all: df = 1, 41’856’655).*

| **Fixed effect** | ***β* ± SE** | **χ^2^** | ***P*** |
| --- | --- | --- | --- |
| Genetic cluster (east) | 0.00607   ±0.00456 | 1.770 | 0.1834 |
| Log_10_ expansion distance | 0.04323   ±0.01002 | 18.625 | <0.0001 |
| Mating system (*F*_IS_) | 0.02880   ±0.00895 | 10.354 | 0.0013 |
| Log_10_ distance to adjacent genes | -0.01324 ±0.00105 | 158.770 | <0.0001 |

Table C: *Results of the linear mixed-effects model with genome-wide linkage disequilibrium (LD, distance-corrected r^2^ between pairs of SNPs) of intergenic regions as the dependent variable. Only results for fixed effects are shown (for all: df = 1, 17’009’753).*

| **Fixed Effect** | ***β* ± SE** | **χ^2^** | ***P*** |
| --- | --- | --- | --- |
| Genetic cluster (east) | 0.00861   ±0.00379 | 5.166 | 0.0230 |
| Log_10_ expansion distance | 0.03132   ±0.00832 | 14.172 | 0.0002 |
| Mating system (*F*_IS_) | 0.02766   ±0.00744 | 13.842 | 0.0002 |
| Log_10_ size intergenic region | -0.01413 ±0.00170 | 69.466 | <0.0001 |

Finally, we repeated the gene-by-gene analysis as outlined in the main text but omitting the six old populations. Applying the same models and following a FDR correction, we identified a total of 440 genes that showed a significant and higher than expected association with range expansion and 22 genes that showed a significant and higher than expected association with mating system. For range expansion, 239 outlier genes overlapped with the dataset using all populations, representing 88.1% of all genes in that dataset. For mating system, 16 genes overlapped, representing 69.6% of all genes in that dataset.
