# Supplementary material for: Drivers of linkage disequilibrium across a species’ geographic range
Source: PLoS Genet. 2021 Mar 26;17(3):e1009477. doi: 10.1371/journal.pgen.1009477 (PMC8026057; doi:10.1371/journal.pgen.1009477)
Supplement: Attachment — Submitted filename: Response.pdf [file pgen.1009477.s012.pdf]

Revised manuscript PGENETICS-D-20-00232

Dear editors

We would like to thank the referees and Dr. Bomblies for the helpful comments on our manuscript. Below we copied the comments in blue and write how we addressed them in our manuscript in black.

Comments by Dr. Bomblies:

1. All three [reviewers] found the topic interesting, yet two of them voiced strong criticism on the fact that the manuscript is lacking a theoretical foundation that would help address better the causes and consequences of the correlations you report.

We revised the theory section of the Introduction and present now in more depth the predictions on the role of expansion/drift, selfing and selection on linkage disequilibrium, LD (lines 66-78, 87-95, 102-109, also see response to comments 2.1 by reviewer 2, and 3.2 by reviewer 3). Furthermore, we now discuss our results in the light of the Hill-Robertson effect (lines 517-522, also see response to comment 3.0 by reviewer 3).

2. It is difficult to extrapolate the relative importance of selfing and range expansion with linear models, since linear models assume independence among the observation, an assumption that is presumably violated on a genome.

We used hierarchical mixed-effects models to account for this sort of structure in the dataset. It is correct that replicate estimates of a genome of an individual or a population are not independent. This is accounted for in the random part of the model and by defining the hierarchy – estimates of genomes are nested within scaffolds and within populations (e.g., see Runcie & Crawford 2019 PLoS Genetics and references therein; lines 279-281).

Note that our revised manuscript includes two major new analyses: i) We estimated levels of background recombination by a novel machine-based learning software that became accessible after our initial submission. We show that our estimates of LD and background recombination were not tightly correlated (lines 361-364, Figure S3; also see response to comment 3.1 by reviewer 3). ii) We annotated the potential effects of our SNPs using SIFT and repeated our analysis including this annotation. While the overall analyses draws a consistent picture with the results of our initial submission, we now show that increased LD due to range expansion or selfing includes to a similar fraction presumably neutral SNPs and presumably deleterious SNPs (novel Figure 2; also see response to comment 1.3 by reviewer 1).

3. Should the authors be willing to revise their work and perform additional analyses, I believe it would be important to critically examine the methods. I often had to guess how the analysis was done. It is surely possible to be more accurate there.

We clarified our methods section (line 165 onwards; also see response to comment 3.1 by reviewer 3) and now include new analyses as described in the response to comment 2.

4. I would also like to suggest to consider the view defended by Simons YB, Sella G. (The impact of recent population history on the deleterious mutation load in humans and close evolutionary

relatives. 2016. Current Opinion in Genetics & Development 41:150–158.), which offers an alternative and possibly more powerful way of estimating the individual deleterious load. This would allow an independent analysis of the link between population size, LD, and the mutational load.

By annotating SNPs with SIFT and repeating our mixed-effects model, our revised manuscript directly assesses the impact of LD on deleterious sites (see response to comment 2).

Comments by Reviewer 1:

1.1 Arabidopsis lyrata has two subspecies: ssp. lyrata and ssp. petraea. The author only analyzed the North American subspecies ssp. lyrata. The two subspecies diverged about ~250 Kya (Mattila et al. 2015 ME). I believe they still compatible and possible gene flow may occur between the two subspecies, which may change the genetic patterns of LD and mutation load. I want to know what the authors think about this.

The reviewer is correct about this piece of natural history. As it is common in plants, individuals of both subspecies can be crossed in the lab and produce fertile offspring. However, the two subspecies are geographically completely isolated by oceans and other natural barriers and there is no evidence for gene flow or admixture between them (e.g. Hohmann et al. 2014 BMC Evol Biol). We now state the latter explicitly in our manuscript (lines 113-116).

1.2 The authors wanted to study the effects of expansion on LD, but used a cutoff of MAF > 0.15. We know that expansion will lead to an excess of low frequency mutations (i.e. the SFS shift to the left). Did the authors excluded most of the expansion effects in the SNPs filtering step?

The reviewer is correct in that there should be a shift toward an increase in rare (but not the very rarest) deleterious mutations under drift (Peischl & Excoffier 2015 Mol Ecol), but the frequency of rare neutral mutations should actually decline. Given that the ends of SFS are particularly prone to be affected by drift, focusing on mid-frequency SNPs seems to be conservtive. Also, in the paper describing the original method of estimating LD based on short reads, a MAF of 0.15 was found to provide the most robust estimates of LD based on simulations (Feder et al. 2012 PLoS ONE). We now highlight this in the Methods (lines 192-194).

1.3 …The authors reported the shift of mating system and expansion on their effects on both the mutation load and LD, and proposed that LD and mutation load may have limited the species' range. However, in Introduction and Discussion, the authors mainly focused on LD. I think it may make the manuscript more complete if the authors could include some paragraphs on the effects of expansion and the shift of mating systems on mutation load, which had been a hot topic recently, for examples Peischl and Excoffier 2015 ME; Arunkumar et al. 2015 Genetics; Liu et al. 2017 MBE and Gaut et al. 2018 Nature Plants. It will be great if the author could also discuss the interactions among different factors.

The primary focus of our study is indeed on LD while we used the published estimates of genomic load to test if the latter is correlated with LD, which very likely has further implications on setting range limits. We discuss the implications of LD and load now already in the Introduction (lines 75-78, 121-122) and expanded the respective section in the Discussion (lines 507-512, 523-532).

Moreover, in our revised manuscript we show that LD between SNP pairs involving a putatively deleterious site increases with range expansion as much as between two putatively tolerated SNPs. As a consequence, selection may become

less effective at the range edge (please see our new Figure 2 and also our response to comment 3.0 by reviewer 3).

1.4 P6L116-117: This may be only true for the nearly complete selfing Arabidopsis thaliana, but not lyrata. For example, massive SVs were detected in grapevine and rice with similar Ne with lyrata.

No large-scale inversions were found in the initial reference genome assembly of *A. lyrata* subsp. *lyrata* (Hu et al. 2011 Nature Genetics), which we now also cite (lines 131-132). Although small inversions may exist, these do not necessarily have any selective value and are often polymorphic within and among populations (e.g., Lucek et al. 2019 Mol Ecol).

1.5 P7L144-147: Why the authors didn't use the current WGS data to calculate FIS?

While individual sequence data allow the estimation of FIS, estimates from pool-seq are less confident. We therefore kept the neutral marker estimates.

1.6 P13L278 and on: Please check the format of numbers.

We fixed the formatting of numbers throughout our manuscript.

1.7 P14L307: "and" is redundant here

Changed to "an" (line 398).

1.8 P21L488: Please update the data sharing.

All genomic data will be made publicly available upon acceptance.

1.9 Fig. 4 Why the numbers under X-axis wasn't evenly distributed?

We used a log-scale. We highlight this now in the figure legend (line 837, 869).

Comments by Reviewer 2:

2.1 A clear result is that distance to expansion centre and selfing rate tends to increase LD, as theoretically predicted. However, what is the next step from here? LD is higher in selfing populations (Fig 1) but they are also on the edge of the expansion range, so is there any interaction between selfing and expansion? Is this observation compatible with the supposed demographic model of expansion here?

Based on theory, we did not expect an interaction between expansion and a shift to selfing on LD. Nevertheless, we rerun models including the interaction and it was highly non-significant for both genic ($p=0.741$) and intergenic sites ($p=0.992$), which we now report on lines 300-302. In both cases the response variable was r2 and all other fixed effects and random effects structure were as for our first main model described in lines 282-299).

2.2 What is not clear to me is whether the LD partner is only a consequence of higher drift (+ reduced recombination for selfing) or whether there are more subtle effects through interaction with selection for example. Typically what can we conclude from Figure 2. A basic interpretation would be that higher drift induces a) lower diversity, b) lower selection on deleterious variant (so higher Pn/Ps) and c) higher linkage. Here the presentation of LD as a function theta Pn/Ps implicitly assumed that low theta and high load are among the causes of high LD. The comparison between genic and intergenic regions (Fig S2) could suggest interaction with selection but the interpretation is not clear.

As we highlight in our discussion, the overall genome-wide increase in LD for both genic and intergenic regions with range expansion is likely driven by drift (lines 445-447). By focusing on annotated genic SNPs in a second analysis (Table 2, new Figure 2) we further confirm that drift similarly affects LD among tolerated SNPs and among sites including putatively deleterious SNPs.

As we state in our discussion (lines 507-511), theta and load are only correlated with LD rather than causing it. Figure S2 was dropped from our revised manuscript.

2.3 Typically, what would really help to better understand the system would be simulation of range expansion with and without shift in mating system, and with and without background deleterious mutations. At least, this would give a baseline to better predict what we expect in the different populations.

Agreeably such a simulation approach could provide further insights on the effects of range expansion, mating system shift and an interplay of gene density on LD. However, given the complex demographic history of our study organism, such an endeavor would – when scaled down to trackable scenarios - likely provide only a simplistic view and seems to be better suited for future in-depth theoretical explorations of LD. We now highlight this gap in theoretical research in the discussion (lines 562-564).

2.4 The method to detect outliers is not very convincing. The rationale implicitly assumes that after controlling for mating system or distance, all else is equal so deviation for a given gene can be interpreted as a selective effect, and even more a selective effect due to selfing or expansion. I think this is clearly overstated. Many things could explain a departure from the global pattern, especially local variation in recombination rate. Once again, a more quantitative null model based on a demographic scenario would be more convincing to detect outliers. Some of outliers GO make sense but nothing is told about the others and one should be cautious about these GO outliers analyses (ex: Pavlidis P, Jensen JD, Stephan W, Stamatakis A. 2012. A critical assessment of storytelling: gene ontology categories and the importance of validating genomic scans. Mol Biol Evol, 29: 3237-48.) For example, another interpretation of higher diversity in outliers genes for mating system could be that because this genes have higher diversity, LD is better estimated and the relationship better supported whereas globally the noise reduces the slope of the relationship.

First, we clarified that we refer to LD outlier genes when genes showed a significant increase in LD after FDR and when the estimated coefficient was higher than the 95%CI from the genome-wide analysis (e.g. lines 30, 533-536). While we found no significant interaction between range expansion and mating system shift in the genome-wide analysis (see our comment to query 2.1), we decided to include this interaction in the gene-by-gene analysis and only report the genes that were solely affected by either factor. Indeed only one gene showed a significant interaction term following FDR (lines 418-419).

We also clarified throughout our manuscript that these strong statistical outliers may only be potential targets or linked to potential targets of selection by either factor or by other factors related to expansion or mating system shift (lines 447-450, 535-537). While we cannot directly show selection on these genes, we discuss some of the outliers in greater depth. These are genes known to be affected by ecological changes associated with range expansion (e.g. FPA and PDP2 – linked to flowering time and photoperiod respectively) or mating system shift (ARK3). We now highlight that the respective associations for these genes come actually from

the genus *Arabidopsis* (line 543, 546). However, we acknowledge that further in-depth studies are needed (lines 553-555), avoiding unnecessary story-telling.

2.5 P6 l116: I'm not sure to understand this rationale: increase in gene density -> lower recombination -> increase in LD?? Here a reference to a paper in Human is given but with a rather complex pattern (non monotonous) so it may not be true for all species.

We revised this section and motivate our inclusion of gene density based on observations in the closely related plant *Arabidopsis thaliana*, where gene density is negatively correlated with genetic polymorphism (Nordborg et al. 2005 PLOS Biol; see lines 129-131).

Comments by Reviewer 3:

3.0 If LD does increase then we could expect selection to become less efficient (Hil-Roberston effect) and this would also contribute to the difficulty to establish new populations far from the core and to set range limits…

We would like to thank the reviewer for the stimulating feedback. We included the discussion of the Hill-Robertson effect in our revised manuscript (line 499 onwards).

3.1 The authors estimated LD with two different approaches. In the pooled dataset they used r2, a classical measure of LD. In the resequencing dataset they used Delta, the correlation of zygosity across genic site, a much less common measure, developed by Haubold et al. (the paper describing the package MLRho used to estimate Delta has been cited 35 times since 2010). Of course, the low use of this statistics, does not mean that MLRho is not useful but it indicates that Delta is not commonly used and therefore that it needs to be described in more detail and its use justified a bit more than what the authors did. I must admit that it was not obvious to me what was gained by it. Furthermore, the use of both r2 and Delta (actually a transformed value of delta) makes the paper a bit harder to follow and it is not clear what the gain is. This needs to be better justified. Also in the case of full genome data would not coalescent-based methods estimating the population recombination rate along the genome (e.g. LDhat, LDhelmet) be more appropriate? Another difficulty I had when reading the paper is to kow which dataset was referred to. This is actually clearly stated in the M&M but was less clear, at least to this reader, in the Results section.

We had two data sets at hand, each with its own strengths and ability to answer some questions better than others. We used pool-seq data to get correlation coefficients between pairwise SNPs over short sequence lengths. The estimate of r2 is commonly used in the study of LD, is robust and allows for the easy comparison among studies. To quantify the sequence length over which LD unfolds, we used individual sequence data and the approach of the correlation of zygosity. Although the two estimates should be highly correlated, the latter can tell us directly over what length LD unfolds. We restructured the Methods and Results to more clearly say why we worked with different data sets and the two estimates, and what analysis was done with what data set (Methods: line 165 onwards, 218 onwards). Also, we describe delta in more depth (lines 224-231).

The two software the reviewer mentions, LDhat or LDhelmet, require many more individually re-sequenced genomes per population as shown by e.g. Hermann 2019 Mol. Ecol. Res or Adrion et al. 2020 Mol Evol Biol. However, we used a novel tool that implements machine learning and also works with pool-seq data (Adrion et al. 2020 Mol. Evol. Biol.). We show that in our studied sections of the genome LD and

recombination only very loosely associated (Figure S3, lines 363-366) and did therefore not include recombination in our model (see also lines 251-255).

3.2 As expected the authors did find a relationship between LD and population expansion and the shift to selfing and similarly also a relationship between mean LD and Ne or genetic load. One major issue with the paper is that it does not really go beyond correlation and the discussion on the limits to range expansion created by higher LD remains rather speculative. In other words the article perhaps lacks some theoretical underpinning that could have helped to interpret these correlations. Perhaps more could have been achieved with considering the distribution of LD (or Rho) and variation along the genome?

In summary, the study seems to have been done carefully, the topic is not devoid of interest, the paper is well written and there have not been many studies of that type. On the other hand, I was left with a lingering frustration that the study did not make the results put forward in the abstract as clear as one would have wished and I think I would have preferred a more thorough study and less correlative study based solely on the 104 genomes.

In response to this comments, we revised the theory part of the Introduction and extended it (lines lines 66-78, 87-95, 102-109; also see response to comment 1 by Dr. Bomblies).

Furthermore, we tried to draw tighter links between patterns by annotating SNPs in genes using SIFT, repeated analyses on LD, and found that an increase in LD due to expansion and selfing is the same for tolerated and deleterious SNPs (Table 2, new Figure 2).

We discuss the potential implications using two theoretical frameworks, including the Hill-Robertson effect, which may reduce the efficacy of selection and be one factor to set range limits (lines 517-523).

We revised the Abstract to make clearer what was found.

Again, we would like to thank you for your handling of the manuscript.

Sincerely, Kay Lucek, Yvonne Willi.
