# Supplementary material for: Drivers of linkage disequilibrium across a species’ geographic range
Source: PLoS Genet. 2021 Mar 26;17(3):e1009477. doi: 10.1371/journal.pgen.1009477 (PMC8026057; doi:10.1371/journal.pgen.1009477)
Supplement: Attachment — Submitted filename: Response_letter.docx [file pgen.1009477.s013.docx]

Revised manuscript PGENETICS-D-20-01727

Dear Dr. de Meaux, dear Dr. Bomblies

We would like to thank you and the reviewers for the constructive comments on our manuscript. Below we copied the comments and write how we addressed them in our manuscript in blue.

Comments by the editors:

I would like to suggest you refer to Takou et al.  <https://www.biorxiv.org/content/10.1101/709873v4>, which provides a comparison of LD in an equilibrium and a bottlenecked population in the related subspecies A. lyrata ssp. petraea. This data is more exhaustive than ref. 31. Please also revise Line 441: it seems to me that the factors that drive LD are known, but their relative importance in nature is seldom described.
*We kept ref. 31 as we cite that paper in the context of comparing selfing with outcrossing taxa. Takou et al. did not seem appropriate to cite as they compared three outcrossing populations of A. lyrata ssp. petraea. However, we thank the editors for pointing us to this novel contribution, which we now cite in lines 474-476. We also revised the suggested section of our discussion (lines 446-447).*

Comments by reviewer 2:

Page 22, 511-512: I am far from certain that LD is a good predictor of mutation load and of a drift-syndrome (whatever the latter means).

*We clarified this section accordingly (lines 516-518).*

Page 23: line 528-529: This conclusion seems rather strong.

*We toned down the conclusion (lines 532-536).*

Comments by reviewer 3:

P4 L57: About the effect of bottleneck on LD, in Schaper et al. (2012) the authors not only showed that bottleneck can increase the extend of LD but that, under certain conditions at least, make LD less dependent to physical distant so generate LD at higher distance than expected by a simple Ne rescaling. I don’t think this prediction can be easily tested but at least it should be mentioned. Maybe one possibility would be to compare the observe pattern of decrease in LD with physical distance between SNPs with a simple neutral model. For ex on Fig 1C and 1D it is not clear how the curves have been obtained. What is the expected shape under neutrality and equilibrium? I’ve noted that the authors justify not too run complex simulations but at least comparison with neutral expectations is easily doable.

*We carefully reconsidered Schaper et al. (now cited in lines 56-59 and 68-69). Authors modeled LD at two loci on two chromosomes using a Wright–Fisher model. In contrast, our study is based on empirical data with hundreds of thousands of SNPs, and we deal with a complex biogeographic history within the species (2 distinct glacial refugia, range expansion, mating system shifts, etc.). These complexities make it hard to simulate accurate predictions of LD for comparison. Therefore, we aimed for the comparison of LD with core populations that have high genetic diversity and should be closest to an equilibrium state. This has been the preferred approach in many empirical studies, e.g. in Takou et al. (see comment to editors). Moreover, Figure 1C & D shows the correlation of zygosity for genic (D) and intergenic (C) portions of the A. lyrata genome, the latter being commonly used as a proxy for neutral expectations.*

The recombination map is a nice addition but could have been exploited more.
- First some points should be precised. Was the map estimated using the four core populations pooled or separated? If the population structure is weak among them, they can be pooled otherwise it is better to perform distinct estimates. If distinct estimates have been obtained how are they averaged?

*Population genetic structure in A. lyrata is rather strong (e.g. Lucek et al. 2019 Mol Ecol, Walden et al. 2020 Mol Ecol), and we therefore estimated the average recombination rate for each population and each genic and intergenic region separately. We clarified this (lines 251-255).*

- The authors noted no correlation between LD and local recombination rate, which is a bit weird as recombination is based on LD information. A possible reason is that they only consider recombination estimate at a very small scale where precision can be low.

*As we highlight in the methods section (lines 249-251), ReLERNN estimates recombination rates in self-set bins. The size of these bins differs among populations (IA1: 137’500 bps, MD4: 63’100 bps, NY1: 61’100 bps, WI1: 61’400 bps). In contrast, our LD estimates from poolseq data were estimated between SNPs within the same paired reads, i.e. within 500bps of distance. This discrepancy could account for the absence of a correlation, however, in the absence of population scale individual sequences, we cannot confirm this.*

- It would be interesting to have a smoother view of recombination map (in addition to Fig S3). For example it seems that there are genomic regions (center of chromosomes?) with lower recombination rates.

*We revised our Figure S3, indicating now the centromeric regions and the smoothed average recombination across the genome.*

- It is not clear also whether the correlation have been done only in the four core populations? What would be important to know is whether the increase in LD observed in peripheral and selfing populations is more pronounced in regions of low recombination (which can be categorized at a larger scale than done here).

*The reviewer is correct; to assess the relationship between LD and recombination rates we used Pearson product moment correlations between the two estimates for the four populations for which we estimated recombination. We clarified this in our revised manuscript (lines 253-255). We thus correlated the actual average LD and average recombination rate estimated from the same data and the very same genomic regions. As we found only a weak correlation between LD and recombination (Figure S3), we did not consider recombination in our subsequent analyses. Also, given the very weak correlation within a population, we did not correlate the recombination estimates with LD estimates from other populations.*

They may have some issues with the linear model analysis. If I understood correctly a classical mixed linear model has been used. However, r2 is not a normal variable (0≤r2≤1) so at least a transformation is required. In addition, the mean r2 over 300 bp is used as the dependent variable. The precision on the estimate of this variable depends on the number of pairs of SNPs used to estimate it. Because this number increases with local diversity, the variance should also do it, which violate the heterosedasticity hypothesis. Linear model are usually quite robust to such violation but here it goes in the direction of the explanatory variables. This should be checked and alternative model should be used is necessary.

*For all our main analyses presented in Tables 1 & 2 of our manuscript we used linear mixed effect models implemented in the R package lme4 (lines 281-284). Because we found LD to generally decay with increasing distance between loci (Supplementary Information 2), we calculated the residuals of a linear model between LD (r2) and the log_10_-transformed base pair distance between loci across all retained LD estimates (see lines 202-205). r2, the dependent variable of our main models was therefore already transformed. Furthermore, we checked for normality and homoscedasticity of residuals of our models by diagnostic plots (see figures 1&2 below and lines 311-312) and results supported their okay distribution.*

*Figure 1: Quantile-quantile plots for the residuals of our linear mixed effects models based on LD estimates of: A genic, B intergenic and C SIFT annotated SNPs.*

*Figure 2: Diagnostic plots for the residuals of our linear mixed effects models based on LD estimates from: A genic, B intergenic and C SIFT annotated SNPs, comparing the Pearson residuals (scaled by the variance function) with the fitted residuals.*

P13 L292-294: Instead of Fis, the RMES software could be used to get more accurate estimates of the selfing rates (David, P., B. Pujol, F. Viard, V. Castella, and J. Goudet. 2007. Reliable selfing rate estimates from imperfect population genetic data. Mol. Ecol. 16:2474-2487.).

*We checked RMES. Unfortunately the software requires population scale individual data, which does not apply for our genomic datasets. Also, a former study (Griffin & Willi 2014 Ecol Lett) showed that Fis and outcrossing rates that were experimentally assessed by progeny arrays provide almost identical estimates (R2=0.929).*

P17 L383-386: Analysis of SIFT data. The authors found a (slight) difference between the LD between the different SIFT categories. However, they should have different frequencies, lower for DEL than TOL SNPs. This could explain the difference between the categories. This could be checked by redoing the analysis for different classes of frequencies. In Table 2, I don’t understand how a single effect can be obtained for the SIFT type given that there are three categories. This variable should be categorical with two values for the effect.

*LD estimates were calculated between 3 pairs of SNPs, TOL-TOL, TOL-DEL and DEL-DEL, and therefore there are three classes for the effect of SIFT type. Furthermore, the reviewer is right that replicate numbers within classes differed, as we stated in our manuscript (lines 309-311). However, this should not be a problem as mixed model analyses are rather robust when data is unbalanced. We further inspected the diagnostic plots of our model (see Fig. 1C, Fig. 2C above), which were similar to the overall models.*

*To check for consistency in the contribution of interaction terms involving SIFT type, we repeated our analysis omitting all DEL-DEL estimates. The resulting marginal effect plots (Figure 3 below) show the same patterns as when DEL-DEL estimates were included (Figure 2 in our manuscript): The interaction between SIFT-SNP type and expansion distance becomes non-significant (Table 1 below), suggesting that the DEL-DEL estimates were responsible for the overall significant interaction as we observed in our manuscript (Figure 2 in our manuscript). The interaction between SIFT-SNP type and mating system remains significant, although to a much lesser degree, which also becomes obvious from the marginal effects plot (Figure 3 below). Thus, the conclusions of our manuscript remain.*

*Figure 3:* *Marginal effects for the interactions of LD (r^2^) estimated from pool-seq between variants that were identified by SIFT to be both deleterious, deleterious and tolerated, or both tolerated, and A – expansion distance (km – log-scale) and B - mating system (F_IS_). Regression lines and their 95% confidence intervals were estimated from a linear mixed effect model.*

*Table 1: Results of the linear mixed-effects model with genome-wide linkage disequilibrium (LD) using only distance-corrected r^2^ between pairs of SNPs in genic regions as the respective dependent variable that were annotated by SIFT. For each fixed effect the model estimate (β), as well as the chi-square statistics (χ^2^) with the associated p value from a type III Wald χ^2^ tests are given. Only results for fixed effects are shown.*

|  | ***LD (r^2^)*** | | |
| --- | --- | --- | --- |
| **Fixed effect** | ***β* ± SE** | **χ^2^** | ***P*** |
| Genetic cluster (east) | 0.00592 ± 0.00437 | 1.833 | 0.1758 |
| Log_10_ expansion distance | 0.03966 ± 0.00947 | 17.428 | <0.0001 |
| Mating system (*F*_IS_) | 0.02617 ± 0.00846 | 12.185 | 0.0005 |
| Log_10_ distance to adjacent genes | -0.01184 ± 0.00111 | 112.827 | <0.0001 |
| SIFT-SNP type | -0.00019 ± 0.00182 | 9.450 | 0.0021 |
| Log_10_ expansion distance * SIFT-SNP type | -0.00023 ± 0.00070 | 0.110 | 0.7402 |
| Mating system (*F*_IS_) * SIFT-SNP type | 0.00380 ± 0.00083 | 20.800 | <0.0001 |

Again, we would like to thank you for the fast handling of your manuscript, and we are looking forward to your response.

Sincerely, Kay Lucek.
